# Supplementary material for: Predictive Microbial Markers Distinguish Responders and Non-Responders to Adalimumab: A Step Toward Precision Medicine in Ulcerative Colitis
Source: Microorganisms. 2025 Aug 20;13(8):1941. doi: 10.3390/microorganisms13081941 (PMC12388438; doi:10.3390/microorganisms13081941)
Supplement: Supplementary file 1 [file microorganisms-13-01941-s001.zip › microorganisms-3744924-SI.pdf]

Supplemetary materials

Supplementary Figure S1. The abundance of the four major phyla in the stool microbiomes (upper rows) or from mucosa biopsies (lower row) from ulcerative colitis (UC) patients with a flare-up of inflammation was assessed by endoscopy (Mayo index of 3). The box displays the interquartile range (IQR 25-75%). The line inside the box represents the median of the values, while the whiskers indicate values within 1.5 times the IQR. The circles outside the whiskers represent the outliers. The upper rows represent data from stool microbiomes [23, 21, 15 samples, respectively], while the lower rows [16, 14, 15 samples, respectively] show the microbiomes from tissue biopsies. The number in square brackets indicates the number of patients that belong to the groups compared in the analysis.

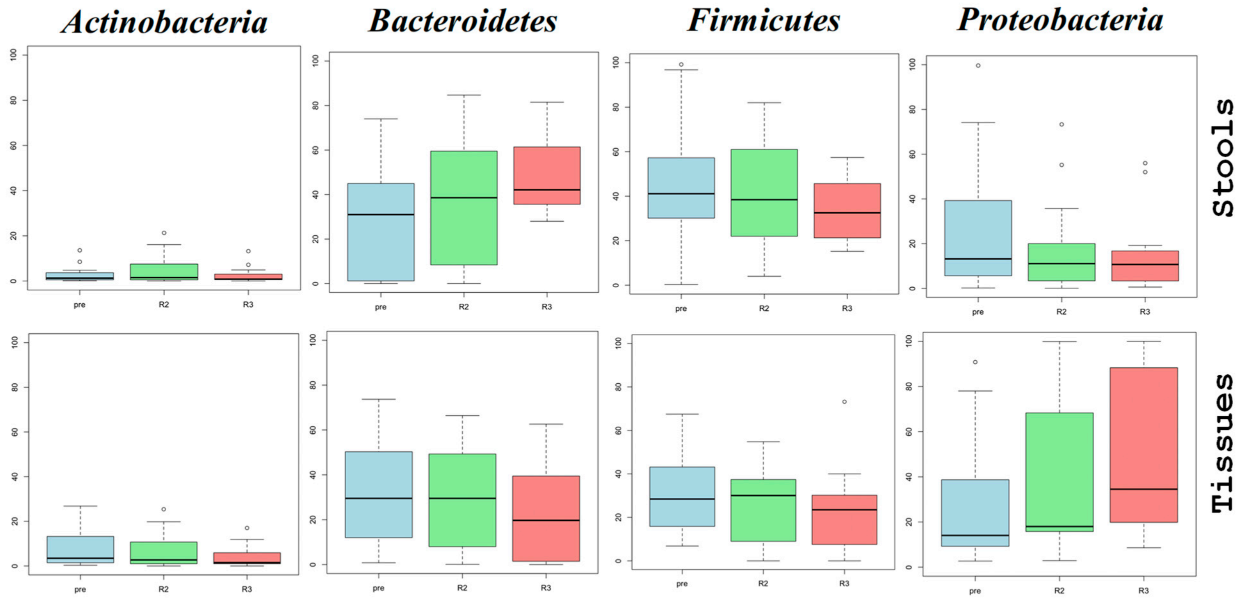

Supplementary Figure S2: The Chao 1, Shannon, and Simpson alpha diversity indexes derived from stool or mucosal tissue biopsies of the microbiome in ulcerative colitis (UC) patients. This study conducts a longitudinal microbiome analysis of UC patients who responded to anti-TNFα treatment based on the endoscopic Mayo score index. Samples before therapy (pre) have a Mayo score of 3, Non-Responders (NR) Mayo score >=2, and Responders (R) Mayo score <=1. In the upper row, microbiome data from stool samples are illustrated, with samples collected before the onset of anti-TNFα treatment (pre) [23] and the Non-Responders samples (NR) [13] or the Responders (R) [23] of therapy. The lower row provides a corresponding analysis of microbiome samples from inflamed gut mucosal tissues, with data from (pre) [16], NR [7], and R [22]. The numbers in square brackets indicate the sample size for each group. The richness Chao 1 index is displayed on the left, while the Shannon and Simpson indexes, which represent the richness and evenness, are shown in the middle and on the right, respectively.

Supplementary Figure S2

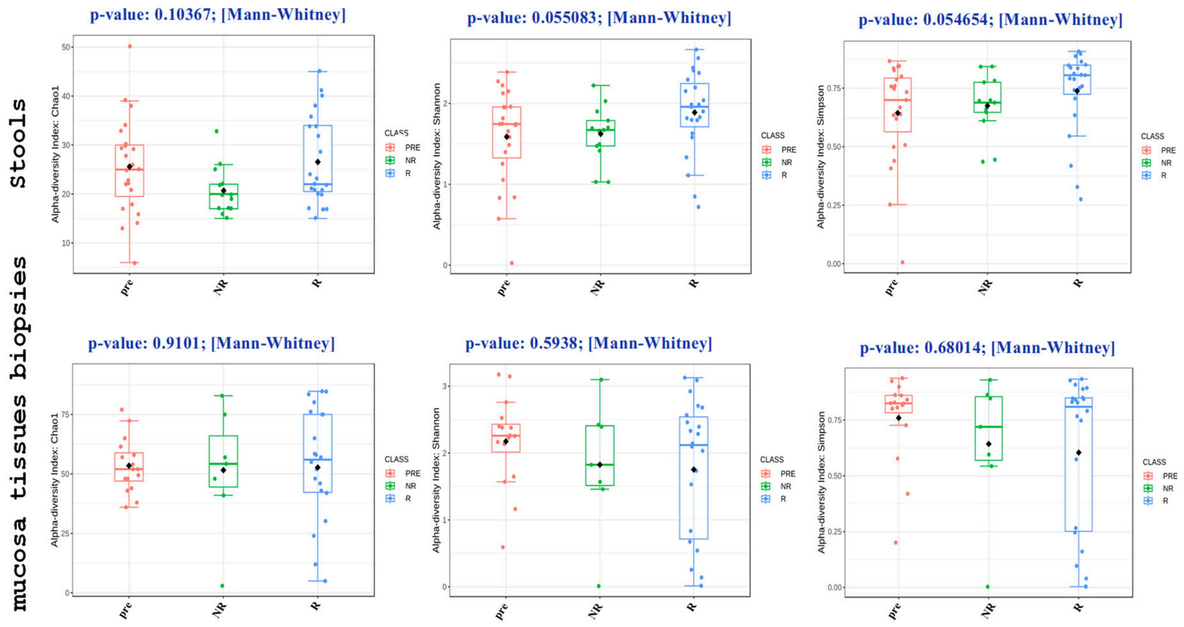

Supplementary Figure S3: Bray-Curtis beta diversity using PCoA (Principal Coordinate Analysis) plots to visualize results in Cinnora®-treated samples.

Stool (left) and Mucosa (right) microbiomes among Cinnora® non-responders (NR), before any treatment (pFu), and responders (R) sample groups. Statistical analysis was performed using overall PERMANOVA and pairwise analysis.

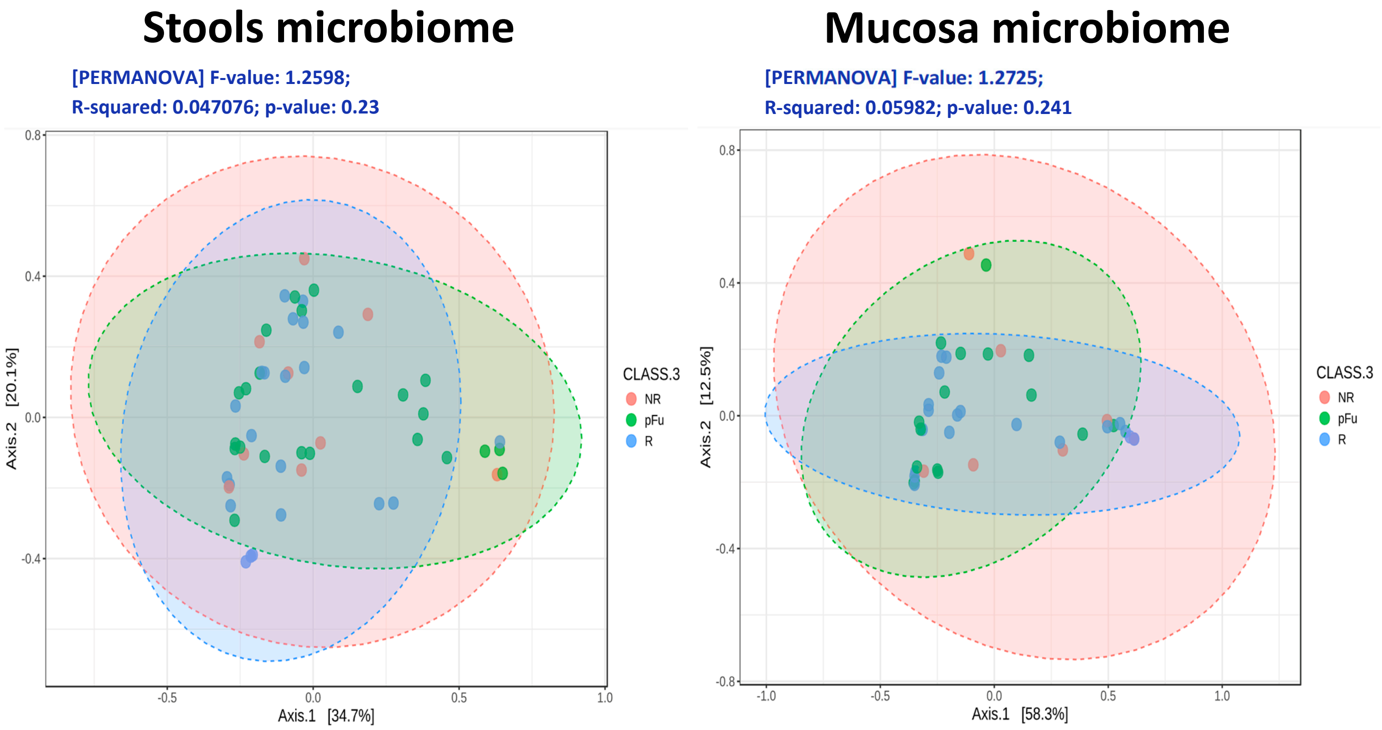

Stool pairwise analysis

Pair ⇅	F-value ⇅	R-squared ⇅	P-value ⇅	FDR ⇅
pFu vs R	2.0866	0.04628	0.066	0.198
pFu vs NR	0.41554	0.013662	0.868	0.868
R vs NR	0.96222	0.032114	0.446	0.669

Mucosa pairwise analysis

Pair ⇅	F-value ⇅	R-squared ⇅	P-value ⇅	FDR ⇅
pFu vs R	1.5475	0.043532	0.195	0.3165
pFu vs NR	1.3993	0.062472	0.211	0.3165
R vs NR	0.81703	0.031647	0.39	0.39

Supplementary Figure S4: Longitudinal results of the abundance of the microbial families between two classes, left-sided colitis vs. Pancolitis stool samples. This figure represents longitudinal results comparing the abundance of microbial families between two classes: left-sided colitis and pancolitis stool samples. It includes the following plots: (i) the receiver operating characteristic (ROC) curve, (ii) the per-class ROC, (iii) a confusion matrix comparing true labels with predicted labels, and (iv) a heatmap illustrating the relative abundance of the most discriminating families between the two classes. In the heatmap, subjects experiencing flare-ups are labeled with "F," while those in remission are labeled with "R."

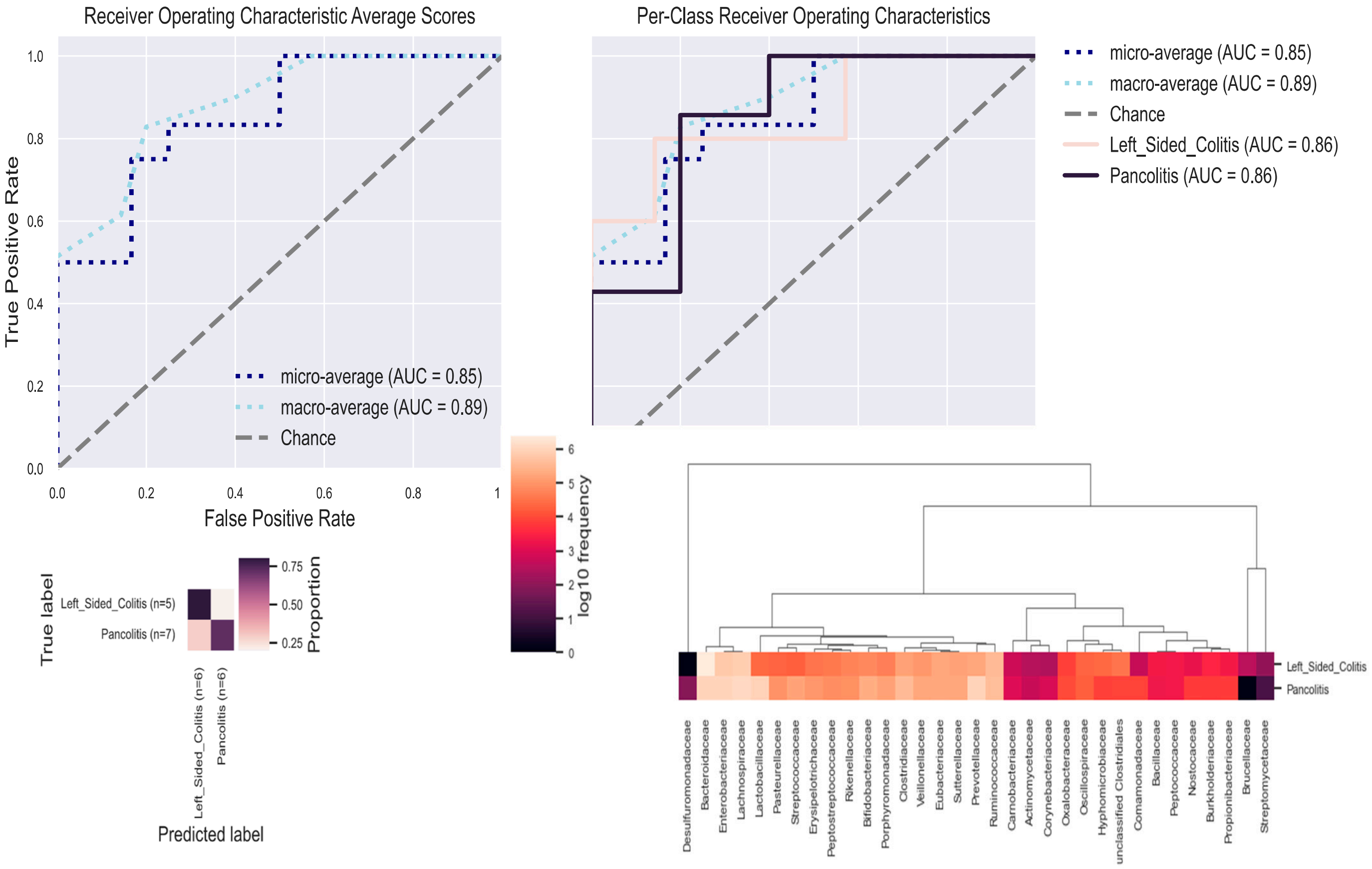

Supplementary Figure S5: Longitudinal results of the species abundance between two classes, Flare up vs. Remission stool samples. This figure presents the longitudinal results of species abundance between two classes: Flare-up and Remission stool samples. It includes the following plots: (i) the receiver operating characteristic (ROC) curve, (ii) the per-class ROC, (iii) the confusion matrix comparing true labels versus predicted labels, and (iv) a heatmap depicting the relative abundance of the most discriminating species between the two classes. In the heatmap, subjects identified as experiencing a flare-up are labeled with “F,” while those in remission are labeled with “R.”

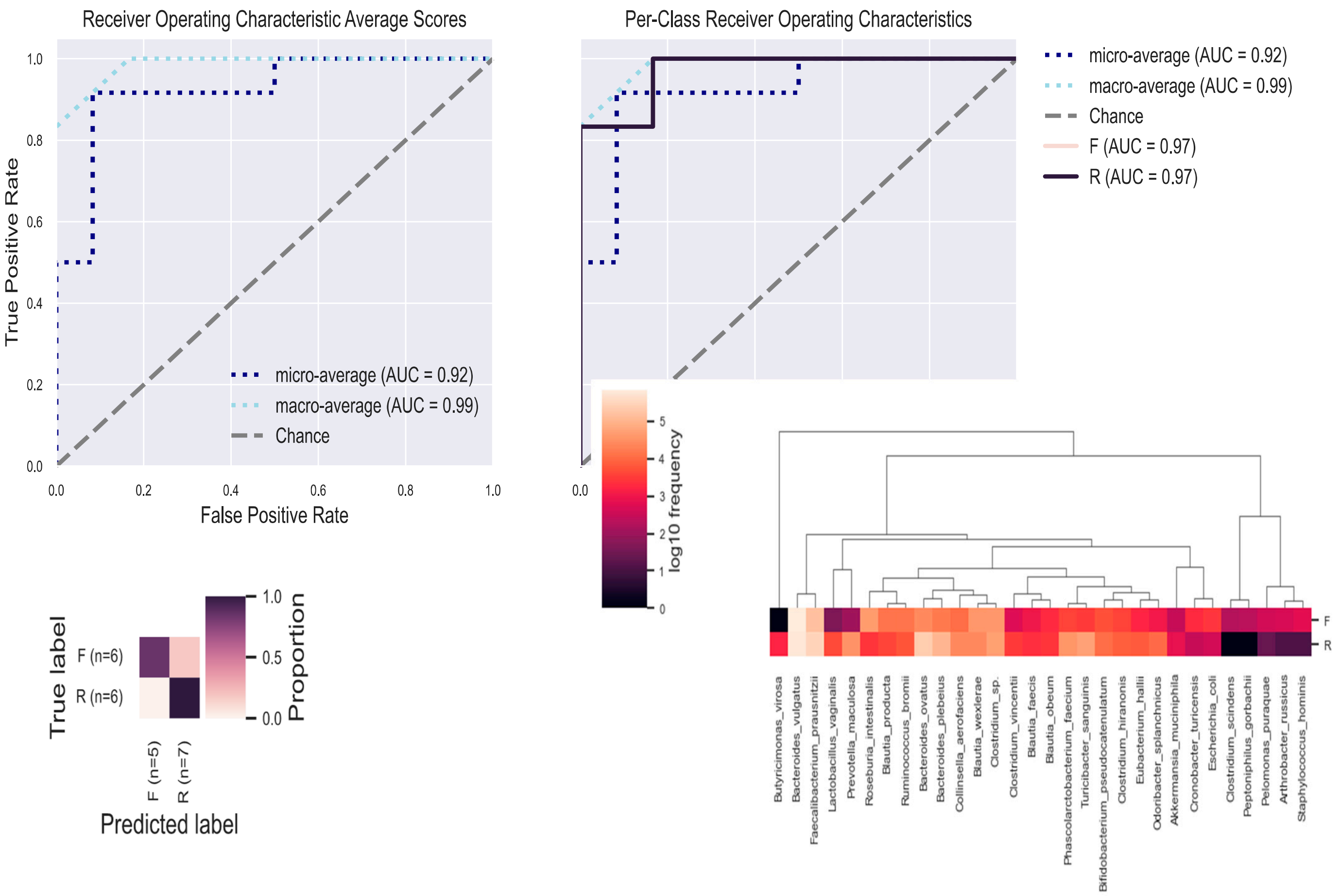

Supplementary Figure S6a: Weighted Correlation Network Analysis (WGCNA) in the Stool Microbiome of non-responsive patients to anti-TNF alpha treatment: Exploring Correlations Between Microbial species and Clinical Variables, Significance of module-trait relationships is determined using p-values and adjusted for multiple comparisons using the False Discovery Rate (FDR). The phylum classification for each microbial species is indicated in parentheses: (A) *Actinobacteria*, (B) *Bacteroidetes*, (F) *Firmicutes*, (P) *Proteobacteria*, and (V) *Verrucomicrobiae*. The evaluation criteria include the following variables: - Age: continuous variable - Sex: Male (1), Female (0) - Age at diagnosis: continuous variable - Disease duration in years: continuous variable - Extension of disease before therapy: Left-Sided Colitis (1), Pancolitis (0) - Severity of disease before therapy: Severe (1), Mild to Moderate (2), Moderate (3), Moderate to Severe (4) - Endoscopic activity index of the rectum after 3 months of Cinnora® therapy: Remission (0), Flare-up (1) - Disease state 6 months after Cinnora® therapy: Remission (0), Flare-up (1) - Corticosteroids: Dependency (1) or corticosteroid refractoriness (0) - Azathioprine: Dependency (1) or not (0) - Comorbidities (primary sclerosing cholangitis, rheumatoid arthritis, and pyoderma gangrenosum): Yes (1), No (0) –
Alcohol habits: Yes (1), No (0) - Smoking habits: Yes (1), No (0).

Stools microbiome from UC patients Non-responsive to the therapy

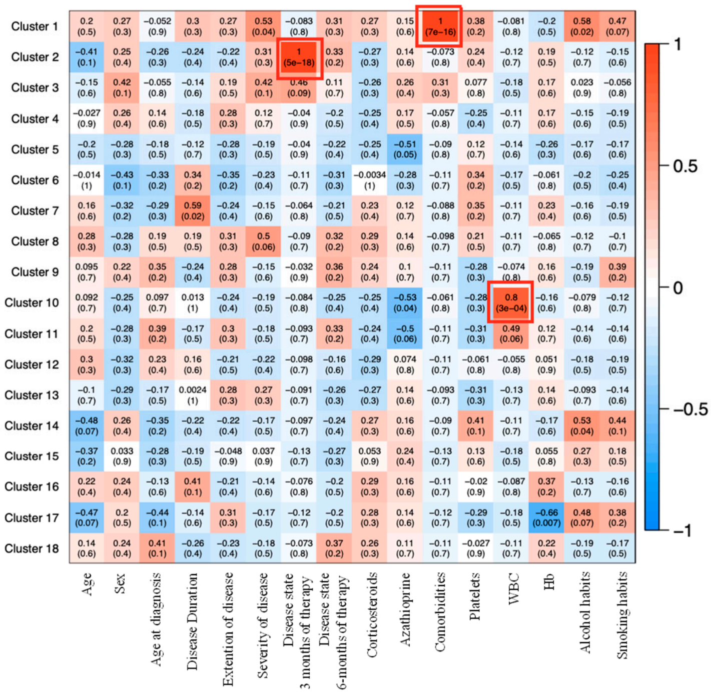

Co-morbidies_ Cluster 1
(Positive correlation:1; FDR:1.38e-15):
Abiotrophia defective (F), Actinomyces naeslundii (A), Actinomyces odontolyticus (A), Corynebacterium argentoratense (A), Enterococcus faecium (F), Enterococcus hirae (F), Enterococcus lactis (F), Enterococcus sp. (F), Enterococcus villorum (F), Eschericia/Shigella coli.dysenteriae (P), Granulicatella adiacens (F), Granulicatella elegans (F), Kocuria kristinae (A), Streptococcus australis (F), Streptococcus parasanguinis (F), Streptococcus sinensis (F), Veillonella rogosae (F).

Disease state after 3-months of therapy _ Cluster 2
(Positive correlation:1; FDR:1.02e-13):
Acidaminococcus fermentans (F), Acidaminococcus sp. (F), Allisonella histaminiformans (F), Bacteroides clarus (B), Catenibacterium mitsuokai (F), Coprococcus catus (F), Corynebacterium amycolatum (A), Cronobacter turicensis (P), Eubacterium biforme (F), Eubacterium biforme (F), Howardella ureilytica (F), Lactobacillus mucosae (F), Lactobacillus ruminis (F), Olsenella sp. (A)

WBC_Cluster 10 (Positive correlation:0.8; FDR:0.03):
Bacteroides plebeius (B), Blautia torques (F), Clostridium butyricum (F), Clostridium glycolicum (F), Clostridium hiranonis (F), Clostridium mayombei (F), Lactobacillus fermentum (F), Lactobacillus salivarius (F), Prevotella copri (B), Roseburia inulinivorans (F), Streptococcus salivarius (F), Sutterella wadsworthensis (P), Veillonella atypica (F), Veillonella denticariosi (F),Veillonella dispar (F).

Supplementary Figure S6b: Weighted Correlation Network Analysis (WGCNA) in the Inflamed tissues Microbiome of patients Non-responding to anti-TNF alpha treatment: Exploring Correlations Between Microbial species and Clinical Variables, Significance of module-trait relationships is determined using p-values and adjusted for multiple comparisons using the False Discovery Rate (FDR). The phylum classification for each microbial species is indicated in parentheses: (A) *Actinobacteria*, (B) *Bacteroidetes*, (F) *Firmicutes*, (P) *Proteobacteria.*

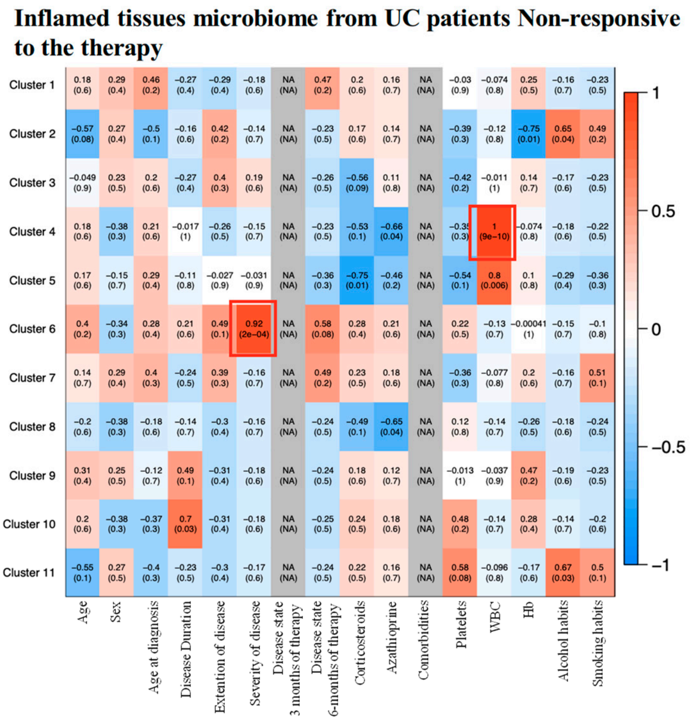

WBC_Cluster 4 (Positive correlation:1; FDR:0.01):
Actinobacillus lignieresii (P), Actinomyces dentalis (A), Alpinimonas psychrophile (A), Anaerofustis stercorihominis (F), Anaerotruncus colihominis (F), Aquaspirillum putridiconchylium (P), Blautia obeum (F), Bosea thiooxidans (P), Burkholderia bryophila (P), Butyricimonas virosa (B), Capnocytophaga ochracea (B), Cellvibrio fibrivorans (B), Corynebacterium maris (A), Corynebacterium matruchotii (A), Flavobacterium aquidurense (B), Flavobacterium sp. (B), Flavobacterium succinicans (B), Megasphaera elsdenii (F), Microvirga aerophile (P), Microvirga lupini (P), Parabacteroides distasonis (B), Paraprevotella clara (B), Phascolarctobacterium faecium (F), Pseudoflavonifractor capillosus (F), Rhizobacter fulvus (P), Rhizomicrobium electricum (P), Rothia mucilaginosa (A), Senegalimassilia anaerobia (A), Staphylococcus pasteuri (F), Streptococcus australis (F), Streptococcus pseudopneumoniae (F), Succinivibrio dextrinosolvens (P), Tepidimonas fonticaldi (P).

Severity of disease_Cluster 6 (Positive correlation:0.92; FDR:1.55e-7):
Asticcacaulis excentricus (P), Bacillus decisifrondis (F), Bifidobacterium longum (A), Blautia wexlerae (F), Campylobacter hominis (P), Clostridium paraputrificum (F), Clostridium perfringens (F), Cronobacter malonaticus (P), Cronobacter turicensis (P), Enhydrobacter aerosaccus (P), Enterobacter asburiae (P), Escherichia coli (P), Escherichia vulneris (P), Flectobacillus roseus (B), Herbaspirillum rhizosphaerae (P), Kocuria palustris (A), Leclercia adecarboxylata (P), Methylobacterium jeotgali (P), Microbacterium ginsengisoli (A), Pantoea agglomerans (P), Peptoniphilus lacrimalis (F), Prevotella bergensis (B), Prevotella bivia (B), Prevotella disiens (B), Salmonella enterica (P), Sporobacterium sp. (F), Staphylococcus epidermidis (F), Trabulsiella odontotermitis (P).

Supplementary Figure S7a: Weighted Correlation Network Analysis (WGCNA) in the **stool Microbiome** of patients responding to anti-TNF alpha treatment: Exploring Correlations Between Microbial species and Clinical Variables, Significance of module-trait relationships is determined using p-values and adjusted for multiple comparisons using the False Discovery Rate (FDR). The phylum classification for each microbial species is indicated in parentheses: (A) *Actinobacteria*, (B) *Bacteroidetes*, (F) *Firmicutes*, (P) *Proteobacteria*, and (V) *Verrucomicrobiae*. The evaluation criteria include the following variables: - Age: continuous variable - Sex: Male (1), Female (0) - Age at diagnosis: continuous variable - Disease duration in years: continuous variable - Extension of disease before therapy: Left-Sided Colitis (1), Pancolitis (0) - Severity of disease before therapy: Severe (1), Mild to Moderate (2), Moderate (3), Moderate to Severe (4) - Endoscopic activity index of the rectum after 3 months of Cinnora® therapy: Remission (0), Flare-up (1) - Disease state 6 months after Cinnora® therapy: Remission (0), Flare-up (1) - Corticosteroids: Dependency (1) or corticosteroid refractoriness (0) - Azathioprine: Dependency (1) or not (0) - Comorbidities (primary sclerosing cholangitis, rheumatoid arthritis, and pyoderma gangrenosum): Yes (1), No (0) - Platelet count: continuous variable - White Blood Cell (WBC) count: continuous variable - Hemoglobin (Hb): continuous variable - Alcohol habits: Yes (1), No (0) - Smoking habits: Yes (1), No (0).

Stools microbiome from UC patients responsive to the therapy

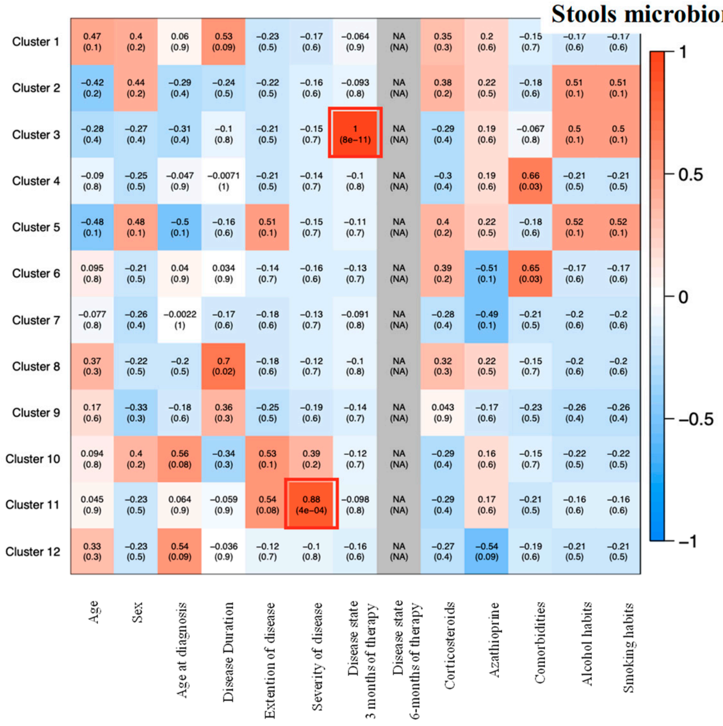

Disease state after 3-months of therapy _ Cluster 3 (Positive correlation: 1; FDR:0.03):
Alistipes putredinis (B), *Bacteroides eggerthii* (B), *Bifidobacterium longum* (A), *Enterobacter sacchari* (P), *Enterococcus casseliflavus* (F), *Enterococcus faecium* (F), *Enterococcus mundtii* (F), *Granulicatella adiacens* (F), *Granulicatella elegans* (F), *Hafnia alvei* (P), *Klebsiella pneumoniae* (P), *Klebsiella variicola* (P), *Lactobacillus amylovorus* (F), *Lactobacillus kefiranofaciens* (F), *Lactobacillus kitasatonis* (F), *Lactobacillus mucosae* (F), *Morganella morganii* (P), *Parabacteroides distasonis* (B), *Parabacteroides merdae* (B), *Parasutterella excrementihominis* (P), *Pediococcus acidilactici* (F), *Pediococcus lolii* (F), *Phascolarctobacterium faecium* (F), *Propionibacterium acnes* (A), *Ruminococcus lactaris* (F), *Serratia symbiotica* (P), *Shimwellia blattae* (P), *Streptococcus australis* (F), *Streptococcus peroris* (F), *Streptococcus pseudopneumoniae* (F), *Streptococcus sinensis* (F), *Streptococcus thermophilus* (F).

Severity of disease _Cluster 11 (Positive correlation: 0.88; FDR:1.20e-08):
Akkermansia muciniphila (V), *Bacteroides finegoldii* (B), *Bacteroides ovatus* (B), *Bacteroides thetaiotaomicron* (B), *Eubacterium ramulus* (F), *Eubacterium sp.* (F), *Eubacterium ventriosum* (F), *Lactobacillus delbrueckii* (F), *Leclercia adecarboxylata* (P), *Odoribacter splanchnicus* (B), *Roseburia hominis* (F), *Ruminococcus champanellensis* (F), *Subdoligranulum variabile* (F).

Supplementary Figure S7b: Weighted Correlation Network Analysis (WGCNA) in the **tissue biopsies Microbiome** of patients responding to anti-TNF alpha treatment: Exploring Correlations Between Microbial species and Clinical Variables, Significance of module-trait relationships is determined using p-values and adjusted for multiple comparisons using the False Discovery Rate (FDR). The phylum classification for each microbial species is indicated in parentheses: (A) *Actinobacteria*, (B) *Bacteroidetes*, (F) *Firmicutes*, (P) *Proteobacteria*, and (V) *Verrucomicrobiae*. The evaluation criteria include the following variables: - Age: continuous variable - Sex: Male (1), Female (0) - Age at diagnosis: continuous variable - Disease duration in years: continuous variable - Extension of disease before therapy: Left-Sided Colitis (1), Pancolitis (0) - Severity of disease before therapy: Severe (1), Mild to Moderate (2), Moderate (3), Moderate to Severe (4) - Endoscopic activity index of the rectum after 3 months of Cinnora® therapy: Remission (0), Flare-up (1) - Disease state 6 months after Cinnora® therapy: Remission (0), Flare-up (1) - Corticosteroids: Dependency (1) or corticosteroid refractoriness (0) - Azathioprine: Dependency (1) or not (0) - Comorbidities (primary sclerosing cholangitis, rheumatoid arthritis, and pyoderma gangrenosum): Yes (1), No (0) - Platelet count: continuous variable - White Blood Cell (WBC) count: continuous variable - Hemoglobin (Hb): continuous variable - Alcohol habits: Yes (1), No (0) - Smoking habits: Yes (1), No (0).

Inflamed tissues microbiome from UC patients responsive to the therapy

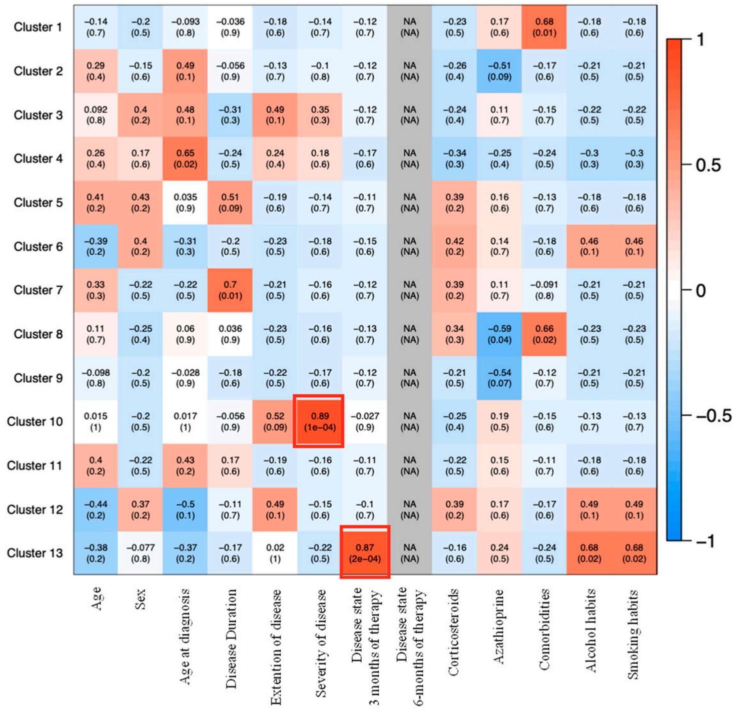

Severity of disease _ **Cluster 10**
[Positive correlation: 0.89; FDR:0.02]:
Asticcacaulis excentricus (P), *Enterobacter hormaechei* (P), *Escherichia/Shigella coli* (P), *Escherichia coli* (P), *Mangrovibacter plantisponsor* (P), *Methylobacterium aquaticum* (P), *Neisseria subflava* (P), *Prevotella nanceiensis* (B), *Rheinheimera sp.* (P), *Roseburia intestinalis* (F), *Shigella boydii* (P), *Sphingomonas echinoides* (P), *Sphingomonas ginsenosidimutans* (P), *Sphingomonas koreensis* (P).

Disease state after 3-months of therapy _ **Cluster 13**
[Positive correlation: 0.87; FDR:0.02]:
Cronobacter malonaticus (P), *Enterobacter asburiae* (P), *Enterobacter sp.* (P), *Escherichia vulneris* (P), *Leclercia adecarboxylata* (P), *Stenotrophomonas geniculate* (P).

Supplementary Table S1.

Extension of pathology, relative abundance analysis on the intestinal biopsies microbiome of Ulcerative Colitis affected patients before any recent therapy.

This study examines the extension of pathology through microbiome relative abundance analysis on intestinal biopsies from patients with inflamed mucosal tissues due to Ulcerative Colitis (UC). Data analyses were conducted on patients experiencing flare-ups of UC (pFu) at the time of sampling before the initiation of CinnoraR therapy. The number in square brackets indicates the number of patients that belong to the groups compared in the analysis. The complete taxonomy is shown on the left column (p_Phylum; c_Class; o_Order; f_Family; g_Genus; s_Species). Relative abundance analysis was performed with zero-inflated Gaussian fit statistical method, the EdgeR or the DESeq2 algorithms. FDR (False Discovery Rate) indicates the statistical significance “p-value” after adjustment for multiple comparisons. The base two logarithmic value of fold changes (log2FC) represents how much the value of increase or decrease of abundance of a particular taxon is in the comparisons between the two groups of samples. FDR values equal to or less than 0.05 were considered statistically significant.

Taxonomy	Left sided colitis [6] *vs* Pancolitis [10]	zero-inflated Gaussian fit	EdgeR		DESeq2	
		FDR	log2FC	FDR	log2FC	FDR
**Higher abundance in UC patients diagnosed for Pancolitis**						
p_Actinobacteria; c_Actinobacteria; o_Actinomycetales; **f_Gordoniaceae**		0.0023	5.7199	0.0078	8.8506	2.80E-4
p_Actinobacteria; c_Actinobacteria; o_Actinomycetales; f_Gordoniaceae; **g_Gordonia**		0.0119	5.4494	0.0144	8.8126	0.0014
p_Actinobacteria; c_Actinobacteria; o_Actinomycetales; f_Gordoniaceae; **g_Gordonia; s_paraffinivorans**		0.0267			7.7795	0.0292
p_Actinobacteria; c_Actinobacteria; o_Actinomycetales; f_Micrococcaceae; **g_Rothia; s_dentocariosa**					23.503	2.37E-16
p_Actinobacteria; c_Actinobacteria; o_Bifidobacteriales; f_Bifidobacteriaceae; **g_Bifidobacterium; s_adolescentis**		0.0436				
p_Bacteroidetes; **c_Bacteroidia**			2.7277	0.0437	1.8549	0.0493
p_Bacteroidetes; c_Bacteroidia; o_Bacteroidales; f_Porphyromonadaceae; **g_Barnesiella; s_intestinihominis**		0.0352				
p_Bacteroidetes; c_Bacteroidia; o_Bacteroidales; f_Porphyromonadaceae; **g_Butyricimonas; s_sp.**		0.0352				
p_Bacteroidetes; c_Bacteroidia; o_Bacteroidales; f_Prevotellaceae; **g_Prevotella; s_oris**		0.0307				
p_Bacteroidetes; c_Bacteroidia; o_Bacteroidales; f_Prevotellaceae; **g_Prevotella; s_timonensis**		0.0429				
p_Firmicutes; **c_Bacilli**			2.7377	0.0437		
p_Firmicutes; c_Bacilli; o_Bacillales; **f_Bacillales incertae sedis**		0.0109	6.5384	0.0078	9.8326	2.80E-4
p_Firmicutes; c_Bacilli; o_Bacillales; f_ Bacillales incertae sedis; **g_Gemella**			5.6679	0.0307	24.0	3.00E-18
p_Firmicutes; c_Bacilli; **o_Lactobacillales**			4.0028	0.0201		
p_Firmicutes; c_Bacilli; o_Lactobacillales; f_Lactobacillaceae; **g_Lactobacillus; s_kitasatonis**		0.0111			7.4707	0.0437
p_Firmicutes; c_Bacilli; o_Lactobacillales; **f_Streptococcaceae**			4.3043	0.0364		
p_Firmicutes; c_Clostridia; o_Clostridiales; f_Clostridiaceae; **g_Clostridium; s_asparagiforme**		0.0229				
p_Firmicutes; c_Clostridia; o_Clostridiales; f_Clostridiaceae; **g_Clostridium; s_aldenense**		0.0096	7.6431	0.0203	9.8939	0.0012
p_Firmicutes; c_Clostridia; o_Clostridiales; f_Peptostreptococcaceae; **g_ Peptostreptococcus; s_stomatis**		0.0352				
p_Firmicutes; c_Clostridia; o_Clostridiales; f_Eubacteriaceae; **g_Eubacterium; s_siraeum**		0.0447				
p_Firmicutes; c_Clostridia; o_Clostridiales; f_Ruminococcaceae; **g_Ruminococcus; s_callidus**		0.0084			24.045	3.28E-15
p_Firmicutes; c_Negativicutes; o_Selenomonadales; f_Acidaminococcaceae; **g_Acidaminococcus; s_intestini**		0.0084			6.4451	0.0255
p_Firmicutes; c_Negativicutes; o_Selenomonadales; f_Acidaminococcaceae; **g_Phascolarctobacterium**			4.7758	0.0414		
p_Firmicutes; c_Negativicutes; o_Selenomonadales; f_Veillonellaceae; **g_Dialister; s_invisus**		0.0398				
p_Firmicutes; c_Negativicutes; o_Selenomonadales; f_Veillonellaceae; **g_Dialister; s_pneumosintes**		0.0332				
p_Firmicutes; c_Negativicutes; o_Selenomonadales; f_Veillonellaceae; **g_Dialister; s_succinatiphilus**		0.0436			23.535	1.17E-14
p_Proteobacteria; c_Gammaproteobacteria; o_Pseudomonadales; f_Pseudomonadaceae; g_Pseudomonas**; s_aeruginosa**		0.0084			23.555	2.27E-14
**Higher abundance in UC patients diagnosed for Left sided colitis**						
p_Actinobacteria; c_Actinobacteria; o_Actinomycetales; **f_Microbacteriaceae**			-3.3816	0.0170	3.5263	0.0368
p_Bacteroidetes; **c_Cytophagia**		4.30E-4	-5.85	7.64E-5	-8.9345	2.70E-4
p_Bacteroidetes; c_Cytophagia; **o_Cytophagales**		0.0023	-5.6777	6.00E-5	-8.4613	3.40E-4
p_Bacteroidetes; c_Cytophagia; o_Cytophagales; **f_Cytophagaceae**		0.0023	-6.6716	1.40E-5	-8.4394	0.0025
p_Firmicutes; c_Clostridia; o_Clostridiales; f_Clostridiaceae; **g_Clostridium; s_bartlettii**			-4.9426	0.0276		
p_Firmicutes; c_Clostridia; o_Clostridiales; f_Lachnospiraceae; **g_Coprococcus; s_catus**			-4.4793	0.0203		
p_Firmicutes; c_Clostridia; o_Clostridiales; **f_Peptoniphilaceae**			-4.3669	0.0258		
p_Firmicutes; c_Clostridia; o_Clostridiales; f_Peptoniphilaceae; **g_Finegoldia**			-4.4005	0.0246		
p_Firmicutes; c_Clostridia; o_Clostridiales; f_Peptoniphilaceae; **g_Finegoldia; s_magna**			-5.1303	0.0203		
p_Firmicutes; c_Clostridia; o_Clostridiales; f_Ruminococcaceae; **g_Ruminococcus; s_gauvreauii**			-5.2798	0.0203		
p_Firmicutes; c_Clostridia; o_Clostridiales; f_Ruminococcaceae; **g_Ruminococcus; s_lactaris**			-3.477	0.0276		
p_Firmicutes; c_Bacilli; o_Bacillales; **f_ Bacillaceae**			-5.018	0.0093		
p_Firmicutes; c_Bacilli; o_Lactobacillales; f_Aerococcaceae; **g_Abiotrophia**			-4.5237	0.0048		
p_Firmicutes; c_Bacilli; o_Lactobacillales; f_Aerococcaceae; **g_Abiotrophia; s_defectiva**			-6.2263	4.33E-4		
**p_Proteobacteria**			-2.0967	0.0040	-2.4093	0.0010
p_Proteobacteria; c_Alphaproteobacteria; o_Rhizobiales; f_Methylobacteriaceae; **g_Methylobacterium; s_jeotgali**			-4.8744	0.0203		
p_Proteobacteria; c_Alphaproteobacteria; o_Rhodobacterales; f_Rhodobacteraceae; **g_Paracoccus**			-3.564	0.0329		
p_Proteobacteria; **c_Gammaproteobacteria**			-2.8219	0.0167	-3.0749	0.0046
p_Proteobacteria; c_Gammaproteobacteria; o_Enterobacteriales; **f_Enterobacteriaceae**			-3.7641	0.0127		
p_Proteobacteria; c_Gammaproteobacteria; o_Enterobacteriales; f_Enterobacteriaceae; **g_Cronobacter**			-6.0259	0.0142		
p_Proteobacteria; c_Gammaproteobacteria; o_Enterobacteriales; f_Enterobacteriaceae; **g_Escherichia**			-7.2668	0.0021		
p_Proteobacteria; c_Gammaproteobacteria; o_Pseudomonadales; f_Moraxellaceae; **g_Acinetobacter; s_calcoaceticus**			-6.1384	4.33E-4		

Supplementary Table S2.
Differential abundance analysis of stools and tissues biopsies microbiomes in patients responsive to the therapy compared to those not responding to the treatment.

	Stools				Tissues			
	R [23] *vs* NR [13]				R [22] *vs* NR [7]			
Taxonomy	EdgeR		DESeq2		EdgeR		DESeq2	
	log2FC	FDR	log2FC	FDR	log2FC	FDR	log2FC	FDR
**Higher abundance in the microbiome of patient Responding (R) to the therapy or lower abundance in the one Non-Responding (NR) to the treatment**								
p_Actinobacteria; c_Actinobacteria; o_Actinomycetales; f_Corynebacteriaceae; **g_Corynebacterium; s_sp.**							22.807	3.3E-16
p_Actinobacteria; c_Actinobacteria; o_Bifidobacteriales; f_Bifidobacteriaceae; **g_Bifidobacterium; s_adolescentis**	4.0397	0.0143						
p_Bacteroidetes; c_Bacteroidia; o_Bacteroidales; f_Bacteroidaceae; **g_Bacteroides; s_thetaiotaomicron**	4.7279	0.0054						
p_Bacteroidetes; c_Bacteroidia; o_Bacteroidales; f_Rikenellaceae; **g_Alistipes; s_shahii**	4.752	2.0E-4	25.939	3.2E-24				
p_Bacteroidetes; c_Bacteroidia; o_Bacteroidales; f_Rikenellaceae; **g_Alistipes; s_putredinis**			8.5144	0.0102				
p_Firmicutes; c_Clostridia; o_Clostridiales; **f_Christensenellaceae**	2.6485	0.0099	25.218	4.9E-23				
p_Firmicutes; c_Clostridia; o_Clostridiales; f_Lachnospiraceae; **g_Roseburia; s_hominis**	4.0806	0.0238					21.252	2.5E-14
p_Firmicutes; c_Clostridia; o_Clostridiales; **f_Peptococcaceae**	3.3514	0.0044						
p_Firmicutes; c_Bacilli; o_Lactobacillales; f_Lactobacillaceae; **g_Lactobacillus; s_rogosae**	5.244	0.0132						
p_Firmicutes; c_Negativicutes; o_Selenomonadales; **f_Acidaminococcaceae**	3.8196	0.0367						
p_Firmicutes; c_Negativicutes; o_Selenomonadales; f_Acidaminococcaceae; **g_Phascolarctobacterium**	4.0129	0.0490						
p_Firmicutes; c_Negativicutes; o_Selenomonadales; f_Acidaminococcaceae; **g_Phascolarctobacterium; s_faecium**	3.9861	0.0418						
p_Proteobacteria; **c_Deltaproteobacteria**	3.3815	0.0425	7.5784	0.0069				
p_Proteobacteria; c_Deltaproteobacteria; **o_Desulfovibrionales**	4.0701	7.2E-5	7.868	0.0039				
p_Proteobacteria; c_Deltaproteobacteria; o_Desulfovibrionales; **f_Desulfovibrionaceae**	5.6458	9.8E-4	6.8318	0.0264				
p_Proteobacteria; c_Deltaproteobacteria; o_Desulfovibrionales; f_Desulfovibrionaceae; **g_Bilophila**	4.6846	0.0026	7.1447	0.0462				
p_Proteobacteria; c_Deltaproteobacteria; o_Desulfovibrionales; f_Desulfovibrionaceae; **g_Bilophila; s_wadsworthia**	6.0591	0.0012	7.3433	0.0176				
p_Proteobacteria; c_Gammaproteobacteria; o_Aeromonadales; f_Aeromonadaceae; **g_Aeromonas**					5.5973	0.0391	23.132	1.8E-12
**Higher abundance in the microbiome of patient NON-Responding (NR) to the therapy or lower abundance in the one Responding (R) to the treatment**								
p_Actinobacteria; **c_Actinobacteria**	-5.5534	2.6E-7						
p_Actinobacteria; c_Actinobacteria; **o_Coriobacteriales**	-4.2614	7.0E-5						
p_Actinobacteria; c_Actinobacteria; o_Coriobacteriales; **f_Coriobacteriaceae**	-3.1492	0.0049						
p_Actinobacteria; c_Actinobacteria; o_Coriobacteriales; f_Coriobacteriaceae; **g_Collinsella**	-2.9428	0.0160						
p_Actinobacteria; c_Actinobacteria; o_Coriobacteriales; f_Coriobacteriaceae; **g_Collinsella; s_aerofaciens**	-3.1575	0.0121						
p_Bacteroidetes; c_Bacteroidia; o_Bacteroidales; f_Bacteroidaceae; **g_Bacteroides**					-2.6752	0.0269		
p_Bacteroidetes; c_Bacteroidia; o_Bacteroidales; f_Rikenellaceae; **g_Alistipes; s_putredinis**					-3.5742	0.0341		
p_Bacteroidetes; c_Flavobacteriia; o_Flavobacteriales; f_Flavobacteriaceae; **g_Chryseobacterium**					-6.744	1.2E-7		
p_Firmicutes; **c_Bacilli**	-5.1915	1.9E-4						
p_Firmicutes; c_Bacilli; **o_Bacillales**	-5.1629	7.0E-5						
p_Firmicutes; c_Bacilli; o_Bacillales; **f_Bacillaceae**	-4.8473	2.0E-4						
p_Firmicutes; c_Bacilli; **o_Lactobacillales**	-2.9663	0.0159						
p_Firmicutes; c_Bacilli; o_Lactobacillales; **f_Carnobacteriaceae**	-5.061	2.7E-4						
p_Firmicutes; c_Bacilli; o_Lactobacillales; f_Carnobacteriaceae**; g_Granulicatella**	-5.212	3.6E-4						
p_Firmicutes; c_Bacilli; o_Lactobacillales; f_Carnobacteriaceae**; g_Granulicatella; s_adiacens**	-4.0741	0.0015						
p_Firmicutes; c_Bacilli; o_Lactobacillales; **f_Enterococcaceae**	-5.8199	0.0012						
p_Firmicutes; c_Bacilli; o_Lactobacillales; f_Enterococcaceae; **g_Enterococcus**	-6.1881	0.0011						
p_Firmicutes; c_Bacilli; o_Lactobacillales; f_Enterococcaceae; **g_Enterococcus; s_faecium**	-4.8807	0.0050						
p_Firmicutes; c_Bacilli; o_Lactobacillales; f_Lactobacillaceae; **g_Lactobacillus; s_delbrueckii**					-4.9988	0.0236		
p_Firmicutes; c_Bacilli; o_Lactobacillales; **f_Streptococcaceae**	-4.8273	1.0E-4						
p_Firmicutes; c_Bacilli; o_Lactobacillales; f_Streptococcaceae; **g_Streptococcus**	-4.8432	3.0E-4						
p_Firmicutes; c_Bacilli; o_Lactobacillales; f_Streptococcaceae; **g_Streptococcus; s_australis**	-2.9227	0.0202						
p_Firmicutes; c_Bacilli; o_Lactobacillales; f_Streptococcaceae; **g_Streptococcus; s_salivarius**	-2,9159	0.0240						
p_Firmicutes; c_Bacilli; o_Lactobacillales; f_Streptococcaceae; **g_Streptococcus; s_sinensis**	-3.4666	0.0112						
p_Firmicutes; c_Clostridia; **o_Clostridiales**	-2.2523	0.0068						
p_Firmicutes; c_Clostridia; o_Clostridiales; **f_Clostridiaceae**	-2.0034	0.0367						
p_Firmicutes; c_Clostridia; o_Clostridiales; f_Clostridiaceae; **g_Clostridium**	-2.1827	0.0490						
p_Firmicutes; c_Clostridia; o_Clostridiales; f_Clostridiaceae; **g_Clostridium; s_disporicum**	-3.0195	0.0262						
p_Firmicutes; c_Clostridia; o_Clostridiales; f_Clostridiaceae; **g_Clostridium; s_perfringens**	-5.2572	6.0E-4						

p_Firmicutes; c_Clostridia; o_ Clostridiales; **f_Clostridiales Family XIII. Incertae Sedis**	-2.1997	0.0215			-3.8268	0.0064		
p_Firmicutes; c_Clostridia; o_ Clostridiales; f_Eubacteriaceae; **g_Eubacterium**	-3.2594	0.0026			-3.4502	0.0156		
p_Firmicutes; c_Clostridia; o_ Clostridiales; f_Eubacteriaceae; **g_Eubacterium; s_ventriosum**	-3.4518	0.0132						
p_Firmicutes; c_Clostridia; o_ Clostridiales; f_ Lachnospiraceae; **g_Blautia; s_luti**	-4.0085	9.7E-4						
p_Firmicutes; c_Clostridia; o_ Clostridiales; f_Lachnospiraceae; **g_Eubacterium; s_hadrum**	-3.6252	0.0120						
p_Firmicutes; c_Clostridia; o_ Clostridiales; f_Lachnospiraceae; **g_Eubacterium; s_rectale**	-4.1311	3.3E-4						
p_Firmicutes; c_Clostridia; o_ Clostridiales; f_Peptoniphilaceae; **g_Peptoniphilus**					-4.4409	0.0026		
p_Firmicutes; c_Clostridia; o_ Clostridiales; f_Ruminococcaceae; **g_Faecalibacterium**					-2.9776	0.0104		
p_Firmicutes; c_Clostridia; o_ Clostridiales; f_Ruminococcaceae; **g_Rumiclostridium**					-3.7759	0.0020		
p_Firmicutes; c_Negativicutes; o_Selenomonadales; f_Veillonellaceae; **g_Veillonella; s_alcalescens**	-4.0803	0.0035						
p_Proteobacteria; c_Alphaproteobacteria; **o_Caulobacterales**					-3.7538	3.5E-4		
p_Proteobacteria; c_Alphaproteobacteria; o_Caulobacterales; **f_Caulobacteraceae**					-3.5446	0.0054		
p_Proteobacteria; c_Alphaproteobacteria; o_Caulobacterales; f_Caulobacteraceae; **g_Brevundimonas**					-9.2416	2.2E-12		
p_Proteobacteria; c_Alphaproteobacteria; o_Rhodobacterales; f_ Rhodobacteraceae**; g_Paracoccus**					-5.7179	1.6E-5		
p_Proteobacteria; c_Alphaproteobacteria; o_Sphingomonadales; f_ Sphingomonadaceae; **g_Sphingomonas**					-3.124	0.0214		
p_Proteobacteria; c_Betaproteobacteria; o_Burkholderiales; **f_Comamonadaceae**	-5.3725	1.9E-5						
p_Proteobacteria; c_Betaproteobacteria; o_Burkholderiales; **f_Burkholderiaceae**	-2.5854	0.0405						
p_Proteobacteria; **c_Gammaproteobacteria**	-6.2804	7.6E-6					-4.4048	3.4E-4
p_Proteobacteria; c_Gammaproteobacteria; **o_Enterobacteriales**	-4.8162	7.2E-5					-4.0392	0.0041
p_Proteobacteria; c_Gammaproteobacteria; o_Enterobacteriales; **f_ Enterobacteriaceae**	-3.3098	0.0068						
p_Proteobacteria; c_Gammaproteobacteria; o_Enterobacteriales; f_ Enterobacteriaceae; **g_Escherichia**	-5.949	6.9E-5						
p_Proteobacteria; c_Gammaproteobacteria; o_Enterobacteriales; f_ Enterobacteriaceae; **g_Escherichia; s_coli**	-4.678	3.3E-4						
p_Proteobacteria; c_Gammaproteobacteria; o_Enterobacteriales; f_ Enterobacteriaceae; **g_Escherichia/Shigella**	-6.2108	3.0E-4						
p_Proteobacteria; c_Gammaproteobacteria; o_Enterobacteriales; f_ Enterobacteriaceae; **g_Escherichia/Shigella; s_coli/dysenteriae**	-9.1191	1.8E-8	-21.74	5.8E-20				
p_Proteobacteria; c_Gammaproteobacteria; o_Enterobacteriales; f_ Enterobacteriaceae;**g_Trabulsiella**	-4.2717	3.0E-4						
p_Proteobacteria; c_Gammaproteobacteria; o_Pseudomonadales; **f_Moraxellaceae**					-3.4088	0.0090		
p_Proteobacteria; c_Gammaproteobacteria; o_Pseudomonadales; f_Moraxellaceae; **g_Acinetobacter**					-3.5597	0.0109		

We assessed the relative abundances of microorganisms in a group of patients with Ulcerative Colitis (UC). Data analyses were conducted on the microbiome of stool samples (shown in the middle columns) and tissue biopsies (located four columns to the left), focusing on patients undergoing therapy with anti-TNF alpha. We compared two groups: Responders (R), who exhibited an endoscopic Mayo score of 1 or less, and Non-Responders (NR) with a Mayo score >=2. The numbers in square brackets indicate the number of patients in each group. The complete taxonomy for each microorganism is provided in the left column, categorized as: (p_Phylum; c_Class; o_Order; f_Family; g_Genus; s_Species). The relative abundance analysis was computed using the EdgeR or DESeq2 statistical methods. The False Discovery Rate (FDR) represents the adjusted p-values for statistical significance for multiple comparisons. The fold-change (log2FC) indicates how much the abundance of each microorganism increases or decreases when comparing the two sample groups. FDR values of 0.05 or lower were considered statistically significant.

Supplementary Table S3a

		Stools							
		R (Mayo-0) *vs* pre (Mayo-3)		R (Mayo-1) *vs* pre (Mayo-3)		NR (Mayo-2) *vs* pre (Mayo-3)		NR (Mayo-3) *vs* pre (Mayo-3)	
	Genera	log2FC	FDR	log2FC	FDR	log2FC	FDR	log2FC	FDR
B	*Bacteroides*	-	-	-	-	2,9107	0.04054		
A	*Bifidobacterium*	-	-	-	-	2,6971	0.04054		
P	*Bilophila*	-	-	3,7726	0.0022599	-	-		
A	*Collinsella*	-	-	-	-	3,8298	0.0075296		
F	*Eubacterium*	-	-	2,0671	0.04201	-	-		
F	*Gemmiger*	3,3976	0.0030802	-	-	-	-		
P	*Haemophilus*	-	-	-	-	3,2701	0.04054		
F	*Lachnoclostridium*	-	-	2,2461	0.044724	-	-		
F	*Odoribacter*	3,3675	0.0034423	-	-	3,9837	0.0075296		
F	*Phascolarctobacterium*	5,6520	2,19E-04	4,0465	0.0088757	-	-		
F	*Blautia*	-2,4355	0.044191	-	-	-	-		
F	*Clostridium*	-3,2181	0.01125	-	-	-	-		
A	*Corynebacterium*	-7,2632	5,04E-06	-5,9628	2,69E-04	-5,1216	0.018597	**No differences**	
P	*Cronobacter*	-7,2632	1,63E-04	-5,9628	0.016036	-	-		
F	*Enterococcus*	-8,3443	0.0030802	-10,501	0.001734	-8,9041	0.04054		
P	*Escherichia*	-7,2632	2,19E-04	-5,9628	0.0080096	-	-		
P	*Escherichia_Shigella*	-7,2632	2,44E-05	-5,9628	0.011394	-	-		
F	*Eubacterium*	-4,2170	0.0030802	-	-	-	-		
F	*Lactobacillus*	-4,5113	0.012037	-	-	-	-		
F	*Oscillibacter*	-	-	-3,8387	0.011394	3,3147	0.016204		
A	*Propionibacterium*	-	-	-4,3837	0.0078434	-	-		
F	*Ruminococcus*	-2,4988	0.046877	4,5814	0.0022599	-	-		
F	*Staphylococcus*	-2,6885	0.039587	-5.245	0.001734	-4,8430	0.04054		
F	*Streptococcus*	-4,3661	0.01125	-4,0025	0.028533	-	-		
P	*Trabulsiella*	-7,2632	2,19E-04	-5,9628	0.035436	-	-		
F	*Veillonella*	-5,9134	0.0030802	-	-	-	-		

Supplementary Table S3b

	Tissues								
		**R (Mayo-0) *vs* pre (Mayo-3)**		**R (Mayo-1) *vs* pre (Mayo-3)**		**NR (Mayo-2) *vs* pre (Mayo-3)**		**NR (Mayo-3) *vs* pre (Mayo-3)**	
	**Genera**	**log2FC**	**FDR**	**log2FC**	**FDR**	**log2FC**	**FDR**	**log2FC**	**FDR**
**P**	*Aeromonas*	4,4123	0.0081564	4,3978	0.0066333	-	-	-	-
**B**	*Chryseobacterium*	-	-	-	-	4,7627	0.0043772	-	-
**A**	*Eggerthella*	2,6566	0.022654			3,0680	0.040866	-	-
**F**	*Eubacterium*	-	-	-	-	-	-	5,2877	0.0067158
**P**	*Paracoccus*	-	-	-	-	4,8768	0.0063263	-	-
**P**	*Salmonella*	-	-	3,8308	0.034741	-	-	5,2507	0.013282
**P**	*Serratia*	-	-	-	-	-	-	4,3904	0.047236
**P**	*Sphingomonas*	-	-	-	-	4,2390	0.010775	-	-
**P**	*Trabulsiella*	3,1805	0.044277	3,4794	0.041729	-	-	4,3678	0.047236
**F**	*Tyzzerella*	-	-	7,4267	4,85E-08	-	-	-	-
**P**	*Acinetobacter*	-3,6007	0.022654	-	-	-	-	-	-
**F**	*Anaerococcus*	-3,9023	0.022654	-	-	-	-	-	-
**B**	*Butyricimonas*	-	-	-3,8826	0.033113	-	-	-	-
**B**	*Cloacibacterium*	-3,4033	0.022654	-4,0501	0.034741	-	-	-	-
**F**	*Finegoldia*	-4,4509	0.01781	-	-	-	-	-	-
**P**	*Moraxella*	-4,5753	0.01781	-4,3711	0.044647	5,7756	5,38E-04	-	-
**F**	*Peptoniphilus*	-5,5280	0.0081564	-	-	-	-	-	-
**F**	*Streptococcus*	-4,0039	0.0081564	-3,2286	0.044647	-	-	-	-

Supplementary Table S4: Supervised Random Forest Analysis

Stools		MDA	R	NR	pre			Tissues		MDA	R	NR	pre		
Bacteroidia	B	0.0189	++	+	-			***Gammaproteobacteria***	P	0.0213	++	+	-		
Bacteroidales	B	0.0169	++	+	-			***Enterobacteriales***	P	0.0129	++	+	-		
Bacteroidetes	B	0.0155	++	+	-			***Bacilli***	F	0.0117	++	-	+		
Alphaproteobacteria	P	0.0111	++	+	-			***Bacillales***	F	0.0078	++	-	+	42%	F
Rhizobiales	P	0.0105	++	+	-	50%	F	***Staphylococcus***	F	0.0072	++	-	+	0%	B
Phascolarctobacterium	F	0.0067	++	+	-	25%	B	***Trabulsiella***	P	0.0071	++	+	-	50%	P
Bifidobacterium adolescentis	A	0.0058	++	+	-	17%	P	***Actinomycetales***	A	0.0067	++	-	+	8%	A
Negativicutes	F	0.0055	++	-	+	8%	A	***Staphylococcaceae***	F	0.0060	++	-	+		
Phascolarctobacterium faecium	F	0.0051	++	+	-			***Enterobacteriaceae***	P	0.0050	++	+	-		
Gemmiger	F	0.0042	++	+	-			***Trabulsiella odontotermitis***	P	0.0045	++	+	-		
Clostridium sp.	F	0.0033	++	+	-			***Staphylococcus hominis***	F	0.0041	++	+	-		
Erysipelotrichia	F	0.0027	++	+	-			***Escherichia coli***	P	0.0024	++	+	-		
**Stools**		**MDA**	**R**	**NR**	**pre**			**Tissues**		**MDA**	**R**	**NR**	**pre**		
Pasteurellales	P	0.0134	-	++	+			***Proteobacteria***	P	0.0330	+	++	-		
Haemophilus	P	0.0060	-	++	+	30%	F	***Deltaproteobacteria***	P	0.0129	+	++	-	0%	F
Veillonella	F	0.0056	-	++	+	10%	B	***Desulfovibrionales***	P	0.0077	+	++	-	17%	B
Bacteroidaceae	B	0.0054	+	++	-	60%	P	***Escherichia***	P	0.0038	+	++	-	67%	P
Lachnospiraceae	F	0.0053	-	++	+	0%	A	***Bacteroides ovatus***	B	0.0037	+	++	-	17%	A
Pasteurellaceae	P	0.0052	-	++	+			***Eggerthella***	A	0.0036	+	++	-		
Escherichia	P	0.0045	-	++	+										
Haemophilus parainfluenzae	P	0.0038	-	++	+										
Escherichia coli	P	0.0035	-	++	+										
Streptococcus salivarius	F	0.0033	-	++	+										
**Stools**		**MDA**	**R**	**NR**	**pre**			**Tissues**		**MDA**	**R**	**NR**	**pre**		
Actimycetales	A	0.0165	-	+	++			***Clostriales Family XI Incertae sedis***	F	0.0137	-	+	++	40%	F
Corynebacterium	A	0.0101	-	+	++	50%	F	***Corynebacteriaceae***	A	0.0038	+	-	++	0%	B
Corynebacteriaceae	A	0.0098	-	+	++	0%	B	***Corynebacterium***	A	0.0037	+	-	++	20%	P
Staphyloccaceae	F	0.0064	-	+	++	0%	P	***Finegoldia magna***	F	0.0025	-	+	++	40%	A
Dorea	F	0.0058	-	+	++	50%	A	***Escherichia/Shigella coli/dysenteriae***	P	0.0018	+	-	++		
Propionibacterium acnes	F	0.0040	-	+	++										

Supplementary Table S5: Inferred metabolic pathways from stool-sampled microbiomes

KO	logFC R3 vs pFu	p Value	
K11178	-2,2336	2.85558785185537e-05	K11178 yagS; xanthine dehydrogenase YagS FAD-binding subunit [EC:1.17.1.4]
K01220	-4,7665	3.15862223491692e-05	K01220 E3.2.1.85, lacG; 6-phospho-beta-galactosidase [EC:3.2.1.85]
K01218	2,0957	3.94716407415666e-05	K01218 gmuG; mannan endo-1,4-beta-mannosidase [EC:3.2.1.78]
K07749	-3,5483	4.23586475355163e-05	K07749 frc; formyl-CoA transferase [EC:2.8.3.16]
K02530	-4,3345	4.58904206998095e-05	K02530 lacR; DeoR family transcriptional regulator, lactose phosphotransferase system repressor
K03693	-4,6414	4.71589053643073e-05	K03693 pbp1b; penicillin-binding protein 1B
K01635	-4,4864	4.94765277776234e-05	K01635 lacD; tagatose 1,6-diphosphate aldolase [EC:4.1.2.40]
K02788	-4,3485	5.57977586724353e-05	K02788 lacE; lactose PTS system EIICB component [EC:2.7.1.207]
K01819	-4,5277	5.58174905960363e-05	K01819 E5.3.1.26, lacA, lacB; galactose-6-phosphate isomerase [EC:5.3.1.26]
K06878	-3,2784	5.65896282541726e-05	K06878 K06878; tRNA-binding protein
K18702	-2,8193	5.71775737649411e-05	K18702 uctC; CoA:oxalate CoA-transferase [EC:2.8.3.19]
K10039	-4,1394	5.82548791412202e-05	K10039 peb1A, glnH; aspartate/glutamate/glutamine transport system substrate-binding protein
K02781	-2,7878	6.0331828158756e-05	K02781 srlB; glucitol/sorbitol PTS system EIIA component [EC:2.7.1.198]
K04844	-1,6993	6.08680832255255e-05	K04844
K07707	-3,2841	7.04077157033657e-05	K07707 agrA, blpR, fsrA; two-component system, LytTR family, response regulator AgrA
K09134	-3,5373	7.15571438085895e-05	K09134 flA; adenosyl-fluoride synthase [EC:2.5.1.63]
K16169	-3,7880	7.46120722618415e-05	K16169 pbuX; xanthine permease
K06994	-3,8886	7.46148134915982e-05	K06994 K06994; putative drug exporter of the RND superfamily
K03367	-3,3995	7.58002322989639e-05	K03367 dltA; D-alanine--poly(phosphoribitol) ligase subunit 1 [EC:6.1.1.13]
K03739	-3,5025	7.61378874357962e-05	K03739 dltB; membrane protein involved in D-alanine export
K02761	-2,9160	7.94369229453129e-05	K02761 celB, chbC; cellobiose PTS system EIIC component
K08996	-3,5400	8.31002025809323e-05	K08996 yagU; putative membrane protein
K03740	-3,3672	8.45926551917776e-05	K03740 dltD; D-alanine transfer protein
K10040	-3,8495	9.00452329838967e-05	K10040 peb1B, glnP, glnM; aspartate/glutamate/glutamine transport system permease protein
K03697	-4,3917	9.32495159477865e-05	K03697 clpE; ATP-dependent Clp protease ATP-binding subunit ClpE

K14188	-3,5641	9.43878977741904e-05	K14188 dltC; D-alanine--poly(phosphoribitol) ligase subunit 2 [EC:6.1.1.13]
K16013	-2,9438	9.55734796615446e-05	K16013 cydD; ATP-binding cassette, subfamily C, bacterial CydD
K16012	-3,0676	9.57762760779194e-05	K16012 cydC; ATP-binding cassette, subfamily C, bacterial CydC
K18892	-4,4342	9.5911826496322e-05	K18892 patB, rscB, lmrC, satB; ATP-binding cassette, subfamily B, multidrug efflux pump
K18891	-4,4342	9.59445948466014e-05	K18891 patA, rscA, lmrC, satA; ATP-binding cassette, subfamily B, multidrug efflux pump
K10041	-3,8743	9.93230853149002e-05	K10041 peb1C, glnQ; aspartate/glutamate/glutamine transport system ATP-binding protein [EC:7.4.2.1]
K02246	-4,0867	0.00010291252860587	K02246 comGD; competence protein ComGD
K07305	-2,8470	0.000103408572201799	K07305 msrB; peptide-methionine (R)-S-oxide reductase [EC:1.8.4.12]
K08368	-3,2994	0.000110168607247509	K08368 yaaU; MFS transporter, putative metabolite transport protein
K03549	-2,6943	0.000111796982758944	K03549 kup; KUP system potassium uptake protein
K02786	-4,3991	0.000115222495188621	K02786 lacF; lactose PTS system EIIA component [EC:2.7.1.207]
K08987	-3,9744	0.000119286220956679	K08987 K08987; putative membrane protein
K01577	-3,7001	0.000121955707730296	K01577 oxc; oxalyl-CoA decarboxylase [EC:4.1.1.8]
K00158	-4,4191	0.000124851808186255	K00158 spxB, poxL; pyruvate oxidase [EC:1.2.3.3]
K02082	-2,7398	0.000128418840891915	K02082 agaS; D-galactosamine 6-phosphate deaminase/isomerase [EC:3.5.99.-]
K03647	-2,8654	0.000129690044527226	K03647 nrdI; protein involved in ribonucleotide reduction
K00383	-2,8761	0.000132505020746739	K00383 GSR, gor; glutathione reductase (NADPH) [EC:1.8.1.7]
K02779	-2,5941	0.000133720177893231	K02779 ptsG; glucose PTS system EIICB or EIICBA component [EC:2.7.1.199]
K02773	-2,6208	0.000134955542767789	K02773 gatA, sgcA; galactitol PTS system EIIA component [EC:2.7.1.200]
K00938	-4,0157	0.000138534006792382	K00938 E2.7.4.2, mvaK2; phosphomevalonate kinase [EC:2.7.4.2]
K06198	-4,0465	0.000139754541928787	K06198 coiA; competence protein CoiA
K12555	-4,0077	0.000140320738572319	K12555 pbp2A; penicillin-binding protein 2A [EC:2.4.99.28 3.4.16.4]
K01322	1,7790	0.000140468128722093	K01322 PREP; prolyl oligopeptidase [EC:3.4.21.26]
K14205	-3,1528	0.000141882354274467	K14205 mprF, fmtC; phosphatidylglycerol lysyltransferase [EC:2.3.2.3]
K00869	-4,0058	0.000142686553157207	K00869 MVK, mvaK1; mevalonate kinase [EC:2.7.1.36]
K01641	-4,0142	0.000143463291557344	K01641 HMGCS; hydroxymethylglutaryl-CoA synthase [EC:2.3.3.10]
K01597	-3,9995	0.000144632524423033	K01597 MVD, mvaD; diphosphomevalonate decarboxylase [EC:4.1.1.33]
K02236	-3,7587	0.000146723651735402	K02236 comC; leader peptidase (prepilin peptidase) / N-methyltransferase [EC:3.4.23.43 2.1.1.-]
K02244	-4,0004	0.000147814602082503	K02244 comGB; competence protein ComGB
K03095	-4,0164	0.00015306844472977	K03095 sprL; SprT-like protein

K02042	-3,2410	0.000155472823535569	K02042 phnE; phosphonate transport system permease protein
K07341	-3,2465	0.000159124457537198	K07341 doc; death on curing protein
K01215	-3,3099	0.000162261735521893	K01215 dexB; glucan 1,6-alpha-glucosidase [EC:3.2.1.70]
K02242	-3,3080	0.000164418444624376	K02242 comFC; competence protein ComFC
K16213	2,0262	0.000168414684340123	K16213 cbe, mbe; cellobiose epimerase [EC:5.1.3.11]
K07029	-3,1387	0.000170755782550277	K07029 dagK; diacylglycerol kinase (ATP) [EC:2.7.1.107]
K02248	-4,2007	0.000173684762304742	K02248 comGF; competence protein ComGF
K00867	-2,8932	0.000178921768288006	K00867 coaA; type I pantothenate kinase [EC:2.7.1.33]
K01916	-2,7470	0.000182500299834837	K01916 nadE; NAD+ synthase [EC:6.3.1.5]
K02796	-2,4939	0.000183996236748607	K02796 manZ; mannose PTS system EIID component
K01226	-2,3664	0.000184691018486804	K01226 treC; trehalose-6-phosphate hydrolase [EC:3.2.1.93]
K03492	-3,0035	0.000194521983100189	K03492 gmuR; GntR family transcriptional regulator, regulator of glucomannan utilization
K01838	-2,4184	0.000194759819723638	K01838 pgmB; beta-phosphoglucomutase [EC:5.4.2.6]
K07570	-3,2514	0.000204586396731012	K07570 GSP13; general stress protein 13
K00364	-2,6038	0.000205485942388634	K00364 guaC, GMPR; GMP reductase [EC:1.7.1.7]
K01223	-2,2161	0.000209548705642082	K01223 E3.2.1.86B, bglA; 6-phospho-beta-glucosidase [EC:3.2.1.86]
K10985	-3,4341	0.00021648196247859	K10985 agaC; galactosamine PTS system EIIC component
K00054	-3,7258	0.000219963519732159	K00054 mvaA; hydroxymethylglutaryl-CoA reductase [EC:1.1.1.88]
K00564	-2,5226	0.000223619435010433	K00564 rsmC; 16S rRNA (guanine1207-N2)-methyltransferase [EC:2.1.1.172]
K02795	-2,4427	0.000223961998266974	K02795 manY; mannose PTS system EIIC component
K07741	-3,7661	0.000232436123935792	K07741 antB; anti-repressor protein
K06286	-3,6186	0.000247759973305477	K06286 ezrA; septation ring formation regulator
K06726	-2,1252	0.000251888086533524	K06726 rbsD; D-ribose pyranase [EC:5.4.99.62]
K02794	-2,3472	0.00025362661790042	K02794 manX; mannose PTS system EIIAB component [EC:2.7.1.191]
K02245	-3,5190	0.000262446119705228	K02245 comGC; competence protein ComGC
K07177	-3,2479	0.000274298925933553	K07177 K07177; Lon-like protease
K02044	-3,1605	0.000284355292972326	K02044 phnD; phosphonate transport system substrate-binding protein
K03346	-3,1919	0.00028661966571633	K03346 dnaB; replication initiation and membrane attachment protein
K01727	-2,6337	0.000290555130129085	K01727 hysA, hylA, hylB; hyaluronate lyase [EC:4.2.2.1]
K19005	-3,7325	0.000295339434370287	K19005 ltaS; lipoteichoic acid synthase [EC:2.7.8.20]

K03293	-2,9609	0.00029739926329624	K03293 TC.AAT; amino acid transporter, AAT family
K02086	-3,1679	0.000301345913548078	K02086 dnaD; DNA replication protein
K02243	-3,1808	0.000302403182558298	K02243 comGA; competence protein ComGA
K18692	-3,1711	0.000303384314778728	K18692 cshB; ATP-dependent RNA helicase CshB [EC:5.6.2.7]
K11144	-3,1549	0.000304211752999548	K11144 dnaI; primosomal protein DnaI
K03048	-3,0304	0.000307260614867483	K03048 rpoE; DNA-directed RNA polymerase subunit delta
K02240	-3,1598	0.000308833279147823	K02240 comFA; competence protein ComFA
K06948	-3,1285	0.000319781178908152	K06948 yqeH; 30S ribosome assembly GTPase
K09976	-3,1438	0.00032140383948736	K09976 K09976; uncharacterized protein
K07706	-2,8728	0.00032344714479713	K07706 agrC, blpH, fsrC; two-component system, LytTR family, sensor histidine kinase AgrC [EC:2.7.13.3]
K16509	-3,0070	0.00033900246409675	K16509 spxA; regulatory protein spx
K06956	-2,0942	0.000339816220467067	K06956 K06956; uncharacterized protein
K03476	-1,4815	0.000340105374430839	K03476 ulaG; L-ascorbate 6-phosphate lactonase [EC:3.1.1.-]
K12700	-3,1673	0.000341665904182037	K12700 rihC; non-specific ribonucleoside hydrolase [EC:3.2.2.-]
K00851	-2,3662	0.000349479733290086	K00851 idnK, gntK; gluconokinase [EC:2.7.1.12]
K08659	-3,4318	0.000349586966783787	K08659 pepDA, pepDB; dipeptidase [EC:3.4.-.-]
K07455	-1,7848	0.000353069138866657	K07455 recT; recombination protein RecT
K02798	-2,0102	0.000358835967053493	K02798 cmtB; mannitol PTS system EIIA component [EC:2.7.1.197]
K02859	-4,4531	0.000364080915010849	K02859 ribT; riboflavin biosynthesis RibT protein
K09384	-3,0423	0.000369380827068322	K09384 K09384; uncharacterized protein
K10986	-2,9358	0.000369758140668389	K10986 agaD; galactosamine PTS system EIID component
K02760	-2,0419	0.000383082807988164	K02760 celA, chbB; cellobiose PTS system EIIB component [EC:2.7.1.196 2.7.1.205]
K03316	-2,5047	0.000384238171241606	K03316 nhaP, nhaS1, nhaS2; Na+:H+ antiporter
K13678	-3,7174	0.000388023095310801	K13678 cpoA; 1,2-diacylglycerol-3-alpha-glucose alpha-1,2-galactosyltransferase [EC:2.4.1.-]
K02774	-2,1298	0.00039635047379131	K02774 gatB, sgcB; galactitol PTS system EIIB component [EC:2.7.1.200]
K15524	-1,6679	0.000396957581779942	K15524 mngB; 2-O-(6-phospho-alpha-D-mannosyl)-D-glycerate hydrolase [EC:3.2.1.-]
K02041	-2,8273	0.000413417213348537	K02041 phnC; phosphonate transport system ATP-binding protein [EC:7.3.2.2]
K01101	-3,3736	0.000435823665756476	K01101 E3.1.3.41; 4-nitrophenyl phosphatase [EC:3.1.3.41]
K03486	-2,5720	0.000440940607016533	K03486 treR2, treR; GntR family transcriptional regulator, trehalose operon transcriptional repressor
K02783	-1,7075	0.000448211777672031	K02783 srlA; glucitol/sorbitol PTS system EIIC component

K13256	-2,4006	0.000456297257338417	K13256 psiE; protein PsiE
K02077	-3,0382	0.000482871626205709	K02077 ABC.ZM.S; zinc/manganese transport system substrate-binding protein
K01281	-3,4547	0.000497553642838355	K01281 pepX; X-Pro dipeptidyl-peptidase [EC:3.4.14.11]
K01823	-2,6288	0.000502478713195416	K01823 idi, IDI; isopentenyl-diphosphate Delta-isomerase [EC:5.3.3.2]
K00135	-2,7061	0.000510795679804043	K00135 gabD; succinate-semialdehyde dehydrogenase / glutarate-semialdehyde dehydrogenase [EC:1.2.1.16 1.2.1.79 1.2.1.20]
K09963	-2,7595	0.000545243987474623	K09963 K09963; uncharacterized protein
K16924	-3,2058	0.000553978003944271	K16924 mtsT; energy-coupling factor transport system substrate-specific component
K03817	-2,7844	0.000562672004102988	K03817 rimL; ribosomal-protein-serine acetyltransferase [EC:2.3.1.-]
K18104	-4,2025	0.000574956840426765	K18104 abcA, bmrA; ATP-binding cassette, subfamily B, bacterial AbcA/BmrA [EC:7.6.2.2]
K03713	-2,8650	0.000575489041899833	K03713 glnR; MerR family transcriptional regulator, glutamine synthetase repressor
K09685	-2,7746	0.000584519783851601	K09685 purR; HTH-type transcriptional regulator, purine operon repressor
K02075	-2,8854	0.000584646088460429	K02075 ABC.ZM.P; zinc/manganese transport system permease protein
K03724	-2,0942	0.00059322645220256	K03724 lhr; ATP-dependent helicase Lhr and Lhr-like helicase [EC:5.6.2.6 5.6.2.4]
K00574	-1,9757	0.000610815021924145	K00574 cfa; cyclopropane-fatty-acyl-phospholipid synthase [EC:2.1.1.79]
K01595	-2,2757	0.000611184977449262	K01595 ppc; phosphoenolpyruvate carboxylase [EC:4.1.1.31]
K01222	-1,3274	0.000618736038104831	K01222 E3.2.1.86A, celF; 6-phospho-beta-glucosidase [EC:3.2.1.86]
K06193	-1,6742	0.000620584149027746	K06193 phnA; protein PhnA
K02053	-1,4933	0.00063305062780258	K02053 ABC.SP.P; putative spermidine/putrescine transport system permease protein
K02759	-1,7876	0.000672102125960814	K02759 celC, chbA; cellobiose PTS system EIIA component [EC:2.7.1.196 2.7.1.205]
K07006	-3,2196	0.000693682306562366	K07006 K07006; uncharacterized protein
K02074	-2,8620	0.00071498766863747	K02074 ABC.ZM.A; zinc/manganese transport system ATP-binding protein
K00068	-1,5925	0.000728834106454136	K00068 srlD; sorbitol-6-phosphate 2-dehydrogenase [EC:1.1.1.140]
K01304	-2,2619	0.00074110988483863	K01304 pcp; pyroglutamyl-peptidase [EC:3.4.19.3]
K07038	-2,1012	0.000795013917674135	K07038 K07038; inner membrane protein
K00841	-3,0424	0.00079717751488463	K00841 patA; aminotransferase [EC:2.6.1.-]
K01674	-2,4279	0.000802830905022403	K01674 cah; carbonic anhydrase [EC:4.2.1.1]
K01488	-1,7445	0.00082075913132135	K01488 add, ADA; adenosine deaminase [EC:3.5.4.4]
K08724	-3,1230	0.000825178768923078	K08724 pbpB; penicillin-binding protein 2B
K02055	-1,4786	0.000828416582246877	K02055 ABC.SP.S; putative spermidine/putrescine transport system substrate-binding protein
K04761	0,9838	0.000833408818805564	K04761 oxyR; LysR family transcriptional regulator, hydrogen peroxide-inducible genes activator

K01261	-2,8265	0.000846901054641254	K01261 pepA; glutamyl aminopeptidase [EC:3.4.11.7]
K16925	-2,8387	0.000871689243974498	K16925 ykoE; energy-coupling factor transport system permease protein
K02819	-1,5055	0.000878547770936065	K02819 treB, treP; trehalose PTS system EIIBC or EIIBCA component [EC:2.7.1.201]
K01426	-1,4951	0.000901216790950184	K01426 E3.5.1.4, amiE; amidase [EC:3.5.1.4]
K07457	-3,1746	0.000914380780660119	K07457 K07457; endonuclease III related protein
K01575	-2,6242	0.000919937510443133	K01575 alsD, budA, aldC; acetolactate decarboxylase [EC:4.1.1.5]
K05847	-1,8788	0.000940417409141139	K05847 opuA; osmoprotectant transport system ATP-binding protein [EC:7.6.2.9]
K02054	-1,5707	0.00094957708399054	K02054 ABC.SP.P1; putative spermidine/putrescine transport system permease protein
K01239	-2,3310	0.000973793446528352	K01239 E3.2.2.1; purine nucleosidase [EC:3.2.2.1]
K08161	-2,3680	0.000986924003066844	K08161 mdtG, pmrA; MFS transporter, DHA1 family, multidrug resistance protein
K01071	-2,8854	0.000991484573372355	K01071 MCH; medium-chain acyl-[acyl-carrier-protein] hydrolase [EC:3.1.2.21]
K06142	1,2293	0.000998217538335761	K06142 hlpA, ompH; outer membrane protein
K19286	1,5126	0.00101110483257016	K19286 nfrA2; FMN reductase [NAD(P)H] [EC:1.5.1.39]
K09925	-1,3867	0.00103470627360457	K09925 K09925; uncharacterized protein
K05311	-2,5486	0.00108991280205775	K05311 cggR; central glycolytic genes regulator
K03667	-2,2259	0.00110637534028715	K03667 hslU; ATP-dependent HslUV protease ATP-binding subunit HslU
K01821	-2,2032	0.00111795096102565	K01821 praC, xylH; 4-oxalocrotonate tautomerase [EC:5.3.2.6]
K01419	-2,2229	0.00112134152556501	K01419 hslV, clpQ; ATP-dependent HslUV protease, peptidase subunit HslV [EC:3.4.25.2]
K11041	-2,0328	0.00119420407517516	K11041 eta; exfoliative toxin A/B
K19157	-2,3098	0.00119457294565589	K19157 yafQ; mRNA interferase YafQ [EC:3.1.-.-]
K02348	-1,5487	0.00119523892534266	K02348 elaA; ElaA protein
K05846	-1,6883	0.0012030297244381	K05846 opuBD; osmoprotectant transport system permease protein
K03704	-1,0654	0.00120550385410443	K03704 cspA; cold shock protein
K02549	-1,7126	0.00126498807529923	K02549 menC; o-succinylbenzoate synthase [EC:4.2.1.113]
K02810	-1,7900	0.00128541827378796	K02810 scrA, sacP, sacX, ptsS; sucrose PTS system EIIBCA or EIIBC component [EC:2.7.1.211]
K07146	-2,0457	0.00131725779482661	K07146 K07146; UPF0176 protein
K06867	-1,5776	0.00132244261780757	K06867 K06867; uncharacterized protein
K03773	1,3510	0.00132440103071801	K03773 fklB; FKBP-type peptidyl-prolyl cis-trans isomerase FklB [EC:5.2.1.8]
K01271	-1,8233	0.00133832401361449	K01271 pepQ; Xaa-Pro dipeptidase [EC:3.4.13.9]
K17195	-1,8721	0.00134945830586607	K17195 alsE; D-allulose-6-phosphate 3-epimerase [EC:5.1.3.-]

K09773	-1,9419	0.00137895288199866	K09773 ppsR; [pyruvate, water dikinase]-phosphate phosphotransferase / [pyruvate, water dikinase] kinase [EC:2.7.4.28 2.7.11.33]
K02552	-1,6968	0.00138619109883597	K02552 menF; menaquinone-specific isochorismate synthase [EC:5.4.4.2]
K18471	-1,3420	0.00140593781774257	K18471 ydjG; methylglyoxal reductase [EC:1.1.1.-]
K09788	-1,7020	0.00143695998278246	K09788 prpF; 2-methylaconitate isomerase [EC:5.3.3.-]
K18221	2,2770	0.00150523536058127	K18221 tetX; tetracycline 11a-monooxygenase, tetracycline resistance protein [EC:1.14.13.231]
K02052	-1,3346	0.00151324401940748	K02052 ABC.SP.A; putative spermidine/putrescine transport system ATP-binding protein
K02221	-1,7523	0.00162087606346268	K02221 yggT; YggT family protein
K11050	-2,4332	0.00165224783101452	K11050 cylA; multidrug/hemolysin transport system ATP-binding protein
K01259	-2,4887	0.00166386921526604	K01259 pip; proline iminopeptidase [EC:3.4.11.5]
K11051	-2,4368	0.00167307477224762	K11051 cylB; multidrug/hemolysin transport system permease protein
K01750	-1,6041	0.00171554706064904	K01750 ocd; ornithine cyclodeaminase [EC:4.3.1.12]
K19267	-1,8275	0.00172717616851589	K19267 qorB; NAD(P)H dehydrogenase (quinone) [EC:1.6.5.2]
K00351	1,2076	0.00174945075489286	K00351 nqrF; Na+-transporting NADH:ubiquinone oxidoreductase subunit F [EC:7.2.1.1]
K03652	-2,4275	0.00175529271493961	K03652 MPG; DNA-3-methyladenine glycosylase [EC:3.2.2.21]
K12111	-1,3964	0.00176049420638061	K12111 ebgA; evolved beta-galactosidase subunit alpha [EC:3.2.1.23]
K03771	1,1334	0.00176188059985743	K03771 surA; peptidyl-prolyl cis-trans isomerase SurA [EC:5.2.1.8]
K02440	-1,8452	0.00176444504775376	K02440 glpF; glycerol uptake facilitator
K02777	-1,6148	0.00176460774816795	K02777 crr; sugar PTS system EIIA component [EC:2.7.1.-]
K02793	-2,1702	0.00177321233875412	K02793 manXa; mannose PTS system EIIA component [EC:2.7.1.191]
K05710	-1,9228	0.00177535761203636	K05710 hcaC; 3-phenylpropionate/trans-cinnamate dioxygenase ferredoxin component
K00347	1,2630	0.00181904109531654	K00347 nqrB; Na+-transporting NADH:ubiquinone oxidoreductase subunit B [EC:7.2.1.1]
K07263	1,1722	0.00182247600932509	K07263 pqqL; zinc protease [EC:3.4.24.-]
K09698	-2,3280	0.00182625241237742	K09698 gltX; nondiscriminating glutamyl-tRNA synthetase [EC:6.1.1.24]
K04084	1,0359	0.00183298173900601	K04084 dsbD, dipZ; thioredoxin:protein disulfide reductase [EC:1.8.4.16]
K01159	0,8327	0.00183369820404916	K01159 ruvC; crossover junction endodeoxyribonuclease RuvC [EC:3.1.21.10]
K00980	-2,0235	0.00184129740822979	K00980 tagD; glycerol-3-phosphate cytidylyltransferase [EC:2.7.7.39]
K16090	-2,0338	0.0018539539062347	K16090 fiu; catecholate siderophore receptor
K09760	0,8978	0.00188272460058399	K09760 rmuC; DNA recombination protein RmuC
K03488	-1,5705	0.00193722802958534	K03488 licT, bglG; beta-glucoside operon transcriptional antiterminator
K08218	1,1345	0.00194209469001441	K08218 ampG; MFS transporter, PAT family, beta-lactamase induction signal transducer AmpG

K01772	-1,4231	0.00198468543903841	K01772 hemH, FECH; protoporphyrin/coproporphyrin ferrochelatase [EC:4.98.1.1 4.99.1.9]
K11618	-2,4635	0.00199620061491479	K11618 liaR; two-component system, NarL family, response regulator LiaR
K10984	-2,3116	0.00205980108304513	K10984 agaB; galactosamine PTS system EIIB component [EC:2.7.1.-]
K19355	1,7931	0.00206474013104708	K19355 MAN; mannan endo-1,4-beta-mannosidase [EC:3.2.1.78]
K07114	1,2664	0.00206679501850733	K07114 yfbK; Ca-activated chloride channel homolog
K12528	-1,8026	0.00206759839135539	K12528 xdhD; putative selenate reductase molybdopterin-binding subunit
K05823	-2,4283	0.00207072649822371	K05823 dapL; N-acetyldiaminopimelate deacetylase [EC:3.5.1.47]
K13963	1,4889	0.00209868382719078	K13963 SERPINB; serpin B
K03839	1,0872	0.00218749993138262	K03839 fldA, nifF, isiB; flavodoxin I
K05712	-1,9446	0.00231421060797318	K05712 mhpA; 3-(3-hydroxy-phenyl)propionate hydroxylase [EC:1.14.13.127]
K06929	-1,4930	0.00231823311128428	K06929 K06929; uncharacterized protein
K11737	-1,7619	0.00231829480491789	K11737 cycA; D-serine/D-alanine/glycine transporter
K00677	1,0799	0.00232385116923496	K00677 lpxA; UDP-N-acetylglucosamine acyltransferase [EC:2.3.1.129]
K13252	-2,5778	0.00232708370156038	K13252 ptcA; putrescine carbamoyltransferase [EC:2.1.3.6]
K00034	-2,1558	0.00237157429038024	K00034 gdh; glucose 1-dehydrogenase [EC:1.1.1.47]
K02800	-1,3772	0.00239930287725709	K02800 mtlA, cmtA; mannitol PTS system EIICBA or EIICB component [EC:2.7.1.197]
K02750	-1,5110	0.00240710884907646	K02750 glvC, malP, aglA; alpha-glucoside PTS system EIICB component [EC:2.7.1.208 2.7.1.-]
K01179	0,7915	0.00242409880073553	K01179 E3.2.1.4; endoglucanase [EC:3.2.1.4]
K06988	-1,8912	0.00246347167338655	K06988 fno; 8-hydroxy-5-deazaflavin:NADPH oxidoreductase [EC:1.5.1.40]
K00346	1,2269	0.00248955049808069	K00346 nqrA; Na+-transporting NADH:ubiquinone oxidoreductase subunit A [EC:7.2.1.1]
K04773	0,9342	0.00248978049585489	K04773 sppA; protease IV [EC:3.4.21.-]
K00349	1,2263	0.00249006431469094	K00349 nqrD; Na+-transporting NADH:ubiquinone oxidoreductase subunit D [EC:7.2.1.1]
K00348	1,2234	0.00250023372687562	K00348 nqrC; Na+-transporting NADH:ubiquinone oxidoreductase subunit C [EC:7.2.1.1]
K00350	1,2255	0.00250794996880744	K00350 nqrE; Na+-transporting NADH:ubiquinone oxidoreductase subunit E [EC:7.2.1.1]
K03758	-1,5993	0.00251728319007484	K03758 arcD, lysI, lysP; arginine:ornithine antiporter / lysine permease
K03718	1,1225	0.00259952025516937	K03718 asnC; Lrp/AsnC family transcriptional regulator, regulator for asnA, asnC and gidA
K07559	-1,0492	0.00265722573318755	K07559 kptA; putative RNA 2'-phosphotransferase [EC:2.7.1.-]
K05881	-1,8104	0.00281619128585678	K05881 dhaM; phosphoenolpyruvate---glycerone phosphotransferase subunit DhaM [EC:2.7.1.121]
K03311	-1,6458	0.00284477502713346	K03311 TC.LIVCS; branched-chain amino acid:cation transporter, LIVCS family
K02825	-2,0882	0.0028494090676687	K02825 pyrR; pyrimidine operon attenuation protein / uracil phosphoribosyltransferase [EC:2.4.2.9]

K07313	-1,4547	0.0028597554920023	K07313 pphA; serine/threonine protein phosphatase 1 [EC:3.1.3.16]
K01879	-1,7438	0.00290624120116733	K01879 glyS; glycyl-tRNA synthetase beta chain [EC:6.1.1.14]
K01878	-1,7428	0.00292952800730768	K01878 glyQ; glycyl-tRNA synthetase alpha chain [EC:6.1.1.14]
K07048	-1,1440	0.00300615121873979	K07048 PTER, php; phosphotriesterase-related protein
K15876	1,8026	0.00312154294149278	K15876 nrfH; cytochrome c nitrite reductase small subunit
K02527	1,0109	0.00316608844271145	K02527 kdtA, waaA; 3-deoxy-D-manno-octulosonic-acid transferase [EC:2.4.99.12 2.4.99.13 2.4.99.14 2.4.99.15]
K00009	-1,2841	0.00317115564991543	K00009 mtlD; mannitol-1-phosphate 5-dehydrogenase [EC:1.1.1.17]
K10254	-1,9521	0.00318498011647894	K10254 ohyA, sph; oleate hydratase [EC:4.2.1.53]
K01598	-2,1814	0.00324585940792872	K01598 PPCDC, coaC; phosphopantothenoylcysteine decarboxylase [EC:4.1.1.36]
K01627	0,9607	0.00325584244889321	K01627 kdsA; 2-dehydro-3-deoxyphosphooctonate aldolase (KDO 8-P synthase) [EC:2.5.1.55]
K06148	-1,9678	0.00325937608707222	K06148 ABCC-BAC; ATP-binding cassette, subfamily C, bacterial
K09704	1,2573	0.00327677662644407	K09704 K09704; uncharacterized protein
K07652	-2,2547	0.00329460594043605	K07652 vicK; two-component system, OmpR family, sensor histidine kinase VicK [EC:2.7.13.3]
K03453	0,9283	0.00330784037788658	K03453 TC.BASS; bile acid:Na+ symporter, BASS family
K05245	-1,6359	0.00332397618144989	K05245 caiT; L-carnitine/gamma-butyrobetaine antiporter
K02757	-1,5702	0.00334184873555182	K02757 bglF, bglP; beta-glucoside PTS system EIICBA component [EC:2.7.1.-]
K01467	-1,6634	0.0033708054500363	K01467 ampC; beta-lactamase class C [EC:3.5.2.6]
K01816	-1,1395	0.00337971045139663	K01816 hyi, gip; hydroxypyruvate isomerase [EC:5.3.1.22]
K01173	1,5394	0.00341223574443895	K01173 ENDOG; endonuclease G, mitochondrial
K12138	-1,7036	0.00341483309827708	K12138 hyfC; hydrogenase-4 component C [EC:1.-.-.-]
K03327	0,9440	0.00341942729119034	K03327 SLC47A, MATE, DTX; MATE family, multidrug and toxin extrusion protein
K18887	-1,8041	0.00349363180678927	K18887 efrA, efrE; ATP-binding cassette, subfamily B, multidrug efflux pump
K07480	-2,1739	0.00349987539239421	K07480 insB; insertion element IS1 protein InsB
K06896	-1,7815	0.00354548537557228	K06896 mapP; maltose 6'-phosphate phosphatase [EC:3.1.3.90]
K02768	-1,5503	0.00359501978868764	K02768 fruB; fructose PTS system EIIA component [EC:2.7.1.202]
K07246	-1,5719	0.00362645168101238	K07246 ttuC, dmlA; tartrate dehydrogenase/decarboxylase / D-malate dehydrogenase [EC:1.1.1.93 4.1.1.73 1.1.1.83]
K01232	-1,2368	0.00363460303080668	K01232 glvA; maltose-6'-phosphate glucosidase [EC:3.2.1.122]
K02450	-1,9506	0.00364918435744138	K02450 gspA; general secretion pathway protein A
K02453	-1,6149	0.00367347900931457	K02453 gspD; general secretion pathway protein D
K06920	0,7876	0.0036786743922211	K06920 queC; 7-cyano-7-deazaguanine synthase [EC:6.3.4.20]

K03079	-1,2124	0.00369923601934327	K03079 ulaE, sgaU, sgbU; L-ribulose-5-phosphate 3-epimerase [EC:5.1.3.22]
K07586	-2,1472	0.00380517411163874	K07586 ygaC; uncharacterized protein
K19350	-1,9048	0.00380846196484623	K19350 lsa; lincosamide and streptogramin A transport system ATP-binding/permease protein
K03722	-1,4220	0.0038151446129675	K03722 dinG; ATP-dependent DNA helicase DinG [EC:5.6.2.3]
K03770	0,9680	0.0038707401715848	K03770 ppiD; peptidyl-prolyl cis-trans isomerase D [EC:5.2.1.8]
K03319	-1,7995	0.00388214003976081	K03319 TC.DASS; divalent anion:Na+ symporter, DASS family
K06191	-1,4994	0.00389525192657652	K06191 nrdH; glutaredoxin-like protein NrdH
K19002	-2,0870	0.00390014567735119	K19002 mgs, bgsB; 1,2-diacylglycerol 3-alpha-glucosyltransferase [EC:2.4.1.337]
K09808	0,9471	0.00396465543823513	K09808 lolC_E_F; lipoprotein-releasing system permease protein
K05814	-0,9599	0.00397183664226356	K05814 ugpA; sn-glycerol 3-phosphate transport system permease protein
K08680	-1,6244	0.0039899897904978	K08680 menH; 2-succinyl-6-hydroxy-2,4-cyclohexadiene-1-carboxylate synthase [EC:4.2.99.20]
K03475	-1,2838	0.00406566283888916	K03475 ulaA, sgaT; ascorbate PTS system EIIC component
K01919	-1,2592	0.0040806094622377	K01919 gshA; glutamate--cysteine ligase [EC:6.3.2.2]
K01993	0,9607	0.00413477496055419	K01993 ABC-2.TX; HlyD family secretion protein
K01621	-2,2771	0.00416324811293131	K01621 xfp, xpk; xylulose-5-phosphate/fructose-6-phosphate phosphoketolase [EC:4.1.2.9 4.1.2.22]
K03786	0,9074	0.00423037281022532	K03786 aroQ, qutE; 3-dehydroquinate dehydratase II [EC:4.2.1.10]
K02822	-1,3191	0.00424330466958085	K02822 ulaB, sgaB; ascorbate PTS system EIIB component [EC:2.7.1.194]
K18888	-1,7441	0.00426068838363902	K18888 efrB, efrF; ATP-binding cassette, subfamily B, multidrug efflux pump
K19505	-1,8622	0.00428037873396333	K19505 gfrR; sigma-54 dependent transcriptional regulator, gfr operon transcriptional activator
K06041	0,9138	0.00429599742923987	K06041 kdsD, kpsF; arabinose-5-phosphate isomerase [EC:5.3.1.13]
K00887	-2,1188	0.00430012751522834	K00887 dgkA; undecaprenol kinase [EC:2.7.1.66]
K03587	0,8534	0.00434243234912559	K03587 ftsI; cell division protein FtsI (penicillin-binding protein 3) [EC:3.4.16.4]
K01692	-1,6471	0.00439400358324084	K01692 paaF, echA; enoyl-CoA hydratase [EC:4.2.1.17]
K00239	0,7857	0.00445130747211073	K00239 sdhA, frdA; succinate dehydrogenase flavoprotein subunit [EC:1.3.5.1]
K00012	0,7178	0.0047719210863394	K00012 UGDH, ugd; UDPglucose 6-dehydrogenase [EC:1.1.1.22]
K17810	-2,2524	0.00477564317116926	K17810 asl; D-aspartate ligase [EC:6.3.1.12]
K02259	-2,2732	0.00478064114773117	K02259 COX15, ctaA; heme a synthase [EC:1.17.99.9]
K00283	1,4126	0.00478502010748927	K00283 gcvPB; glycine cleavage system P protein (glycine dehydrogenase) subunit 2 [EC:1.4.4.2]
K00917	-1,6634	0.00478748086455348	K00917 lacC; tagatose 6-phosphate kinase [EC:2.7.1.144]
K12139	-1,6959	0.00483585165411794	K12139 hyfD; hydrogenase-4 component D [EC:1.-.-.-]

K13953	-1,4382	0.00484117166375069	K13953 adhP; alcohol dehydrogenase, propanol-preferring [EC:1.1.1.1]
K07259	0,9101	0.00485157751839216	K07259 dacB; serine-type D-Ala-D-Ala carboxypeptidase/endopeptidase (penicillin-binding protein 4) [EC:3.4.16.4 3.4.21.-]
K03832	1,0245	0.00487380593968144	K03832 tonB; periplasmic protein TonB
K03270	0,9083	0.00499944297858134	K03270 kdsC; 3-deoxy-D-manno-octulosonate 8-phosphate phosphatase (KDO 8-P phosphatase) [EC:3.1.3.45]

Supplementary Table S6: Inferred metabolic pathways from mucosal biopsies' sampled microbiomes

KO	logFC R3 vs pFu	p Value	
K01598	-1,8564	0.00139033934935118	K01598 PPCDC, coaC; phosphopantothenoylcysteine decarboxylase [EC:4.1.1.36]
K11617	-1,8664	0.00157488184890008	K11617 liaS; two-component system, NarL family, sensor histidine kinase LiaS [EC:2.7.13.3]
K09962	-1,5812	0.00167265753406296	K09962 K09962; uncharacterized protein
K01922	-1,7645	0.00181919323342452	K01922 PPCS, COAB; phosphopantothenate---cysteine ligase (ATP) [EC:6.3.2.51]
K03693	-1,6657	0.00205266471815816	K03693 pbp1b; penicillin-binding protein 1B
K01261	-1,5311	0.00213596812419729	K01261 pepA; glutamyl aminopeptidase [EC:3.4.11.7]
K03713	-1,4947	0.00220169614199544	K03713 glnR; MerR family transcriptional regulator, glutamine synthetase repressor
K05362	-1,5737	0.00220820079799254	K05362 murE; UDP-N-acetylmuramoyl-L-alanyl-D-glutamate-L-lysine ligase [EC:6.3.2.7]
K11618	-1,6411	0.00286366528260215	K11618 liaR; two-component system, NarL family, response regulator LiaR
K12554	-1,7928	0.00374055980906847	K12554 murN; alanine adding enzyme [EC:2.3.2.-]
K01819	-1,5468	0.00390159469514134	K01819 E5.3.1.26, lacA, lacB; galactose-6-phosphate isomerase [EC:5.3.1.26]
K04086	-1,7569	0.00531551323096541	K04086 clpL; ATP-dependent Clp protease ATP-binding subunit ClpL
K01635	-1,3398	0.00646760665317095	K01635 lacD; tagatose 1,6-diphosphate aldolase [EC:4.1.2.40]
K12270	-1,4082	0.00653986400715727	K12270 asp3; accessory secretory protein Asp3
K02530	-1,3264	0.00707471222426069	K02530 lacR; DeoR family transcriptional regulator, lactose phosphotransferase system repressor
K12268	-1,3926	0.00717839190530629	K12268 asp1; accessory secretory protein Asp1
K12997	-1,5487	0.0083096873198306	K12997 rgpB; rhamnosyltransferase [EC:2.4.1.-]
K11051	-1,3266	0.00852479692538106	K11051 cylB; multidrug/hemolysin transport system permease protein
K12269	-1,3524	0.00870883136121752	K12269 asp2; accessory secretory protein Asp2
K11050	-1,2801	0.00995768296989311	K11050 cylA; multidrug/hemolysin transport system ATP-binding protein
K12555	-1,3327	0.0105017108763357	K12555 pbp2A; penicillin-binding protein 2A [EC:2.4.99.28 3.4.16.4]
K02246	-1,2428	0.0107677762634884	K02246 comGD; competence protein ComGD
K01220	-1,2425	0.0114294808200567	K01220 E3.2.1.85, lacG; 6-phospho-beta-galactosidase [EC:3.2.1.85]
K01597	-1,1671	0.0118026864740612	K01597 MVD, mvaD; diphosphomevalonate decarboxylase [EC:4.1.1.33]
K00869	-1,1635	0.0121803556282158	K00869 MVK, mvaK1; mevalonate kinase [EC:2.7.1.36]

K06282	0,9274	0.0123659874112782	K06282 hyaA, hybO; hydrogenase small subunit [EC:1.12.99.6]
K02244	-1,1840	0.0125756961910978	K02244 comGB; competence protein ComGB
K13677	-1,2098	0.0127677647228779	K13677 dgs, bgsA; 1,2-diacylglycerol-3-alpha-glucose alpha-1,2-glucosyltransferase [EC:2.4.1.208]
K06281	0,9527	0.0129132410737962	K06281 hyaB, hybC; hydrogenase large subunit [EC:1.12.99.6]
K01042	0,7968	0.0131011597759909	K01042 selA; L-seryl-tRNA(Ser) seleniumtransferase [EC:2.9.1.1]
K05823	-1,4152	0.0132732429545766	K05823 dapL; N-acetyldiaminopimelate deacetylase [EC:3.5.1.47]
K02786	-1,2524	0.013334245433371	K02786 lacF; lactose PTS system EIIA component [EC:2.7.1.207]
K01641	-1,1554	0.0138082295977652	K01641 HMGCS; hydroxymethylglutaryl-CoA synthase [EC:2.3.3.10]
K18474	-1,6918	0.0138235602864617	K18474 fabM; trans-2-decenoyl-[acyl-carrier protein] isomerase [EC:5.3.3.14]
K07038	0,8018	0.0138336053464964	K07038 K07038; inner membrane protein
K00887	-1,1407	0.0140843714760599	K00887 dgkA; undecaprenol kinase [EC:2.7.1.66]
K06198	-1,1730	0.0142527513988817	K06198 coiA; competence protein CoiA
K16371	1,0737	0.0142974326869775	K16371 gatZ-kbaZ; D-tagatose-1,6-bisphosphate aldolase subunit GatZ/KbaZ
K03095	-1,1712	0.0143116283619276	K03095 sprL; SprT-like protein
K00938	-1,1590	0.0148042328002726	K00938 E2.7.4.2, mvaK2; phosphomevalonate kinase [EC:2.7.4.2]
K02248	-1,3160	0.0150724391052503	K02248 comGF; competence protein ComGF
K19304	0,9746	0.0157467358948054	K19304 mepM; murein DD-endopeptidase [EC:3.4.24.-]
K02236	-1,1683	0.0159053927731818	K02236 comC; leader peptidase (prepilin peptidase) / N-methyltransferase [EC:3.4.23.43 2.1.1.-]
K08986	-1,2989	0.0160049818270011	K08986 ycgQ; putative membrane protein
K01185	0,9663	0.0166713500797502	K01185 E3.2.1.17; lysozyme [EC:3.2.1.17]
K07570	-1,1497	0.0166765810055192	K07570 GSP13; general stress protein 13
K18891	-1,3854	0.017748175733409	K18891 patA, rscA, lmrC, satA; ATP-binding cassette, subfamily B, multidrug efflux pump
K18892	-1,3854	0.017748175733409	K18892 patB, rscB, lmrC, satB; ATP-binding cassette, subfamily B, multidrug efflux pump
K05964	1,2986	0.0179058544961708	K05964 citX; holo-ACP synthase [EC:2.7.7.61]
K18918	1,1523	0.0179079900208968	K18918 relB; RHH-type transcriptional regulator, rel operon repressor / antitoxin RelB
K02788	-1,1327	0.0182073757563486	K02788 lacE; lactose PTS system EIICB component [EC:2.7.1.207]
K00054	-1,0531	0.0185126997287107	K00054 mvaA; hydroxymethylglutaryl-CoA reductase [EC:1.1.1.88]
K07778	-1,0094	0.0187136525478573	K07778 desK; two-component system, NarL family, sensor histidine kinase DesK [EC:2.7.13.3]
K08306	1,1030	0.0192288788514405	K08306 mltC; peptidoglycan lytic transglycosylase C [EC:4.2.2.29]
K04027	1,0857	0.0192781207100999	K04027 eutM; ethanolamine utilization protein EutM

K07104	-1,0548	0.0193856882813838	K07104 catE; catechol 2,3-dioxygenase [EC:1.13.11.2]
K05966	1,2364	0.0195282935988133	K05966 citG; triphosphoribosyl-dephospho-CoA synthase [EC:2.4.2.52]
K12678	1,2861	0.019631269129098	K12678 aidA-I, misL; autotransporter family porin
K11392	1,2259	0.0197517535916685	K11392 rsmF; 16S rRNA (cytosine1407-C5)-methyltransferase [EC:2.1.1.178]
K00880	1,2454	0.01980515847753	K00880 lyxK; L-xylulokinase [EC:2.7.1.53]
K19164	1,1744	0.0202191765519536	K19164 ccdA; antitoxin CcdA
K16958	-1,3366	0.0202572905371279	K16958 tcyL; L-cystine transport system permease protein
K16924	-1,2208	0.0204228117122521	K16924 mtsT; energy-coupling factor transport system substrate-specific component
K11707	-1,1528	0.0205825949153435	K11707 troA, mntA, znuA; manganese/zinc/iron transport system substrate-binding protein
K10037	1,2202	0.0209547827308294	K10037 glnP; glutamine transport system permease protein
K03835	1,0657	0.0212927124218684	K03835 mtr; tryptophan-specific transport protein
K00813	0,9283	0.0213111052798708	K00813 aspC; aspartate aminotransferase [EC:2.6.1.1]
K08173	1,2547	0.0217470175173879	K08173 ydfJ; MFS transporter, MHS family, metabolite:H+ symporter
K09798	0,8480	0.0222312410880927	K09798 K09798; uncharacterized protein
K03815	1,3064	0.0223316942462409	K03815
K03605	0,8188	0.0226328024641036	K03605 hyaD, hybD; hydrogenase maturation protease [EC:3.4.23.-]
K03828	1,0731	0.0229132156315113	K03828 yjgM; putative acetyltransferase [EC:2.3.1.-]
K00123	0,6784	0.0229193888463499	K00123 fdoG, fdhF, fdwA; formate dehydrogenase major subunit [EC:1.17.1.9]
K08313	1,1735	0.0232201036720925	K08313 fsaA, mipB; fructose-6-phosphate aldolase 1 [EC:4.1.2.-]
K02478	1,0481	0.0232928160099015	K02478 K02478; two-component system, LytTR family, sensor kinase [EC:2.7.13.3]
K07709	0,9531	0.0234426958684786	K07709 zraS, hydH; two-component system, NtrC family, sensor histidine kinase HydH [EC:2.7.13.3]
K06166	1,0947	0.0238216646112839	K06166 phnG; alpha-D-ribose 1-methylphosphonate 5-triphosphate synthase subunit PhnG [EC:2.7.8.37]
K07710	1,2311	0.0239258330800056	K07710 atoS; two-component system, NtrC family, sensor histidine kinase AtoS [EC:2.7.13.3]
K00769	0,8576	0.0240021778094654	K00769 gpt; xanthine phosphoribosyltransferase [EC:2.4.2.22]
K18843	0,8114	0.0243305152919655	K18843 hicB; antitoxin HicB
K13636	1,3016	0.0243442674772139	K13636 dsdC; LysR family transcriptional regulator, D-serine deaminase activator
K02858	0,8180	0.0244049130447289	K02858 ribB, RIB3; 3,4-dihydroxy 2-butanone 4-phosphate synthase [EC:4.1.99.12]
K10985	1,2591	0.0246500144986455	K10985 agaC; galactosamine PTS system EIIC component
K01093	0,9247	0.0247368080954049	K01093 appA; 4-phytase / acid phosphatase [EC:3.1.3.26 3.1.3.2]
K00313	1,1076	0.0249297100571823	K00313 fixC; electron transfer flavoprotein-quinone oxidoreductase [EC:1.5.5.-]

K06156	1,0631	0.0250729706551096	K06156 gntU; Gnt-I system low-affinity gluconate transporter
K03275	1,2625	0.0251087265905797	K03275 waaO, rfaI; UDP-glucose:(glucosyl)LPS alpha-1,3-glucosyltransferase [EC:2.4.1.-]
K03826	0,6528	0.0251139663557231	K03826 yiaC; putative acetyltransferase [EC:2.3.1.-]
K06078	1,3122	0.0252151434572477	K06078 lpp; murein lipoprotein
K03291	1,2951	0.0253053499861924	K03291 MFS.SET; MFS transporter, SET family, sugar efflux transporter
K07497	0,8396	0.0253307252850819	K07497 K07497; putative transposase
K00917	-0,9528	0.0253368731024572	K00917 lacC; tagatose 6-phosphate kinase [EC:2.7.1.144]
K07307	0,8345	0.0253629440697438	K07307 dmsB; anaerobic dimethyl sulfoxide reductase subunit B
K07640	1,1200	0.0254514936241705	K07640 cpxA; two-component system, OmpR family, sensor histidine kinase CpxA [EC:2.7.13.3]
K08314	1,1384	0.0254645402683836	K08314 fsaB, talC; fructose-6-phosphate aldolase 2 [EC:4.1.2.-]
K05780	1,0882	0.0255161332049245	K05780 phnL; alpha-D-ribose 1-methylphosphonate 5-triphosphate synthase subunit PhnL [EC:2.7.8.37]
K06164	1,0882	0.0256298356112706	K06164 phnI; alpha-D-ribose 1-methylphosphonate 5-triphosphate synthase subunit PhnI [EC:2.7.8.37]
K05781	1,0866	0.0256957368766452	K05781 phnK; putative phosphonate transport system ATP-binding protein
K06163	1,0866	0.0256957368766452	K06163 phnJ; alpha-D-ribose 1-methylphosphonate 5-phosphate C-P lyase [EC:4.7.1.1]
K04835	1,1149	0.0257301905606273	K04835 mal; methylaspartate ammonia-lyase [EC:4.3.1.2]
K11688	-0,9867	0.0260105281191931	K11688 dctP, yiaO; TRAP-type transport system periplasmic protein
K07586	-0,9223	0.0260239915247867	K07586 ygaC; uncharacterized protein
K07648	1,1730	0.0262015528691236	K07648 arcB; two-component system, OmpR family, aerobic respiration control sensor histidine kinase ArcB [EC:2.7.13.3]
K05835	1,0812	0.026222626692911	K05835 rhtC; threonine efflux protein
K10109	1,1134	0.0262766439191022	K10109 malF; maltose/maltodextrin transport system permease protein
K10108	1,1105	0.0262863593489835	K10108 malE; maltose/maltodextrin transport system substrate-binding protein
K03649	1,1234	0.0263287802453985	K03649 mug; double-stranded uracil-DNA glycosylase [EC:3.2.2.28]
K02182	1,2223	0.0266706035827512	K02182 caiC; carnitine-CoA ligase [EC:6.2.1.48]
K07308	1,0601	0.0267024631146601	K07308 dmsC; anaerobic dimethyl sulfoxide reductase subunit C
K18997	1,0417	0.0267775016294787	K18997 cbpM; chaperone modulatory protein CbpM
K07751	1,1397	0.0268443492448461	K07751 pepB; PepB aminopeptidase [EC:3.4.11.23]
K06155	1,0792	0.0269728227257667	K06155 gntT; Gnt-I system high-affinity gluconate transporter
K05523	1,3100	0.0271312961693202	K05523 hchA; D-lactate dehydratase / protein deglycase [EC:4.2.1.130 3.5.1.124]
K05590	1,1372	0.0271674348078975	K05590 srmB; ATP-dependent RNA helicase SrmB [EC:5.6.2.7]
K13283	1,0712	0.0271816754660458	K13283 fieF; ferrous-iron efflux pump FieF

K09477	1,2684	0.0273183991219556	K09477 citT; citrate:succinate antiporter
K03623	1,2142	0.0274215885061375	K03623 yhcO; ribonuclease inhibitor
K00884	1,1570	0.027489436777689	K00884 NAGK; N-acetylglucosamine kinase [EC:2.7.1.59]
K10906	1,3813	0.027662245340677	K10906 recE; exodeoxyribonuclease VIII [EC:3.1.11.-]
K07724	1,3136	0.0277603426051697	K07724 ner, nlp, sfsB; Ner family transcriptional regulator
K10986	1,2370	0.0277858106342758	K10986 agaD; galactosamine PTS system EIID component
K03970	1,2443	0.0278006055743051	K03970 pspB; phage shock protein B
K04770	1,1654	0.027847799781456	K04770 lonH; Lon-like ATP-dependent protease [EC:3.4.21.-]
K00374	0,7976	0.0279120793831784	K00374 narI, narV; nitrate reductase gamma subunit [EC:1.7.5.1 1.7.99.-]
K11534	1,1634	0.0279560742566869	K11534 deoR; DeoR family transcriptional regulator, deoxyribose operon repressor
K07711	1,1727	0.027974139908815	K07711 glrK, qseE; two-component system, NtrC family, sensor histidine kinase GlrK [EC:2.7.13.3]
K03974	1,2384	0.0279966541424304	K03974 pspF; psp operon transcriptional activator
K07662	1,1199	0.028000738467192	K07662 cpxR; two-component system, OmpR family, response regulator CpxR
K19268	1,1101	0.0280271842232442	K19268 glmE, mutE, mamB; methylaspartate mutase epsilon subunit [EC:5.4.99.1]
K09891	1,1290	0.0280284717408964	K09891 K09891; uncharacterized protein
K06165	1,1111	0.0280996039110072	K06165 phnH; alpha-D-ribose 1-methylphosphonate 5-triphosphate synthase subunit PhnH [EC:2.7.8.37]
K12065	1,0921	0.0281205429938743	K12065 traB; conjugal transfer pilus assembly protein TraB
K19235	1,2790	0.0281625429734299	K19235 ybiS; L,D-transpeptidase YbiS
K05802	1,1351	0.0281647722192102	K05802 mscK, kefA, aefA; potassium-dependent mechanosensitive channel
K13695	1,0663	0.0282375018287675	K13695 nlpC; probable lipoprotein NlpC
K08351	1,2855	0.0282554660772607	K08351 bisC; biotin/methionine sulfoxide reductase [EC:1.-.-.-]
K15545	1,1914	0.0282567684129935	K15545 mlc; transcriptional regulator of PTS gene
K05803	1,1335	0.0282858385907107	K05803 nlpI; lipoprotein NlpI
K09893	1,1286	0.0282864765105858	K09893 rraB; regulator of ribonuclease activity B
K15536	1,2128	0.0283203671743794	K15536 cybC; soluble cytochrome b562
K03184	1,1872	0.0283217615076989	K03184 ubiF; 3-demethoxyubiquinol 3-hydroxylase [EC:1.14.99.60]
K18889	1,2258	0.0283665592354045	K18889 mdlA, smdA; ATP-binding cassette, subfamily B, multidrug efflux pump
K07803	1,1912	0.0283930148265848	K07803 zraP; zinc resistance-associated protein
K07337	1,1879	0.0284100037026929	K07337 K07337; penicillin-binding protein activator
K01407	1,2200	0.0284340489147422	K01407 ptrA; protease III [EC:3.4.24.55]

K18842	1,2107	0.0285065120643926	K18842 chpS, chpBI; antitoxin ChpS
K07479	1,1318	0.0285274075486113	K07479 yrdD; putative DNA topoisomerase
K07773	1,1318	0.0285274075486113	K07773 arcA; two-component system, OmpR family, aerobic respiration control protein ArcA
K09897	1,1318	0.0285274075486113	K09897 K09897; uncharacterized protein
K09899	1,1318	0.0285274075486113	K09899 K09899; uncharacterized protein
K19227	1,1318	0.0285274075486113	K19227 sapB; cationic peptide transport system permease protein
K19228	1,1318	0.0285274075486113	K19228 sapC; cationic peptide transport system permease protein
K19230	1,1318	0.0285274075486113	K19230 sapF; cationic peptide transport system ATP-binding protein
K03833	0,6146	0.0285289972277173	K03833 selB, EEFSEC; selenocysteine-specific elongation factor
K03926	0,8190	0.0285704990313231	K03926 cutA; periplasmic divalent cation tolerance protein
K10011	1,2889	0.0286299281382963	K10011 arnA, pmrI; UDP-4-amino-4-deoxy-L-arabinose formyltransferase / UDP-glucuronic acid dehydrogenase (UDP-4-keto-hexauronic acid decarboxylating) [EC:2.1.2.13 1.1.1.305]
K00881	1,2778	0.0286963895051003	K00881 alsK; allose kinase [EC:2.7.1.55]
K02364	1,2560	0.0287985173532847	K02364 entF; L-serine---[L-seryl-carrier protein] ligase [EC:6.3.2.14 6.2.1.72]
K03516	1,3437	0.0288030705416486	K03516 flhE; flagellar protein FlhE
K07014	0,8901	0.0288459135131641	K07014 K07014; uncharacterized protein
K02847	0,9845	0.0288959122435565	K02847 waaL, rfaL; O-antigen ligase [EC:2.4.99.26]
K11750	1,2683	0.028921681149846	K11750 frsA; esterase FrsA [EC:3.1.-.-]
K03573	1,1225	0.0289302060615286	K03573 mutH; DNA mismatch repair protein MutH
K08992	1,1399	0.028933724227138	K08992 lapA; lipopolysaccharide assembly protein A
K19229	1,1269	0.0289478103865747	K19229 sapD; cationic peptide transport system ATP-binding protein
K12152	1,2566	0.028949036253044	K12152 nudJ; phosphatase NudJ [EC:3.6.1.-]
K11704	-0,8374	0.0289495796021691	K11704 mtsA; iron/zinc/manganese/copper transport system substrate-binding protein
K06212	1,1302	0.0289728915115217	K06212 focA; formate transporter
K12661	1,2753	0.0290347875211623	K12661 LRA3, rhmD; L-rhamnonate dehydratase [EC:4.2.1.90]
K02363	1,1710	0.0290612967963585	K02363 entE, dhbE, vibE, mxcE; 2,3-dihydroxybenzoate---[aryl-carrier protein] ligase [EC:6.3.2.14 6.2.1.71]
K19334	1,3069	0.0291069305380049	K19334 tabA; biofilm protein TabA
K07285	1,2511	0.0291667767969726	K07285 slp; outer membrane lipoprotein
K01252	1,2737	0.0291936220696894	K01252 entB, dhbB, vibB, mxcF; bifunctional isochorismate lyase / aryl carrier protein [EC:3.3.2.1 6.3.2.14]
K09894	1,1625	0.0292352227619225	K09894 K09894; uncharacterized protein

K06918	1,0750	0.02927717807658	K06918 K06918; uncharacterized protein
K15974	1,2590	0.0292832319830723	K15974 emrR, mprA; MarR family transcriptional regulator, negative regulator of the multidrug operon emrRAB
K02043	1,1688	0.0293131980047018	K02043 phnF; GntR family transcriptional regulator, phosphonate transport system regulatory protein
K02783	0,8229	0.029319802511188	K02783 srlA; glucitol/sorbitol PTS system EIIC component
K11941	1,3042	0.0293237144708774	K11941 mdoC; glucans biosynthesis protein C [EC:2.1.-.-]
K11751	1,1974	0.0293954421534939	K11751 ushA; 5'-nucleotidase / UDP-sugar diphosphatase [EC:3.1.3.5 3.6.1.45]
K07685	1,1618	0.0294017162028415	K07685 narP; two-component system, NarL family, nitrate/nitrite response regulator NarP
K11258	1,1582	0.0294592295678768	K11258 ilvM; acetolactate synthase II small subunit [EC:2.2.1.6]
K05790	1,1904	0.029488475295214	K05790 wzzE; lipopolysaccharide biosynthesis protein WzzE
K04018	1,0836	0.0295991337348568	K04018 nrfG; formate-dependent nitrite reductase complex subunit NrfG
K07186	1,1586	0.0296390477089859	K07186 smp; membrane protein
K08344	1,2174	0.0296624420744605	K08344 scsB; suppressor for copper-sensitivity B
K00247	1,1264	0.0296763221366474	K00247 frdD; succinate dehydrogenase subunit D
K00073	1,2000	0.0297013461711988	K00073 allD; ureidoglycolate dehydrogenase (NAD+) [EC:1.1.1.350]
K01630	0,8895	0.0297039363529334	K01630 garL; 2-dehydro-3-deoxyglucarate aldolase [EC:4.1.2.20]
K07771	1,1766	0.0297083575083408	K07771 basR; two-component system, OmpR family, response regulator BasR
K03603	1,1696	0.0297355766800916	K03603 fadR; GntR family transcriptional regulator, negative regulator for fad regulon and positive regulator of fabA
K11203	1,1291	0.0298309750253631	K11203 fryC, frvB; fructose-like PTS system EIIC or EIIBC or EIIABC component
K03675	1,2534	0.0298802818103374	K03675 grxB; glutaredoxin 2
K03632	1,1879	0.0299084923745309	K03632 mukB; chromosome partition protein MukB
K10110	1,1356	0.0299235928551384	K10110 malG; maltose/maltodextrin transport system permease protein
K07642	1,0283	0.029930942923846	K07642 baeS, smeS; two-component system, OmpR family, sensor histidine kinase BaeS [EC:2.7.13.3]
K12525	1,3172	0.0299637504549584	K12525 metL; bifunctional aspartokinase / homoserine dehydrogenase 2 [EC:2.7.2.4 1.1.1.3]
K00216	1,2218	0.0299935188948837	K00216 entA; 2,3-dihydro-2,3-dihydroxybenzoate dehydrogenase [EC:1.3.1.28]
K08990	1,0975	0.0299956174400786	K08990 ycjF; putative membrane protein
K04775	1,2891	0.0300485415437217	K04775 ydgD; protease YdgD [EC:3.4.21.-]
K16348	1,2928	0.0300848366215437	K16348 ecnB; entericidin B
K06887	1,3646	0.030087633014104	K06887 K06887; uncharacterized protein
K18890	1,1945	0.0301077649147024	K18890 mdlB, smdB; ATP-binding cassette, subfamily B, multidrug efflux pump
K07283	1,2087	0.0301209676998969	K07283 ydiY; putative salt-induced outer membrane protein

K09909	1,1348	0.0301624654317258	K09909 K09909; uncharacterized protein
K06162	0,9738	0.0301667069347711	K06162 phnM; alpha-D-ribose 1-methylphosphonate 5-triphosphate diphosphatase [EC:3.6.1.63]
K02682	1,1231	0.0301761115798601	K02682 ppdD; prepilin peptidase dependent protein D
K07715	1,1667	0.0302058629776249	K07715 glrR, qseF; two-component system, NtrC family, response regulator GlrR
K09161	1,2998	0.030312977337543	K09161 K09161; uncharacterized protein
K08137	1,2994	0.0303199145671545	K08137 galP; MFS transporter, SP family, galactose:H+ symporter
K03214	1,1422	0.0303660212854396	K03214 yfiF, trmG; RNA methyltransferase, TrmH family [EC:2.1.1.-]
K07689	1,2758	0.0304227741034828	K07689 uvrY, gacA, varA; two-component system, NarL family, invasion response regulator UvrY
K08299	1,2863	0.0304685677513845	K08299 caiD; crotonobetainyl-CoA hydratase [EC:4.2.1.149]
K08160	1,2583	0.0306154675663605	K08160 mdfA, cmr; MFS transporter, DHA1 family, multidrug/chloramphenicol efflux transport protein
K10000	1,1563	0.0306155966788925	K10000 artP; arginine transport system ATP-binding protein [EC:7.4.2.1]
K07274	1,1405	0.0306527269809614	K07274 mipA, ompV; MipA family protein
K03645	1,1565	0.0306828011517157	K03645 seqA; negative modulator of initiation of replication
K03764	1,1565	0.0306828011517157	K03764 metJ; MetJ family transcriptional regulator, methionine regulon repressor
K09904	1,1565	0.0306828011517157	K09904 K09904; uncharacterized protein
K02024	1,1763	0.0306906064870008	K02024 lamB; maltoporin
K08298	1,3513	0.0307090612662726	K08298 caiB; L-carnitine CoA-transferase [EC:2.8.3.21]
K13638	1,3094	0.0307435189867996	K13638 zntR; MerR family transcriptional regulator, Zn(II)-responsive regulator of zntA
K07261	1,0052	0.0307521909233159	K07261 mepA; penicillin-insensitive murein DD-endopeptidase [EC:3.4.24.-]
K02840	1,3909	0.0307865998538465	K02840 waaB, rfaB; UDP-D-galactose:(glucosyl)LPS alpha-1,6-D-galactosyltransferase [EC:2.4.1.-]
K02505	1,1744	0.030844482947037	K02505 hofC; protein transport protein HofC
K19226	1,1551	0.0308529450334218	K19226 sapA; cationic peptide transport system substrate-binding protein
K06159	1,2539	0.0308563587166602	K06159 yojI; multidrug/microcin transport system ATP-binding/permease protein
K03314	1,1175	0.030871854446055	K03314 nhaB; Na+:H+ antiporter, NhaB family
K02415	0,5911	0.0308729961250701	K02415 fliL; flagellar protein FliL
K09901	1,1553	0.0308732553533283	K09901 K09901; uncharacterized protein
K18841	1,1958	0.0308744330941637	K18841 chpB, chpBK; mRNA interferase ChpB [EC:3.1.-.-]
K07674	1,1643	0.0309219515057352	K07674 narQ; two-component system, NarL family, nitrate/nitrite sensor histidine kinase NarQ [EC:2.7.13.3]
K18446	1,2340	0.0309261058613938	K18446 ygiF; triphosphatase [EC:3.6.1.25]
K09997	1,1515	0.0309478096956499	K09997 artI; arginine transport system substrate-binding protein

K03674	1,1538	0.0309740172804212	K03674 grxA; glutaredoxin 1
K02681	1,1537	0.030974979938418	K02681 ppdC; prepilin peptidase dependent protein C
K03753	0,5577	0.0309801058255398	K03753 mobB; molybdopterin-guanine dinucleotide biosynthesis adapter protein
K02466	1,3118	0.0310077113821446	K02466 gutM; glucitol operon activator protein
K12265	1,3137	0.031047300124931	K12265 norW; nitric oxide reductase FlRd-NAD(+) reductase [EC:1.18.1.-]
K03658	1,0034	0.0310473876859196	K03658 helD; DNA helicase IV [EC:5.6.2.4]
K09900	1,1544	0.0310608661012923	K09900 E3.5.1.135; N4-acetylcytidine amidohydrolase [EC:3.5.1.135]
K13014	1,3042	0.0310669312033759	K13014 arnD; undecaprenyl phosphate-alpha-L-ara4FN deformylase [EC:3.5.1.-]
K06006	1,3115	0.0310765445246246	K06006 cpxP, spy; periplasmic protein CpxP/Spy
K08997	1,2455	0.0310846978731032	K08997 SELENOO, selO; protein adenylyltransferase [EC:2.7.7.108]
K07223	0,8061	0.0310999427002527	K07223 yfeX; porphyrinogen peroxidase [EC:1.11.1.-]
K16346	1,2559	0.0311076640501632	K16346 xanQ; xanthine permease XanQ
K14051	1,3012	0.0311206943487522	K14051 gmr, pdeR; c-di-GMP phosphodiesterase Gmr [EC:3.1.4.52]
K19303	1,3131	0.0312095669019147	K19303 mepH; murein DD-endopeptidase [EC:3.4.-.-]
K09917	1,1620	0.031221822224372	K09917 K09917; uncharacterized protein
K12149	1,3025	0.0312658176201476	K12149 dinI; DNA-damage-inducible protein I
K05809	1,1539	0.0313183109694723	K05809 raiA; ribosome-associated inhibitor A
K09907	1,1539	0.0313183109694723	K09907 K09907; uncharacterized protein
K09910	1,1539	0.0313183109694723	K09910 K09910; uncharacterized protein
K16347	1,2782	0.0313281907248402	K16347 ecnA; entericidin A
K02344	1,1534	0.0313785184664663	K02344 holD; DNA polymerase III subunit psi [EC:2.7.7.7]
K03804	1,1534	0.0313785184664663	K03804 mukE; chromosome partition protein MukE
K09896	1,1534	0.0313785184664663	K09896 K09896; uncharacterized protein
K11938	1,3116	0.0313930911947944	K11938 cof; HMP-PP phosphatase [EC:3.6.1.-]
K04021	1,3270	0.0313991325162296	K04021 eutE; aldehyde dehydrogenase
K07347	1,1627	0.0313997493151151	K07347 fimD, fimC, mrkC, htrE, cssD; outer membrane usher protein
K07357	1,3177	0.0313998020483196	K07357 fimB; type 1 fimbriae regulatory protein FimB
K00840	1,2614	0.0314053500272744	K00840 astC; succinylornithine aminotransferase [EC:2.6.1.81]
K18765	1,3128	0.0314340572490728	K18765 csrD; RNase E specificity factor CsrD

K18988	1,2944	0.0314704338877279	K18988 ampH; serine-type D-Ala-D-Ala carboxypeptidase/endopeptidase [EC:3.4.16.4 3.4.21.-]
K07806	1,3017	0.0314781622316022	K07806 arnB, pmrH; UDP-4-amino-4-deoxy-L-arabinose-oxoglutarate aminotransferase [EC:2.6.1.87]
K03633	1,1531	0.0314813455899329	K03633 mukF; chromosome partition protein MukF
K01096	1,1366	0.0315017542026044	K01096 pgpB; phosphatidylglycerophosphatase B [EC:3.1.3.27 3.6.1.75 3.1.3.4 3.6.1.27]
K09470	1,2669	0.0315074987352559	K09470 puuA; gamma-glutamylputrescine synthase [EC:6.3.1.11]
K14762	1,3092	0.0315339187397875	K14762 yibL; ribosome-associated protein
K12963	1,3053	0.0315345905617583	K12963 arnF; undecaprenyl phosphate-alpha-L-ara4N flippase subunit ArnF
K04020	1,3265	0.0315620516380081	K04020 eutD; phosphotransacetylase
K03840	1,3036	0.0315683962542168	K03840 fldB; flavodoxin II
K07781	1,3109	0.0315916452042007	K07781 rcsA; LuxR family transcriptional regulator, capsular biosynthesis positive transcription factor
K07701	1,3170	0.0315985096981977	K07701 dcuS; two-component system, CitB family, sensor histidine kinase DcuS [EC:2.7.13.3]
K05775	1,3003	0.0316004360658835	K05775 malM; maltose operon periplasmic protein
K07264	1,3109	0.0316042832764933	K07264 arnT, pmrK; 4-amino-4-deoxy-L-arabinose transferase [EC:2.4.2.43]
K09892	0,9585	0.0316318147307449	K09892 zapB; cell division protein ZapB
K10124	1,3274	0.031673151321025	K10124 bglH; carbohydrate-specific outer membrane porin
K02507	0,9277	0.0316834449733109	K02507 hofQ; protein transport protein HofQ
K15723	1,3080	0.031687894993312	K15723 syd; SecY interacting protein Syd
K07703	1,3010	0.0317137462237098	K07703 dcuR; two-component system, CitB family, response regulator DcuR
K01071	-1,0522	0.0317199908619155	K01071 MCH; medium-chain acyl-[acyl-carrier-protein] hydrolase [EC:3.1.2.21]
K02562	1,3077	0.0317205076941717	K02562 mtlR; mannitol operon repressor
K18838	1,3373	0.0317294561250702	K18838 cbeA; cytoskeleton bundling-enhancing protein CbeA and related proteins
K19270	1,0101	0.0317428740939245	K19270 hxpA; mannitol-1-/sugar-/sorbitol-6-phosphatase [EC:3.1.3.22 3.1.3.23 3.1.3.50]
K16703	1,3884	0.0317591850772292	K16703 wcaL, amsK, cpsK; colanic acid/amylovoran/stewartan biosynthesis glycosyltransferase WcaL/AmsK/CpsK [EC:2.4.-.-]
K11744	1,2753	0.0317612476765462	K11744 tqsA; AI-2 transport protein TqsA
K00892	1,3068	0.0317892867169717	K00892 gsk; inosine kinase [EC:2.7.1.73]
K02485	1,2500	0.0318144227221552	K02485 rssB, hnr; two-component system, response regulator
K01146	1,3074	0.031820314300385	K01146 xni; protein Xni
K05368	1,3074	0.031820314300385	K05368 fre, ubiB; NAD(P)H-flavin reductase [EC:1.5.1.41]
K12961	1,3036	0.031821140017556	K12961 diaA; DnaA initiator-associating protein
K11743	1,0937	0.0318262274728175	K11743 mdtJ; spermidine export protein MdtJ

K03591	1,1508	0.031852848828762	K03591 ftsN; cell division protein FtsN
K07700	1,3083	0.0318656828108955	K07700 dpiB, citA; two-component system, CitB family, cit operon sensor histidine kinase CitA [EC:2.7.13.3]
K04067	1,1500	0.0318732461065501	K04067 priC; primosomal replication protein N''
K14054	1,3036	0.0318748124212742	K14054 mpaA; murein peptide amidase A
K06073	1,2883	0.0318910696824065	K06073 btuC; vitamin B12 transport system permease protein
K00998	1,1097	0.0319150227976447	K00998 pssA; CDP-diacylglycerol---serine O-phosphatidyltransferase [EC:2.7.8.8]
K11742	1,0935	0.0319161165518892	K11742 mdtI; spermidine export protein MdtI
K09475	1,3120	0.0319427252205686	K09475 ompC; outer membrane pore protein C
K06866	1,1474	0.0319765724159635	K06866 grcA; autonomous glycyl radical cofactor
K12981	1,4014	0.031980455193422	K12981 waaZ, rfaZ; KDO transferase III [EC:2.4.99.-]
K09999	1,1500	0.0319821063390853	K09999 artQ; arginine transport system permease protein
K13919	0,9756	0.0319892815610298	K13919 pduD; propanediol dehydratase medium subunit [EC:4.2.1.28]
K06192	0,7183	0.0320487043495446	K06192 pqiB; paraquat-inducible protein B
K02844	1,0560	0.0320576165062333	K02844 waaG, rfaG; UDP-glucose:(heptosyl)LPS alpha-1,3-glucosyltransferase [EC:2.4.1.-]
K07121	0,9662	0.0320643269866804	K07121 K07121; uncharacterized protein
K07346	1,1234	0.0321014458804728	K07346 fimC; fimbrial chaperone protein
K09998	1,1504	0.0321015784542969	K09998 artM; arginine transport system permease protein
K18837	1,3534	0.0321410570616407	K18837 cbtA; cytoskeleton-binding toxin CbtA and related proteins
K06197	1,2976	0.0321654863901244	K06197 chaB; cation transport regulator
K19222	1,2025	0.0321875883814089	K19222 menI, DHNAT; 1,4-dihydroxy-2-naphthoyl-CoA hydrolase [EC:3.1.2.28]
K11530	1,2849	0.0322218975800069	K11530 lsrG; (4S)-4-hydroxy-5-phosphonooxypentane-2,3-dione isomerase [EC:5.3.1.32]
K11925	1,3033	0.0322227851908087	K11925 sgrR; SgrR family transcriptional regulator
K12500	1,2567	0.0322295110236698	K12500 tesC; thioesterase III [EC:3.1.2.-]
K00427	1,2859	0.0322616260487009	K00427 lldP, lctP; L-lactate permease
K18697	1,1948	0.0322714401349589	K18697 pgpC; phosphatidylglycerophosphatase C [EC:3.1.3.27]
K06074	1,3051	0.032287852509322	K06074 btuD; vitamin B12 transport system ATP-binding protein [EC:7.6.2.8]
K09911	1,1485	0.0323303419554955	K09911 K09911; uncharacterized protein
K05880	1,3150	0.0323513132670397	K05880 dhaR; transcriptional activator for dhaKLM operon
K05811	1,2852	0.0323630541809384	K05811 yfiM; putative lipoprotein

K08154	1,3044	0.0323680653875247	K08154 emrD; MFS transporter, DHA1 family, 2-module integral membrane pump EmrD
K03777	1,0928	0.0323774002345483	K03777 dld; D-lactate dehydrogenase (quinone) [EC:1.1.5.12]
K19048	1,2693	0.0323935598557196	K19048 symE; toxic protein SymE
K09612	1,2977	0.0323972635558952	K09612 iap; alkaline phosphatase isozyme conversion protein [EC:3.4.11.-]
K05708	1,3394	0.0323988855073979	K05708 hcaE, hcaA1; 3-phenylpropionate/trans-cinnamate dioxygenase subunit alpha [EC:1.14.12.19]
K07483	0,6116	0.032416680639078	K07483 K07483; transposase
K05396	0,8089	0.0324207101641649	K05396 dcyD; D-cysteine desulfhydrase [EC:4.4.1.15]
K02680	1,1480	0.0324227617190305	K02680 ppdB; prepilin peptidase dependent protein B
K05594	1,3062	0.0324329817315703	K05594 elaB; ElaB protein
K09906	1,1034	0.0324394314819329	K09906 epmC; elongation factor P hydroxylase [EC:1.14.-.-]
K18657	1,3004	0.0324533292518596	K18657 zapC; cell division protein ZapC
K02504	1,1478	0.0324708649608078	K02504 hofB; protein transport protein HofB
K02679	1,1478	0.0324708649608078	K02679 ppdA; prepilin peptidase dependent protein A
K04753	1,1478	0.0324708649608078	K04753 sufI; suppressor of ftsI
K03478	1,0005	0.0324744317506232	K03478 chbG; chitin disaccharide deacetylase [EC:3.5.1.105]
K06080	1,3043	0.0324807096259452	K06080 rcsF; RcsF protein
K07251	1,3038	0.0325374782963425	K07251 thiK; thiamine kinase [EC:2.7.1.89]
K04017	1,3015	0.0325685144191786	K04017 nrfF; formate-dependent nitrite reductase complex subunit NrfF
K14744	1,3559	0.0325797761374589	K14744 rzpD; prophage endopeptidase [EC:3.4.-.-]
K07757	1,1121	0.0326104248769644	K07757 ybiV; sugar-phosphatase [EC:3.1.3.23]
K03533	1,3331	0.0326125739155297	K03533 torD; TorA specific chaperone
K07309	1,2909	0.0326910376650403	K07309 ynfE; Tat-targeted selenate reductase subunit YnfE [EC:1.97.1.9]
K03425	1,3045	0.0327164640499074	K03425 tatE; sec-independent protein translocase protein TatE
K11926	1,3022	0.0327288480955401	K11926 crl; sigma factor-binding protein Crl
K06957	1,0865	0.0327531616491937	K06957 tmcA; tRNA(Met) cytidine acetyltransferase [EC:2.3.1.193]
K02425	1,3092	0.0327624168593013	K02425 fliZ; regulator of sigma S factor FliZ
K07312	1,3159	0.0327652684214499	K07312 ynfH; Tat-targeted selenate reductase subunit YnfH
K13480	1,3061	0.0328495113799161	K13480 ygeU, xdhC; xanthine dehydrogenase iron-sulfur-binding subunit
K08219	1,3047	0.0328883413348658	K08219 UMF2; MFS transporter, UMF2 family, putative MFS family transporter protein
K01085	1,2139	0.0329073757599118	K01085 agp; glucose-1-phosphatase [EC:3.1.3.10]

K06211	1,1244	0.032913450251478	K06211 nadR; HTH-type transcriptional regulator, transcriptional repressor of NAD biosynthesis genes [EC:2.7.7.1 2.7.1.22]
K12289	1,3043	0.032919999383226	K12289 hofN; pilus assembly protein HofN
K08162	1,2921	0.0329368147806994	K08162 mdtH; MFS transporter, DHA1 family, multidrug resistance protein
K07643	1,3042	0.0329404839027023	K07643 basS; two-component system, OmpR family, sensor histidine kinase BasS [EC:2.7.13.3]
K07310	1,3356	0.0329596216091938	K07310 ynfF; Tat-targeted selenate reductase subunit YnfF [EC:1.97.1.9]
K05709	1,3369	0.0329720242499881	K05709 hcaF, hcaA2; 3-phenylpropionate/trans-cinnamate dioxygenase subunit beta [EC:1.14.12.19]
K03837	1,0676	0.0329732725157495	K03837 sdaC; serine transporter
K03468	1,3039	0.0329856281383535	K03468 aaeB; p-hydroxybenzoic acid efflux pump subunit AaeB
K11736	1,3039	0.0329856281383535	K11736 proY; proline-specific permease ProY
K15548	1,3039	0.0329856281383535	K15548 aaeA; p-hydroxybenzoic acid efflux pump subunit AaeA
K03836	1,3742	0.0329866769775945	K03836 tnaB; low affinity tryptophan permease
K13243	1,3742	0.0329866769775945	K13243 dos, pdeO; c-di-GMP-specific phosphodiesterase [EC:3.1.4.52]
K08225	1,2474	0.0330050242821989	K08225 entS; MFS transporter, ENTS family, enterobactin (siderophore) exporter
K18324	1,3039	0.0330064659669062	K18324 acrD; multidrug efflux pump
K03485	1,3088	0.0330071911008657	K03485 treR; LacI family transcriptional regulator, trehalose operon repressor
K13918	1,2993	0.0330480603181983	K13918 gudX; glucarate dehydratase-related protein
K07269	1,3020	0.0330571224352416	K07269 ytfB; cell division protein YtfB
K05997	1,2984	0.0330714384844479	K05997 sufA; Fe-S cluster assembly protein SufA
K09996	1,3001	0.033087682609194	K09996 artJ; arginine transport system substrate-binding protein
K06141	1,3031	0.0330967403204253	K06141 tsgA; MFS transporter, TsgA protein
K18326	1,3037	0.0331260837554576	K18326 mdtD; MFS transporter, DHA2 family, multidrug resistance protein
K03759	1,3406	0.0331276758624455	K03759 adiC; arginine:agmatine antiporter
K12288	1,3029	0.0331389883161086	K12288 hofM; pilus assembly protein HofM
K07702	1,2988	0.0331595921803784	K07702 dpiA, citB; two-component system, CitB family, response regulator CitB
K07726	0,6422	0.0331682744160146	K07726 K07726; putative transcriptional regulator
K03619	1,0580	0.0331730968377258	K03619 hyaE; hydrogenase-1 operon protein HyaE
K02846	1,2823	0.0331845841743646	K02846 solA; N-methyl-L-tryptophan oxidase [EC:1.5.3.-]
K19236	1,3014	0.0331923960434803	K19236 ycfS; L,D-transpeptidase YcfS
K13631	1,3461	0.0332090537261517	K13631 soxS; AraC family transcriptional regulator, mar-sox-rob regulon activator
K01002	0,7624	0.0332466921001272	K01002 mdoB; phosphoglycerol transferase [EC:2.7.8.20]

K12291	1,3086	0.0332496310546247	K12291 hofP; pilus assembly protein HofP
K05839	1,2970	0.0332582252156301	K05839 hha; haemolysin expression modulating protein
K10748	1,3012	0.033260474666382	K10748 tus, tau; DNA replication terminus site-binding protein
K05517	1,3020	0.0332727139384329	K05517 tsx; nucleoside-specific channel-forming protein
K12974	1,3007	0.0332814457284433	K12974 lpxP; KDO2-lipid IV(A) palmitoleoyltransferase [EC:2.3.1.242]
K03824	0,8373	0.0332839565312943	K03824 yhbS; putative acetyltransferase [EC:2.3.1.-]
K06144	1,3001	0.0332851225181547	K06144 uspB; universal stress protein B
K08319	1,3299	0.0332936060738692	K08319 ltnD; L-threonate 2-dehydrogenase [EC:1.1.1.411]
K16693	1,2974	0.0333402350167256	K16693 wzxE; enterobacterial common antigen flippase
K13920	0,9772	0.0333413077021717	K13920 pduE; propanediol dehydratase small subunit [EC:4.2.1.28]
K11923	1,2965	0.0333506397588353	K11923 cueR; MerR family transcriptional regulator, copper efflux regulator
K08297	1,3443	0.0333581277515041	K08297 caiA; crotonobetainyl-CoA dehydrogenase [EC:1.3.8.13]
K02853	1,2971	0.0333638699060766	K02853 wzyE, rffT; enterobacterial common antigen polymerase [EC:2.4.99.27]
K12582	1,2971	0.0333638699060766	K12582 wecF, rffT; dTDP-N-acetylfucosamine:lipid II N-acetylfucosaminyltransferase [EC:2.4.1.325]
K16704	1,2971	0.0333638699060766	K16704 rffC, wecD; dTDP-4-amino-4,6-dideoxy-D-galactose acyltransferase [EC:2.3.1.210]
K12147	1,3018	0.0333806544030649	K12147 msyB; acidic protein MsyB
K12148	1,2994	0.0334343428250075	K12148 bssS; biofilm regulator BssS
K05713	1,2999	0.0334428625904089	K05713 mhpB; 2,3-dihydroxyphenylpropionate 1,2-dioxygenase [EC:1.13.11.16]
K08682	1,2995	0.0334514040705717	K08682 acpH; acyl carrier protein phosphodiesterase [EC:3.1.4.14]
K02468	1,2973	0.033515199442693	K02468 srlR, gutR; DeoR family transcriptional regulator, glucitol operon repressor
K03971	1,2986	0.0335366388965165	K03971 pspD; phage shock protein D
K11706	-0,8164	0.0335376368633307	K11706 mtsB; iron/zinc/manganese/copper transport system ATP-binding protein
K02080	0,9923	0.0335477617414635	K02080 agaI; putative deaminase/isomerase [EC:3.5.99.-]
K01577	1,1254	0.033560180955976	K01577 oxc; oxalyl-CoA decarboxylase [EC:4.1.1.8]
K07676	1,2988	0.0335704879568703	K07676 rcsD; two-component system, NarL family, sensor histidine kinase RcsD [EC:2.7.13.3]
K13301	1,2988	0.0335704879568703	K13301 secM; secretion monitor
K19162	1,2988	0.0335704879568703	K19162 tomB; hha toxicity modulator TomB
K11924	1,2247	0.0335752998768116	K11924 mntR; DtxR family transcriptional regulator, manganese transport regulator
K05711	1,3344	0.0335900117307977	K05711 hcaB; 2,3-dihydroxy-2,3-dihydrophenylpropionate dehydrogenase [EC:1.3.1.87]
K14588	1,2982	0.033613666302307	K14588 cueO; cuproxidase [EC:1.16.3.4]

K03618	1,1746	0.0336179704659689	K03618 hyaF; hydrogenase-1 operon protein HyaF
K08227	1,2931	0.0336701815891259	K08227 lplT; MFS transporter, LPLT family, lysophospholipid transporter
K12973	1,2717	0.033672622506586	K12973 pagP, crcA; lipid IVA palmitoyltransferase [EC:2.3.1.251]
K04025	1,3155	0.033683309159711	K04025 eutK; ethanolamine utilization protein EutK
K03720	1,1334	0.0337007945898651	K03720 trpR; TrpR family transcriptional regulator, trp operon repressor
K11107	1,2191	0.0337453995635043	K11107 yfaE; ferredoxin
K08987	-0,8677	0.0337830160962816	K08987 K08987; putative membrane protein
K02083	0,8471	0.0338041945589774	K02083 allC; allantoate deiminase [EC:3.5.3.9]
K12975	1,2955	0.0338240983869505	K12975 eptB; KDO II ethanolaminephosphotransferase [EC:2.7.8.42]
K03225	1,1714	0.0338269441907756	K03225 yscQ, sctQ, hrcQ, ssaQ, spaO; type III secretion protein Q
K02381	1,3281	0.033841974627959	K02381 fdrA; FdrA protein
K04081	1,3008	0.0338440585188009	K04081 ibpB; molecular chaperone IbpB
K13683	1,3691	0.0338713631729885	K13683 wcaE; putative colanic acid biosynthesis glycosyltransferase WcaE [EC:2.4.-.-]
K16263	1,2515	0.0338733210609123	K16263 yjeH; amino acid efflux transporter
K09476	1,2839	0.0338955706567319	K09476 ompF; outer membrane pore protein F
K08320	1,2997	0.0339597084056845	K08320 nudG; (d)CTP diphosphatase [EC:3.6.1.65]
K06968	0,9597	0.0339794096338227	K06968 rlmM; 23S rRNA (cytidine2498-2'-O)-methyltransferase [EC:2.1.1.186]
K19160	1,4079	0.0339865076960728	K19160 yafO; mRNA interferase YafO [EC:3.1.-.-]
K08308	1,2954	0.0339885860495808	K08308 mltE, emtA; peptidoglycan lytic transglycosylase E [EC:4.2.2.29]
K14064	1,3003	0.0340126512626888	K14064 uspC; universal stress protein C
K11933	1,2646	0.0340457142606191	K11933 hcr; NADH oxidoreductase Hcr [EC:1.-.-.-]
K02467	1,2993	0.034113392667668	K02467 gutQ; arabinose 5-phosphate isomerase [EC:5.3.1.13]
K07034	0,9160	0.0341197885057341	K07034 GPR1, satP, ATO, ADY2, ACPA; acetate uptake transporter family protein
K07677	1,1259	0.0341517187379631	K07677 rcsC; two-component system, NarL family, capsular synthesis sensor histidine kinase RcsC [EC:2.7.13.3]
K14056	1,2987	0.0341728111395443	K14056 puuR; HTH-type transcriptional regulator, repressor for puuD
K03148	0,6076	0.0341774246814285	K03148 thiF; sulfur carrier protein ThiS adenylyltransferase [EC:2.7.7.73]
K12943	1,2942	0.0341904202877337	K12943 ygeR; lipoprotein YgeR
K16695	1,3742	0.0342447790551878	K16695 wzxC; lipopolysaccharide exporter
K04033	1,2374	0.0343193542617637	K04033 eutR; AraC family transcriptional regulator, ethanolamine operon transcriptional activator
K08318	1,3082	0.0343240494528305	K08318 yihU; 4-hydroxybutyrate dehydrogenase / sulfolactaldehyde 3-reductase [EC:1.1.1.61 1.1.1.373]

K05796	1,2674	0.0343419004457271	K05796 hydN; electron transport protein HydN
K05798	1,2958	0.0343690932751935	K05798 leuO; LysR family transcriptional regulator, transcriptional activator for leuABCD operon
K06039	1,1357	0.0344648886592881	K06039 ychN; uncharacterized protein involved in oxidation of intracellular sulfur
K03805	1,2802	0.0344680882707126	K03805 dsbG; thiol:disulfide interchange protein DsbG
K19161	1,4043	0.0345024852513111	K19161 yafN; antitoxin YafN
K19336	1,3216	0.0345191944056182	K19336 bdcA; cyclic-di-GMP-binding biofilm dispersal mediator protein
K09712	1,1506	0.0345232849033533	K09712 K09712; uncharacterized protein
K09473	1,2976	0.0346378009992707	K09473 puuD; gamma-glutamyl-gamma-aminobutyrate hydrolase [EC:3.5.1.94]
K16066	0,9647	0.0346389469059584	K16066 ydfG; 3-hydroxy acid dehydrogenase / malonic semialdehyde reductase [EC:1.1.1.381 1.1.1.-]
K07349	1,3096	0.0346596336737966	K07349 fimG; minor fimbrial subunit
K16959	-1,2042	0.0346746333457016	K16959 tcyM; L-cystine transport system permease protein
K04058	1,1717	0.0346987668967662	K04058 yscW, sctW; type III secretion protein W
K04653	0,5966	0.0347873334123422	K04653 hypC; hydrogenase expression/formation protein HypC
K07688	1,2211	0.0348143714437923	K07688 fimZ; two-component system, NarL family, response regulator, fimbrial Z protein, FimZ
K08276	1,2149	0.0348441904023723	K08276 eco; ecotin
K14055	1,1425	0.0348661151329689	K14055 uspE; universal stress protein E
K18320	1,2562	0.0348795380924408	K18320 IS15, IS26; transposase, IS6 family
K07490	1,2852	0.0349181094432713	K07490 feoC; ferrous iron transport protein C
K09978	1,0896	0.0349237467506839	K09978 K09978; uncharacterized protein
K11535	0,9980	0.034925720663018	K11535 nupC; nucleoside transport protein
K10857	1,1607	0.03492595583909	K10857 exoX; exodeoxyribonuclease X [EC:3.1.11.-]
K19354	1,3531	0.0349844093314924	K19354 waaH; heptose III glucuronosyltransferase [EC:2.4.1.-]
K19775	1,2876	0.035002843249178	K19775 exuR; GntR family transcriptional regulator, hexuronate regulon transcriptional repressor
K03367	-0,9517	0.035012852402734	K03367 dltA; D-alanine--poly(phosphoribitol) ligase subunit 1 [EC:6.1.1.13]
K05984	1,2826	0.0350266004992035	K05984 cho; excinuclease Cho [EC:3.1.25.-]
K07029	-0,8581	0.0350396606417934	K07029 dagK; diacylglycerol kinase (ATP) [EC:2.7.1.107]
K00835	1,2194	0.0350521871668427	K00835 avtA; valine--pyruvate aminotransferase [EC:2.6.1.66]
K07319	1,3517	0.0350531876639942	K07319 yhdJ; adenine-specific DNA-methyltransferase [EC:2.1.1.72]
K07686	1,2575	0.0351169849708844	K07686 uhpA; two-component system, NarL family, uhpT operon response regulator UhpA

K11607	1,0375	0.0351745183368275	K11607 sitB; manganese/iron transport system ATP-binding protein
K13921	1,1052	0.035206044738714	K13921 pduQ; 1-propanol dehydrogenase
K18478	1,2561	0.0352300854282641	K18478 yihV; sulfofructose kinase [EC:2.7.1.184]
K07454	0,9401	0.0352505724687077	K07454 K07454; putative restriction endonuclease
K01175	1,1011	0.0352657103424733	K01175 ybfF; esterase [EC:3.1.-.-]
K01194	1,0307	0.0352664615457068	K01194 TREH, treA, treF; alpha,alpha-trehalase [EC:3.2.1.28]
K02560	1,0828	0.0353237169859168	K02560 lpxM, msbB; lauroyl-Kdo2-lipid IVA myristoyltransferase [EC:2.3.1.243]
K13641	0,9748	0.0354517485350173	K13641 iclR; IclR family transcriptional regulator, acetate operon repressor
K02345	1,2761	0.035491571601796	K02345 holE; DNA polymerase III subunit theta [EC:2.7.7.7]
K12299	1,3455	0.0355092195368996	K12299 garP; MFS transporter, ACS family, probable galactarate transporter
K04026	0,9354	0.0355334431954745	K04026 eutL; ethanolamine utilization protein EutL
K07109	0,9407	0.0356689692514489	K07109 K07109; uncharacterized protein
K08989	1,0009	0.0356695971729376	K08989 K08989; putative membrane protein
K07799	0,8351	0.0356830945778355	K07799 mdtA; membrane fusion protein, multidrug efflux system
K11216	1,1134	0.035694178876997	K11216 lsrK; autoinducer-2 kinase [EC:2.7.1.189]
K17938	1,1947	0.0357387633877635	K17938 sbmA, bacA; peptide/bleomycin uptake transporter
K00371	0,8510	0.0357630312075073	K00371 narH, narY, nxrB; nitrate reductase / nitrite oxidoreductase, beta subunit [EC:1.7.5.1 1.7.99.-]
K12144	1,2872	0.0357906040363442	K12144 hyfI; hydrogenase-4 component I [EC:1.-.-.-]
K00246	0,7817	0.0358021581395287	K00246 frdC; succinate dehydrogenase subunit C
K10939	1,3608	0.0358264659125038	K10939 acfD; accessory colonization factor AcfD
K16322	1,2426	0.0358645660313272	K16322 pit; low-affinity inorganic phosphate transporter
K15735	1,3359	0.0358994827223054	K15735 csiR; GntR family transcriptional regulator, carbon starvation induced regulator
K07788	0,9379	0.0358995209102609	K07788 mdtB; multidrug efflux pump
K03748	0,9128	0.0359027336994699	K03748 sanA; SanA protein
K07289	0,9320	0.035922128289061	K07289 asmA; AsmA protein
K03567	0,8749	0.035932201795541	K03567 gcvR; glycine cleavage system transcriptional repressor
K16136	1,2708	0.0359655367183695	K16136 malI; LacI family transcriptional regulator, maltose regulon regulatory protein
K06151	0,9481	0.0360211982037441	K06151 E1.1.99.3A; gluconate 2-dehydrogenase alpha chain [EC:1.1.99.3]
K12957	1,1881	0.0360276444022235	K12957 ahr; alcohol/geraniol dehydrogenase (NADP+) [EC:1.1.1.2 1.1.1.183]
K07786	1,3602	0.0360298763612519	K07786 emrY; MFS transporter, DHA2 family, multidrug resistance protein

K16140	1,3602	0.0360298763612519	K16140 uidC, gusC; putative glucuronide porin
K07279	1,3591	0.0360648338653609	K07279 yfaL; autotransporter family porin
K19777	1,3468	0.0361059142324478	K19777 hdeA; acid stress chaperone HdeA
K13735	1,3367	0.0361315848762971	K13735 yeeJ; adhesin/invasin
K02436	1,3126	0.0361466493755175	K02436 gatR; DeoR family transcriptional regulator, galactitol utilization operon repressor
K00245	0,7799	0.0361510954233873	K00245 frdB; succinate dehydrogenase iron-sulfur subunit [EC:1.3.5.1]
K08984	1,1673	0.0361598699496535	K08984 yjdF; putative membrane protein
K16711	1,3676	0.0362187822755494	K16711 wcaM; colanic acid biosynthesis protein WcaM
K05714	1,2727	0.0362249714804729	K05714 mhpC; 2-hydroxy-6-oxonona-2,4-dienedioate hydrolase [EC:3.7.1.14]
K19778	1,3188	0.0362480233405447	K19778 hdeB; acid stress chaperone HdeB
K19335	1,3227	0.0362538125411844	K19335 bdcR; TetR/AcrR family transcriptional regulator, repressor for divergent bdcA
K12066	1,0610	0.0362841292507821	K12066 traK; conjugal transfer pilus assembly protein TraK
K12944	1,3296	0.0362883051327064	K12944 nudI; nucleoside triphosphatase [EC:3.6.1.-]
K08680	0,8669	0.0363168534597875	K08680 menH; 2-succinyl-6-hydroxy-2,4-cyclohexadiene-1-carboxylate synthase [EC:4.2.99.20]
K13684	1,3671	0.0363198952386574	K13684 wcaC; putative colanic acid biosynthesis glycosyltransferase WcaC [EC:2.4.-.-]
K11935	1,2924	0.0363276285020393	K11935 pgaA; biofilm PGA synthesis protein PgaA
K01521	1,2255	0.0363431710645299	K01521 cdh; CDP-diacylglycerol pyrophosphatase [EC:3.6.1.26]
K07796	1,3074	0.0363810909473794	K07796 cusC, silC; outer membrane protein, copper/silver efflux system
K01910	0,7766	0.0364157646670533	K01910 citC; [citrate (pro-3S)-lyase] ligase [EC:6.2.1.22]
K18862	1,3475	0.0364183999076303	K18862 ldrA_B_C_D; small toxic polypeptide LdrA/B/C/D
K12528	1,1984	0.0364278048545864	K12528 xdhD; putative selenate reductase molybdopterin-binding subunit
K02615	1,2646	0.0364659406895269	K02615 paaJ; 3-oxo-5,6-didehydrosuberyl-CoA/3-oxoadipyl-CoA thiolase [EC:2.3.1.223 2.3.1.174]
K10014	1,1895	0.0364946537460832	K10014 hisJ; histidine transport system substrate-binding protein
K16957	-1,1937	0.0365312438678082	K16957 tcyK; L-cystine transport system substrate-binding protein
K11605	1,0339	0.0365365828498629	K11605 sitC; manganese/iron transport system permease protein
K05589	0,6444	0.0365683403797989	K05589 ftsB; cell division protein FtsB
K10975	1,3220	0.0365779722821812	K10975 allP; allantoin permease
K13629	1,3033	0.0365877842485298	K13629 dsdX; D-serine transporter
K19238	1,3495	0.0365911296204355	K19238 pmrD; signal transduction protein PmrD
K11606	1,0325	0.0365937273307537	K11606 sitD; manganese/iron transport system permease protein

K15737	1,3354	0.036600508510027	K15737 csiD; glutarate dioxygenase [EC:1.14.11.64]
K04022	1,3409	0.0366276914620522	K04022 eutG; alcohol dehydrogenase
K02380	0,8547	0.0366519493153567	K02380 fdhE; FdhE protein
K03819	1,3532	0.0366842935120495	K03819 wcaB; putative colanic acid biosynthesis acetyltransferase WcaB [EC:2.3.1.-]
K07355	1,3563	0.0366955866995455	K07355 sfmF; fimbrial-like protein
K12529	1,3467	0.036707889430021	K12529 ygfM; putative selenate reductase FAD-binding subunit
K00370	0,8554	0.03671373916977	K00370 narG, narZ, nxrA; nitrate reductase / nitrite oxidoreductase, alpha subunit [EC:1.7.5.1 1.7.99.-]
K08277	1,3495	0.0367383689799317	K08277 caiF; transcriptional activator CaiF
K08354	1,3495	0.0367383689799317	K08354 phsC; thiosulfate reductase cytochrome b subunit
K05804	1,2456	0.0367747602039215	K05804 rob; AraC family transcriptional regulator, mar-sox-rob regulon activator
K10972	1,3375	0.0368127801120473	K10972 allS; LysR family transcriptional regulator, transcriptional activator of the allD operon
K10555	1,2711	0.0368462008952669	K10555 lsrB; AI-2 transport system substrate-binding protein
K03208	1,2678	0.036853295720229	K03208 wcaI; putative colanic acid biosynthesis glycosyltransferase WcaI
K13620	1,3647	0.0368614824421632	K13620 wcaD; putative colanic acid polymerase
K11391	1,3217	0.0368629722697752	K11391 rlmG; 23S rRNA (guanine1835-N2)-methyltransferase [EC:2.1.1.174]
K01671	1,2999	0.0368663253570868	K01671 yihT; sulfofructosephosphate aldolase [EC:4.1.2.57]
K02430	1,1489	0.036913829146662	K02430 fucR; DeoR family transcriptional regulator, L-fucose operon activator
K16052	0,8636	0.0369278385846748	K16052 ynaI, mscMJ; MscS family membrane protein
K08353	1,3441	0.0369458759447446	K08353 phsB; thiosulfate reductase electron transport protein
K18531	1,3513	0.0369595923169704	K18531 frvR; putative frv operon regulatory protein
K14052	1,2398	0.0370003500146337	K14052 puuP; putrescine importer
K13637	1,3159	0.0370021206353935	K13637 uxuR; GntR family transcriptional regulator, uxu operon transcriptional repressor
K04333	1,3191	0.037007938582609	K04333 csgD; LuxR family transcriptional regulator, csgAB operon transcriptional regulatory protein
K03329	1,3482	0.0370245228099177	K03329 yahN; amino acid exporter
K13632	1,3163	0.0370332174724361	K13632 marA; AraC family transcriptional regulator, mar-sox-rob regulon activator
K12136	1,1855	0.037042248797489	K12136 hyfA; hydrogenase-4 component A [EC:1.-.-.-]
K07136	1,1336	0.0370514049552611	K07136 K07136; uncharacterized protein
K14061	1,3104	0.0370628609907588	K14061 uspF; universal stress protein F
K00885	1,1493	0.0370943194062897	K00885 nanK; N-acylmannosamine kinase [EC:2.7.1.60]
K02855	1,2795	0.0371073617272692	K02855 rhaS; AraC family transcriptional regulator, L-rhamnose operon regulatory protein RhaS

K07358	1,3259	0.0371720281769619	K07358 fimE; type 1 fimbriae regulatory protein FimE
K08279	1,3421	0.0371828016995302	K08279 caiE; carnitine operon protein CaiE
K10558	1,2705	0.0372251055058121	K10558 lsrA, ego; AI-2 transport system ATP-binding protein
K12660	1,3360	0.0372611554322641	K12660 rhmA; 2-dehydro-3-deoxy-L-rhamnonate aldolase [EC:4.1.2.53]
K03535	0,9738	0.0373440740524744	K03535 gudP; MFS transporter, ACS family, glucarate transporter
K10213	1,2079	0.0373594169505813	K10213 rihB; ribosylpyrimidine nucleosidase [EC:3.2.2.8]
K11604	1,0293	0.0373854896383192	K11604 sitA; manganese/iron transport system substrate-binding protein
K15736	1,2167	0.0374068737364338	K15736 lhgO; (S)-2-hydroxyglutarate dehydrogenase [EC:1.1.5.13]
K07714	1,1061	0.0374220663479531	K07714 atoC; two-component system, NtrC family, response regulator AtoC
K07345	1,1315	0.0374320576753295	K07345 fimA; major type 1 subunit fimbrin (pilin)
K09979	0,9713	0.0375302152616662	K09979 K09979; uncharacterized protein
K12143	1,2029	0.0375510613817452	K12143 hyfH; hydrogenase-4 component H
K11201	1,3310	0.037556653937814	K11201 frvA; fructose-like PTS system EIIA component [EC:2.7.1.-]
K08172	1,2092	0.0376550430107581	K08172 shiA; MFS transporter, MHS family, shikimate and dehydroshikimate transport protein
K11264	1,3284	0.0376608740352591	K11264 scpB, mmcD; methylmalonyl-CoA decarboxylase [EC:4.1.1.-]
K03755	1,3381	0.0376704829217952	K03755 adiY; AraC family transcriptional regulator, transcriptional activator of adiA
K10036	0,9728	0.0376712731254893	K10036 glnH; glutamine transport system substrate-binding protein
K12516	1,3574	0.0376968820207984	K12516 bigA; putative surface-exposed virulence protein
K08178	1,1448	0.0377260365003923	K08178 JEN; MFS transporter, SHS family, lactate transporter
K12141	1,0253	0.0377333244213964	K12141 hyfF; hydrogenase-4 component F [EC:1.-.-.-]
K11930	1,3321	0.0377511891274436	K11930 torT; periplasmic protein TorT
K03221	1,2913	0.037754947943876	K03221 yscF, sctF, ssaG, prgI; type III secretion protein F
K11689	-0,8227	0.0378563925480246	K11689 dctQ, yiaM; TRAP-type transport system small permease protein
K12264	1,2949	0.0378760343389321	K12264 norV; anaerobic nitric oxide reductase flavorubredoxin
K09251	1,2064	0.0379409368001111	K09251 patA; putrescine aminotransferase [EC:2.6.1.82]
K08348	1,3023	0.0379772327804888	K08348 fdnG; formate dehydrogenase-N, alpha subunit [EC:1.17.5.3]
K15550	1,2844	0.0379822384654615	K15550 mdtP; outer membrane protein, multidrug efflux system
K04013	1,1587	0.037985423035726	K04013 nrfB; cytochrome c-type protein NrfB
K03825	0,9477	0.0379967374848785	K03825 aaaT; L-phenylalanine/L-methionine N-acetyltransferase [EC:2.3.1.53 2.3.1.-]

K01281	-1,1278	0.038047009093426	K01281 pepX; X-Pro dipeptidyl-peptidase [EC:3.4.14.11]
K15922	1,2487	0.0380642873535862	K15922 yihQ; sulfoquinovosidase [EC:3.2.1.199]
K09890	1,0904	0.0381081515155417	K09890 arfA; alternative ribosome-rescue factor
K06075	0,8782	0.0381788905292444	K06075 slyA; MarR family transcriptional regulator, transcriptional regulator for hemolysin
K04016	1,1571	0.0382239203109248	K04016 nrfE; cytochrome c-type biogenesis protein NrfE
K04080	1,0114	0.0383021085217433	K04080 ibpA; molecular chaperone IbpA
K05778	1,2657	0.0383075676781795	K05778 ynjC; putative thiamine transport system permease protein
K05245	1,1865	0.0383177220611835	K05245 caiT; L-carnitine/gamma-butyrobetaine antiporter
K19776	1,3177	0.0383325742794393	K19776 dgoR; GntR family transcriptional regulator, galactonate operon transcriptional repressor
K11739	1,3562	0.0383338833814979	K11739 nfrA; bacteriophage N4 adsorption protein A
K11531	1,2960	0.0383958926543118	K11531 lsrR; lsr operon transcriptional repressor
K10544	0,9853	0.0384056060659204	K10544 xylH; D-xylose transport system permease protein
K10974	0,9586	0.0384720203967246	K10974 codB; cytosine permease
K07026	1,3174	0.0385635876599666	K07026 E3.1.3.70; mannosyl-3-phosphoglycerate phosphatase [EC:3.1.3.70]
K04334	1,3246	0.0385774823678793	K04334 csgA; major curlin subunit
K05818	1,2552	0.0385979711762322	K05818 mhpR; IclR family transcriptional regulator, mhp operon transcriptional activator
K07314	1,3422	0.038616508797608	K07314 pphB; serine/threonine protein phosphatase 2 [EC:3.1.3.16]
K10984	1,0889	0.0386731497130632	K10984 agaB; galactosamine PTS system EIIB component [EC:2.7.1.-]
K01355	1,3364	0.0386783040941744	K01355 ompT; omptin [EC:3.4.23.49]
K10973	1,3402	0.0386972133463988	K10973 allR; IclR family transcriptional regulator, negative regulator of allantoin and glyoxylate utilization operons
K11922	1,0037	0.0387238974995973	K11922 mngR, farR; GntR family transcriptional regulator, mannosyl-D-glycerate transport/metabolism system repressor
K03740	-0,9571	0.0387357378458066	K03740 dltD; D-alanine transfer protein
K02521	1,0507	0.0387506119581936	K02521 ilvY; LysR family transcriptional regulator, positive regulator for ilvC
K12945	1,3102	0.0387635696973049	K12945 nudK; GDP-mannose pyrophosphatase NudK [EC:3.6.1.-]
K03739	-0,9559	0.0387977696266118	K03739 dltB; membrane protein involved in D-alanine export
K07470	1,3073	0.038842148039818	K07470 sbmC; DNA gyrase inhibitor
K08324	1,2613	0.0388482901968639	K08324 sad; succinate-semialdehyde dehydrogenase [EC:1.2.1.16 1.2.1.24]
K03834	1,0504	0.0388521088634279	K03834 tyrP; tyrosine-specific transport protein
K15836	1,2914	0.0388934832351268	K15836 fhlA; formate hydrogenlyase transcriptional activator
K16509	-0,9120	0.0388972409096462	K16509 spxA; regulatory protein spx

K12290	1,3173	0.0389212944961311	K12290 hofO; pilus assembly protein HofO
K19611	1,1985	0.0389859363283404	K19611 fepA, pfeA, iroN, pirA; ferric enterobactin receptor
K02317	1,3125	0.0390000473638937	K02317 dnaT; DNA replication protein DnaT
K08723	1,3125	0.0390000473638937	K08723 yjjG; pyrimidine 5'-nucleotidase [EC:3.1.3.-]
K15830	1,3055	0.0390113114737225	K15830 hycE; formate hydrogenlyase subunit 5
K11192	1,3139	0.0390142190266709	K11192 murP; N-acetylmuramic acid PTS system EIICB component [EC:2.7.1.192]
K02854	1,2886	0.0390330974745537	K02854 rhaR; AraC family transcriptional regulator, L-rhamnose operon transcriptional activator RhaR
K07229	1,3121	0.0390620179804234	K07229 yqjH; ferric-chelate reductase (NADPH) [EC:1.16.1.9]
K17948	1,2845	0.0391191845024729	K17948 nanM; N-acetylneuraminate epimerase [EC:5.1.3.24]
K12112	1,3166	0.0391516446392301	K12112 ebgC; evolved beta-galactosidase subunit beta
K12151	1,2869	0.0391561887551483	K12151 bhsA; multiple stress resistance protein BhsA
K03477	1,0774	0.0391575247337269	K03477 ulaR; DeoR family transcriptional regulator, ulaG and ulaABCDEF operon transcriptional repressor
K03279	1,1048	0.039159852243935	K03279 waaJ, rfaJ; UDP-glucose:(galactosyl)LPS alpha-1,2-glucosyltransferase [EC:2.4.1.58]
K11929	1,3066	0.0391841273142249	K11929 phoE; outer membrane pore protein E
K07350	1,3052	0.0392010510932099	K07350 fimH; minor fimbrial subunit
K07647	1,2993	0.039221891260119	K07647 torS; two-component system, OmpR family, sensor histidine kinase TorS [EC:2.7.13.3]
K07811	1,3395	0.0392230596231858	K07811 torA; trimethylamine-N-oxide reductase (cytochrome c) [EC:1.7.2.3]
K13255	1,2333	0.0392402212313188	K13255 fhuF; ferric iron reductase protein FhuF
K03276	1,2717	0.0392572087598874	K03276 waaR, waaT, rfaJ; UDP-glucose/galactose:(glucosyl)LPS alpha-1,2-glucosyl/galactosyltransferase [EC:2.4.1.-]
K10708	1,0241	0.039258695771312	K10708 frlB; fructoselysine 6-phosphate deglycase [EC:3.5.-.-]
K19046	1,0456	0.0392991337288357	K19046 casB, cse2; CRISPR system Cascade subunit CasB
K15832	1,3030	0.0393233859662298	K15832 hycG; formate hydrogenlyase subunit 7
K06152	0,9384	0.039328997789496	K06152 E1.1.99.3G; gluconate 2-dehydrogenase gamma chain [EC:1.1.99.3]
K16135	1,1486	0.0393325229218491	K16135 dmlR; LysR family transcriptional regulator, transcriptional activator for dmlA
K00094	1,2400	0.0393954442774572	K00094 E1.1.1.251, gatD; galactitol-1-phosphate 5-dehydrogenase [EC:1.1.1.251]
K02849	1,0085	0.0393976305519979	K02849 waaQ, rfaQ; lipopolysaccharide heptosyltransferase III [EC:2.4.99.25]
K02972	1,3090	0.0395234123785506	K02972 sra; stationary-phase-induced ribosome-associated protein
K11932	1,2507	0.0395290461323171	K11932 uspG; universal stress protein G
K09914	1,0861	0.0395865436805419	K09914 K09914; putative lipoprotein
K05876	1,3091	0.0395950203629988	K05876 trg; methyl-accepting chemotaxis protein III, ribose and galactose sensor receptor

K03207	1,3032	0.0396141637182951	K03207 gmm, nudD, wcaH; GDP-mannose mannosyl hydrolase [EC:3.6.1.-]
K02572	1,1326	0.03968440970649	K02572 napF; ferredoxin-type protein NapF
K11705	-0,7949	0.0397216691129353	K11705 mtsC; iron/zinc/manganese/copper transport system permease protein
K01169	1,3094	0.0397415637442655	K01169 rna; ribonuclease I (enterobacter ribonuclease) [EC:4.6.1.21]
K14261	0,7861	0.0397584403313638	K14261 alaC; alanine-synthesizing transaminase [EC:2.6.1.-]
K04032	0,9314	0.0397642519612599	K04032 eutT; ethanolamine utilization cobalamin adenosyltransferase [EC:2.5.1.154]
K14188	-0,9642	0.0397748441089732	K14188 dltC; D-alanine--poly(phosphoribitol) ligase subunit 2 [EC:6.1.1.13]
K08485	1,0264	0.0398282615721414	K08485 ptsO, npr; phosphocarrier protein NPr
K11144	-0,9137	0.039828263142826	K11144 dnaI; primosomal protein DnaI
K04336	1,3200	0.0398312457063648	K04336 csgC; curli production protein
K11732	1,2905	0.0399010111439493	K11732 pheP; phenylalanine-specific permease
K08163	1,3099	0.0399128268100723	K08163 mdtL; MFS transporter, DHA1 family, multidrug resistance protein
K18141	1,3072	0.0399163811922626	K18141 acrE; membrane fusion protein, multidrug efflux system
K02403	0,9687	0.0400259739265627	K02403 flhD; flagellar transcriptional activator FlhD
K07784	1,2932	0.0400462733649598	K07784 uhpT; MFS transporter, OPA family, hexose phosphate transport protein UhpT
K00932	1,3078	0.0400502639297384	K00932 tdcD; propionate kinase [EC:2.7.2.15]
K03712	1,2209	0.0400546280494512	K03712 marR; MarR family transcriptional regulator, multiple antibiotic resistance protein MarR
K02255	1,3086	0.0400947519137935	K02255 ftnB; ferritin-like protein 2
K15827	1,3086	0.0400947519137935	K15827 hycB; formate hydrogenlyase subunit 2
K15828	1,3086	0.0400947519137935	K15828 hycC; formate hydrogenlyase subunit 3
K15829	1,3086	0.0400947519137935	K15829 hycD; formate hydrogenlyase subunit 4
K15831	1,3086	0.0400947519137935	K15831 hycF; formate hydrogenlyase subunit 6
K15833	1,3086	0.0400947519137935	K15833 hycA; formate hydrogenlyase regulatory protein HycA
K19234	1,3086	0.0400947519137935	K19234 ynhG; L,D-transpeptidase YnhG
K10017	1,1434	0.0400979414287824	K10017 hisP; histidine transport system ATP-binding protein [EC:7.4.2.1]
K02550	1,3395	0.0401091580997821	K02550 glcA; glycolate permease
K13654	1,3188	0.040114522488025	K13654 mcbR; GntR family transcriptional regulator, colanic acid and biofilm gene transcriptional regulator
K02511	1,2288	0.0401289166155692	K02511 hpaX; MFS transporter, ACS family, 4-hydroxyphenylacetate permease
K12113	1,3170	0.0401316193915357	K12113 ebgR; LacI family transcriptional regulator, ebg operon repressor
K02568	1,0353	0.0401542633458934	K02568 napB; nitrate reductase (cytochrome), electron transfer subunit

K15834	1,2771	0.0401704710029351	K15834 hycH; formate hydrogenlyase maturation protein HycH
K08349	1,3018	0.0401886743800973	K08349 fdnH; formate dehydrogenase-N, beta subunit
K08350	1,3018	0.0401886743800973	K08350 fdnI; formate dehydrogenase-N, gamma subunit
K15722	1,3086	0.0402069474101356	K15722 cedA; cell division activator
K19688	1,3074	0.0402303262393548	K19688 bssR; biofilm regulator BssR
K18140	1,3074	0.0402526771961266	K18140 envR, acrS; TetR/AcrR family transcriptional regulator, acrEF/envCD operon repressor
K18142	1,3074	0.0402526771961266	K18142 acrF; multidrug efflux pump
K18530	1,3295	0.0402934163752662	K18530 frvX; putative aminopeptidase FrvX [EC:3.4.11.-]
K07797	1,3389	0.0403000449933653	K07797 emrK; multidrug resistance protein K
K18898	1,3389	0.0403000449933653	K18898 mdtE; membrane fusion protein, multidrug efflux system
K18899	1,3389	0.0403000449933653	K18899 mdtF; multidrug efflux pump
K02402	0,9735	0.0403067952801937	K02402 flhC; flagellar transcriptional activator FlhC
K02446	0,6193	0.0403081942539536	K02446 glpX; fructose-1,6-bisphosphatase II [EC:3.1.3.11]
K06884	1,2010	0.0403199226797237	K06884 K06884; uncharacterized protein
K12140	1,0242	0.0403261425049367	K12140 hyfE; hydrogenase-4 component E [EC:1.-.-.-]
K03838	1,3034	0.0403330107282602	K03838 tdcC; threonine transporter
K04335	1,3036	0.040340344207777	K04335 csgB; minor curlin subunit
K10016	1,1386	0.0404350498601435	K10016 hisQ; histidine transport system permease protein
K02414	0,5802	0.0404661808557487	K02414 fliK; flagellar hook-length control protein FliK
K08194	1,1422	0.0404674965085972	K08194 dgoT; MFS transporter, ACS family, D-galactonate transporter
K01708	0,8818	0.040514724100846	K01708 garD; galactarate dehydratase [EC:4.2.1.42]
K16076	1,3091	0.0405593272521888	K16076 nmpC, ompD; outer membrane porin protein LC
K02386	0,7435	0.0405634490194703	K02386 flgA; flagellar basal body P-ring formation protein FlgA
K03779	0,6624	0.0406222723387922	K03779 ttdA; L(+)-tartrate dehydratase alpha subunit [EC:4.2.1.32]
K04337	1,3070	0.0406745400486308	K04337 csgE; curli production assembly/transport component CsgE
K10556	1,1264	0.0406772715882495	K10556 lsrC; AI-2 transport system permease protein
K00484	1,1059	0.0406919533094147	K00484 hpaC; flavin reductase (NADH) [EC:1.5.1.36]
K10015	1,1371	0.0407017224433197	K10015 hisM; histidine transport system permease protein
K16326	1,2218	0.0407288280445311	K16326 yeiL; CRP/FNR family transcriptional regulator, putaive post-exponential-phase nitrogen-starvation regulator
K03919	0,9749	0.0407609828360877	K03919 alkB; DNA oxidative demethylase [EC:1.14.11.33]

K03490	1,2243	0.0407796320502484	K03490 chbR, celD; AraC family transcriptional regulator, dual regulator of chb operon
K02196	0,8322	0.0407809357917218	K02196 ccmD; heme exporter protein D
K13630	1,3060	0.0408668556910206	K13630 marB; multiple antibiotic resistance protein MarB
K08317	1,2454	0.0408836996842597	K08317 hcxA; hydroxycarboxylate dehydrogenase A [EC:1.1.1.-]
K07592	1,2982	0.0409214783267758	K07592 tdcA; LysR family transcriptional regulator, tdc operon transcriptional activator
K19155	1,2985	0.04092959620121	K19155 yhaV; toxin YhaV [EC:3.1.-.-]
K07130	-0,9046	0.0409938341508524	K07130 kynB; arylformamidase [EC:3.5.1.9]
K18479	1,1980	0.0409960210087604	K18479 yihS; sulfoquinovose isomerase [EC:5.3.1.31]
K07348	1,2928	0.0410112609054154	K07348 fimF; minor fimbrial subunit
K12146	1,3223	0.041062775612272	K12146 hyfR; hydrogenase-4 transcriptional activator
K10038	1,0053	0.0411085340707849	K10038 glnQ; glutamine transport system ATP-binding protein [EC:7.4.2.1]
K05877	1,2849	0.041110617080786	K05877 tap; methyl-accepting chemotaxis protein IV, peptide sensor receptor
K01460	1,1980	0.0411446322156164	K01460 gsp; glutathionylspermidine amidase/synthetase [EC:3.5.1.78 6.3.1.8]
K03459	1,3301	0.0412989027081059	K03459 focB; formate transporter
K07354	1,3301	0.0412989027081059	K07354 sfmD; outer membrane usher protein
K02423	0,9611	0.041303257120143	K02423 fliT; flagellar protein FliT
K06286	-0,9366	0.0413046065542684	K06286 ezrA; septation ring formation regulator
K11936	1,0751	0.0414316247100946	K11936 pgaC, icaA; poly-beta-1,6-N-acetyl-D-glucosamine synthase [EC:2.4.1.-]
K00879	0,7265	0.0415295725794194	K00879 fucK; L-fuculokinase [EC:2.7.1.51]
K05358	1,0552	0.0415634474363732	K05358 quiA; quinate dehydrogenase (quinone) [EC:1.1.5.8]
K00098	1,1172	0.0415981920453075	K00098 idnD; L-idonate 5-dehydrogenase [EC:1.1.1.264]
K03366	-0,6573	0.0416660477308438	K03366 butA, budC; meso-butanediol dehydrogenase / (S,S)-butanediol dehydrogenase / diacetyl reductase [EC:1.1.1.- 1.1.1.76 1.1.1.304]
K01058	0,8101	0.0417648602599466	K01058 pldA; phospholipase A1/A2 [EC:3.1.1.32 3.1.1.4]
K12700	0,9342	0.041792195570547	K12700 rihC; non-specific riboncleoside hydrolase [EC:3.2.2.-]
K11106	1,1632	0.0417963348190383	K11106 ttdT; L-tartrate/succinate antiporter
K14587	1,2779	0.0417982493006322	K14587 sgcE; protein sgcE [EC:5.1.3.-]
K02063	0,9653	0.0418650915090211	K02063 thiP; thiamine transport system permease protein
K10557	1,1364	0.0419485249755578	K10557 lsrD; AI-2 transport system permease protein
K12061	1,0407	0.0419671080887294	K12061 traW; conjugal transfer pilus assembly protein TraW
K19780	1,3237	0.042061454041541	K19780 ralA, rcbA; antisense regulator of RalR protein

K19123	1,0218	0.042071626765389	K19123 casA, cse1; CRISPR system Cascade subunit CasA
K05777	1,2504	0.042111237394095	K05777 ynjB; putative thiamine transport system substrate-binding protein
K05779	1,2504	0.042111237394095	K05779 ynjD; putative thiamine transport system ATP-binding protein
K05817	1,1031	0.0421125698429114	K05817 hcaR; LysR family transcriptional regulator, hca operon transcriptional activator
K14059	0,8365	0.0421687602663981	K14059 int; integrase
K07480	1,2865	0.0421950636756393	K07480 insB; insertion element IS1 protein InsB
K12067	0,7821	0.0422007759710635	K12067 traE; conjugal transfer pilus assembly protein TraE
K13650	1,2954	0.0422635557081809	K13650 mcbA; MqsR-controlled colanic acid and biofilm protein A
K08302	0,6727	0.0423152768710272	K08302 gatY-kbaY; tagatose 1,6-diphosphate aldolase GatY/KbaY [EC:4.1.2.40]
K16324	1,3283	0.0423479785614606	K16324 psuT; putative pseudouridine transporter
K08315	1,2012	0.0423848574357332	K08315 hycI; hydrogenase 3 maturation protease [EC:3.4.23.51]
K03219	1,0454	0.0425472616889311	K03219 yscC, sctC, ssaC; type III secretion protein C
K19156	1,2863	0.04255295677489	K19156 prlF, sohA; antitoxin PrlF
K11940	1,0582	0.0425546017938789	K11940 hspQ; heat shock protein HspQ
K03414	0,7396	0.0426729826530853	K03414 cheZ; chemotaxis protein CheZ
K11102	0,8715	0.042733573343106	K11102 gltP, gltT; proton glutamate symport protein
K03220	0,9666	0.0428008277404389	K03220 yscD, sctD, ssaD; type III secretion protein D
K03228	1,0226	0.0428116518568627	K03228 yscT, sctT, hrcT, ssaT; type III secretion protein T
K17464	1,0919	0.0429408801156698	K17464 dgaA; D-glucosaminate PTS system EIIA component [EC:2.7.1.203]
K12687	1,3573	0.0429428643197162	K12687 flu; antigen 43
K07351	1,2831	0.0429804685114651	K07351 fimI; fimbrial protein
K02193	0,6204	0.0430110992164201	K02193 ccmA; heme exporter protein A [EC:7.6.2.5]
K09802	1,0693	0.0431617963753803	K09802 K09802; uncharacterized protein
K03435	1,0621	0.0431776988063993	K03435 fruR1, fruR; LacI family transcriptional regulator, fructose operon transcriptional repressor
K08368	0,9122	0.0432110494879121	K08368 yaaU; MFS transporter, putative metabolite transport protein
K11745	1,0394	0.0433043305634235	K11745 kefC; glutathione-regulated potassium-efflux system protein KefC
K09021	1,1646	0.0433134499239246	K09021 rutC; aminoacrylate peracid reductase
K07789	0,8596	0.0433197544227596	K07789 mdtC; multidrug efflux pump
K11931	1,1194	0.043396720500512	K11931 pgaB; poly-beta-1,6-N-acetyl-D-glucosamine N-deacetylase [EC:3.5.1.-]
K09016	1,2627	0.0434190922491003	K09016 rutG; putative pyrimidine permease RutG

K12056	1,1750	0.0434728285702098	K12056 traG; conjugal transfer mating pair stabilization protein TraG
K04765	0,8026	0.0434773293194646	K04765 mazG; nucleoside triphosphate diphosphatase [EC:3.6.1.9]
K11746	1,0420	0.043527667504675	K11746 kefF; glutathione-regulated potassium-efflux system ancillary protein KefF
K07664	1,0271	0.0435418244538386	K07664 baeR, smeR; two-component system, OmpR family, response regulator BaeR
K02062	0,9849	0.043605148755588	K02062 thiQ; thiamine transport system ATP-binding protein [EC:7.6.2.15]
K09024	1,0712	0.0436257826648889	K09024 rutF; flavin reductase [EC:1.5.1.-]
K02064	0,9523	0.0436351723271935	K02064 thiB, tbpA; thiamine transport system substrate-binding protein
K19124	0,8882	0.0436686351249452	K19124 casC, cse4; CRISPR system Cascade subunit CasC
K02079	1,2852	0.0436703522939385	K02079 agaA; N-acetylgalactosamine-6-phosphate deacetylase [EC:3.5.1.-]
K18922	1,3152	0.043677891906615	K18922 hokE; protein HokE
K18015	1,3138	0.0436896726009834	K18015 elaD, sseL; deubiquitinase [EC:3.4.22.-]
K03222	1,0264	0.0438251609863159	K03222 yscJ, sctJ, hrcJ, ssaJ; type III secretion protein J
K05785	1,1718	0.0438909814176498	K05785 rfaH; transcriptional antiterminator RfaH
K03212	0,9584	0.0438912978425592	K03212 rumB; 23S rRNA (uracil747-C5)-methyltransferase [EC:2.1.1.189]
K12142	1,2175	0.0439150871500475	K12142 hyfG; hydrogenase-4 component G [EC:1.-.-.-]
K03788	1,1438	0.0439456419009792	K03788 aphA; acid phosphatase (class B) [EC:3.1.3.2]
K14348	1,2096	0.0440335965859406	K14348 lldR; GntR family transcriptional regulator, L-lactate dehydrogenase operon regulator
K03482	1,2633	0.0441058362100066	K03482 yidP; GntR family transcriptional regulator, glv operon transcriptional regulator
K07740	1,1208	0.0441335801106587	K07740 rsd; regulator of sigma D
K11685	1,2825	0.0441561235610094	K11685 stpA; DNA-binding protein StpA
K14062	1,2830	0.0441892696672825	K14062 ompN; outer membrane protein N
K19126	0,9846	0.0441921573525947	K19126 casE, cse3; CRISPR system Cascade subunit CasE
K06149	1,0653	0.0441983030660549	K06149 uspA; universal stress protein A
K02532	0,8336	0.0442078848883986	K02532 lacY; MFS transporter, OHS family, lactose permease
K12145	1,2077	0.0442114008898148	K12145 hyfJ; hydrogenase-4 component J [EC:1.-.-.-]
K01582	0,8517	0.0442350374958335	K01582 E4.1.1.18, ldcC, cadA; lysine decarboxylase [EC:4.1.1.18]
K05501	0,7069	0.0442652295534944	K05501 slmA, ttk; TetR/AcrR family transcriptional regulator
K01699	0,9295	0.0442903800717427	K01699 pduC; propanediol dehydratase large subunit [EC:4.2.1.28]
K01646	0,7433	0.0442986299323901	K01646 citD; citrate lyase subunit gamma (acyl carrier protein)
K05875	1,0487	0.0445122987597192	K05875 tar; methyl-accepting chemotaxis protein II, aspartate sensor receptor

K19000	1,2064	0.0445485427155852	K19000 rof; Rho-binding antiterminator
K09982	1,1349	0.0446381549365519	K09982 K09982; uncharacterized protein
K15547	1,1200	0.0446718431973964	K15547 mdtO; multidrug resistance protein MdtO
K19540	1,2744	0.0447143535135281	K19540 frlA; fructoselysine transporter
K19125	0,9866	0.0447456923979911	K19125 casD, cse5; CRISPR system Cascade subunit CasD
K12138	1,1621	0.0447635935415348	K12138 hyfC; hydrogenase-4 component C [EC:1.-.-.-]
K03227	1,0251	0.0447755099292836	K03227 yscS, sctS, hrcS, ssaS; type III secretion protein S
K14187	1,0021	0.0448076535249175	K14187 tyrA; chorismate mutase / prephenate dehydrogenase [EC:5.4.99.5 1.3.1.12]
K03747	1,0401	0.0448537711016416	K03747 smg; Smg protein
K12063	0,9146	0.0448947106822183	K12063 traC; conjugal transfer ATP-binding protein TraC
K08352	0,9666	0.0449165790785282	K08352 phsA, psrA; thiosulfate reductase / polysulfide reductase chain A [EC:1.8.5.5]
K03607	0,9734	0.0449586792144411	K03607 proQ; ProP effector
K07782	0,9915	0.0449859145829535	K07782 sdiA; LuxR family transcriptional regulator, quorum-sensing system regulator SdiA
K07637	1,1422	0.0450232855072349	K07637 phoQ; two-component system, OmpR family, sensor histidine kinase PhoQ [EC:2.7.13.3]
K08970	1,1688	0.0450782709615707	K08970 rcnA; nickel/cobalt transporter (NicO) family protein
K08161	0,6547	0.0452206712161676	K08161 mdtG, pmrA; MFS transporter, DHA1 family, multidrug resistance protein
K01160	1,2343	0.045221711330439	K01160 rusA; crossover junction endodeoxyribonuclease RusA [EC:3.1.21.10]
K09020	1,1311	0.0452420958710093	K09020 rutB; ureidoacrylate peracid hydrolase [EC:3.5.1.110]
K09824	0,8906	0.0452888268438168	K09824 virK; uncharacterized protein
K08484	0,9233	0.0453407095288725	K08484 ptsP; phosphotransferase system, enzyme I, PtsP [EC:2.7.3.9]
K04568	0,9340	0.0454453876799872	K04568 epmA, poxA; elongation factor P--(R)-beta-lysine ligase [EC:6.3.1.-]
K05363	-1,2850	0.0454529897853951	K05363 murM; serine/alanine adding enzyme [EC:2.3.2.10]
K15549	1,1103	0.0455388392522021	K15549 mdtN; membrane fusion protein, multidrug efflux system
K03226	1,0177	0.045570774179871	K03226 yscR, sctR, hrcR, ssaR; type III secretion protein R
K02194	0,6123	0.0456065178191182	K02194 ccmB; heme exporter protein B
K03112	1,1810	0.045745019381712	K03112 damX; DamX protein
K02598	0,7048	0.0458442813340951	K02598 nirC; nitrite transporter
K05982	0,9900	0.0459112873973918	K05982 nfi; deoxyribonuclease V [EC:3.1.21.7]
K00694	0,7518	0.0459339000268795	K00694 bcsA; cellulose synthase (UDP-forming) [EC:2.4.1.12]
K19734	1,2094	0.0460331949863417	K19734 expR; LuxR family transcriptional regulator, quorum-sensing system regulator ExpR

K02336	1,1246	0.0460492325473216	K02336 polB; DNA polymerase II [EC:2.7.7.7]
K07661	1,0776	0.0460865910710316	K07661 rstA; two-component system, OmpR family, response regulator RstA
K14057	1,2664	0.0461113853700975	K14057 abgR; LysR family transcriptional regulator, regulator of abg operon
K05787	0,9934	0.0461190699041544	K05787 hupA; DNA-binding protein HU-alpha
K18919	1,3108	0.0462059407473558	K18919 hokC_D; protein HokC/D
K07675	1,0852	0.0462188868815085	K07675 uhpB; two-component system, NarL family, sensor histidine kinase UhpB [EC:2.7.13.3]
K02076	-0,7433	0.0462271011906249	K02076 zurR, zur; Fur family transcriptional regulator, zinc uptake regulator
K13771	0,6648	0.0462372920335999	K13771 nsrR; Rrf2 family transcriptional regulator, nitric oxide-sensitive transcriptional repressor
K18920	1,3157	0.0463063102377389	K18920 hokA; protein HokA
K09023	1,1535	0.0464036701373413	K09023 rutD; aminoacrylate hydrolase [EC:3.5.1.-]
K13069	1,1046	0.046445084122108	K13069 E2.7.7.65; diguanylate cyclase [EC:2.7.7.65]
K03721	0,9834	0.0465610635929828	K03721 tyrR; transcriptional regulator of aroF, aroG, tyrA and aromatic amino acid transport
K07306	0,7813	0.0466197997437213	K07306 dmsA; anaerobic dimethyl sulfoxide reductase subunit A [EC:1.8.5.3]
K03669	0,9687	0.0466244292384768	K03669 mdoH; membrane glycosyltransferase [EC:2.4.1.-]
K07639	1,0778	0.0466341285238761	K07639 rstB; two-component system, OmpR family, sensor histidine kinase RstB [EC:2.7.13.3]
K02197	0,6107	0.0466896028184844	K02197 ccmE; cytochrome c-type biogenesis protein CcmE
K10711	1,0607	0.0467204495023137	K10711 frlR; GntR family transcriptional regulator, frlABCD operon transcriptional regulator
K07172	0,7657	0.0467632130672897	K07172 mazE, chpAI; antitoxin MazE
K05819	1,1388	0.046864266788806	K05819 mhpT; MFS transporter, AAHS family, 3-hydroxyphenylpropionic acid transporter
K02245	-0,9127	0.0469460377862185	K02245 comGC; competence protein ComGC
K06858	1,1587	0.0470012729084338	K06858 btuF; vitamin B12 transport system substrate-binding protein
K02195	0,6087	0.0470414586298018	K02195 ccmC; heme exporter protein C
K10013	1,0853	0.0470514018210159	K10013 argT; lysine/arginine/ornithine transport system substrate-binding protein
K08326	1,2942	0.0471033975885076	K08326 ypdF; aminopeptidase [EC:3.4.11.-]
K16842	1,0877	0.0471038973112905	K16842 hpxB; allantoinase [EC:3.5.2.5]
K09857	0,7827	0.0473138754654776	K09857 K09857; uncharacterized protein
K06948	-0,8755	0.0473628442969792	K06948 yqeH; 30S ribosome assembly GTPase
K05851	0,9973	0.0473860877477905	K05851 cyaA; adenylate cyclase, class 1 [EC:4.6.1.1]
K00230	0,7298	0.0474249445025934	K00230 hemG; menaquinone-dependent protoporphyrinogen oxidase [EC:1.3.5.3]
K02394	0,6948	0.0475592255762105	K02394 flgI; flagellar P-ring protein FlgI

K16692	1,0558	0.0475660779154648	K16692 etk-wzc; tyrosine-protein kinase Etk/Wzc [EC:2.7.10.3]
K16074	0,9724	0.0477685447246351	K16074 zntB; zinc transporter
K09018	1,1212	0.0478305982227516	K09018 rutA; pyrimidine oxygenase [EC:1.14.99.46]
K00113	0,7432	0.047986564138637	K00113 glpC; glycerol-3-phosphate dehydrogenase subunit C
K05939	0,9367	0.0480098890416511	K05939 aas; acyl-[acyl-carrier-protein]-phospholipid O-acyltransferase / long-chain-fatty-acid--[acyl-carrier-protein] ligase [EC:2.3.1.40 6.2.1.20]
K03972	1,0578	0.0480627365313288	K03972 pspE; phage shock protein E
K06222	1,0229	0.0480659413686045	K06222 dkgB; 2,5-diketo-D-gluconate reductase B [EC:1.1.1.346]
K04065	1,0302	0.0481061971898665	K04065 osmY; hyperosmotically inducible periplasmic protein
K07184	1,0205	0.0482500880693557	K07184 ygiM; SH3 domain protein
K02439	0,9812	0.0482722505843741	K02439 glpE; thiosulfate sulfurtransferase [EC:2.8.1.1]
K03229	1,0011	0.0482917474938581	K03229 yscU, sctU, hrcU, ssaU; type III secretion protein U
K00389	0,9022	0.048368409949936	K00389 yidH; inner membrane protein YidH
K06214	1,1496	0.0484355651180907	K06214 csgG; curli production assembly/transport component CsgG
K06896	-1,0220	0.0484381863444624	K06896 mapP; maltose 6'-phosphate phosphatase [EC:3.1.3.90]
K00373	0,7528	0.0485400180297381	K00373 narJ, narW; nitrate reductase molybdenum cofactor assembly chaperone NarJ/NarW
K10537	0,9560	0.0485597892089273	K10537 araF; L-arabinose transport system substrate-binding protein
K01531	0,5312	0.0485673720547548	K01531 mgtA, mgtB; P-type Mg2+ transporter [EC:7.2.2.14]
K11066	1,0252	0.0485685770612364	K11066 amiD; N-acetylmuramoyl-L-alanine amidase [EC:3.5.1.28]
K01825	1,0948	0.0485789339375244	K01825 fadB; 3-hydroxyacyl-CoA dehydrogenase / enoyl-CoA hydratase / 3-hydroxybutyryl-CoA epimerase / enoyl-CoA isomerase [EC:1.1.1.35 4.2.1.17 5.1.2.3 5.3.3.8]
K04338	1,2358	0.0486863264916556	K04338 csgF; curli production assembly/transport component CsgF
K04019	0,8337	0.0488347696936935	K04019 eutA; ethanolamine utilization protein EutA
K05774	0,9713	0.0488668537026559	K05774 phnN; ribose 1,5-bisphosphokinase [EC:2.7.4.23]
K13892	0,9416	0.0490019561397772	K13892 gsiA; glutathione transport system ATP-binding protein
K09806	0,8487	0.0491308738216472	K09806 ubiK; ubiquinone biosynthesis accessory factor UbiK
K06175	0,8101	0.0492263738123409	K06175 truC; tRNA pseudouridine65 synthase [EC:5.4.99.26]
K05685	0,8124	0.0493175651792517	K05685 macB; macrolide transport system ATP-binding/permease protein [EC:7.6.2.-]
K13926	1,0553	0.0493578207583882	K13926 rbbA; ribosome-dependent ATPase
K06146	1,2818	0.0493951552572282	K06146 idnR, gntH; LacI family transcriptional regulator, gluconate utilization system Gnt-II transcriptional activator
K12972	0,8648	0.0494129855986965	K12972 ghrA; glyoxylate/hydroxypyruvate reductase [EC:1.1.1.79 1.1.1.81]
K02784	0,9931	0.0494235906607503	K02784 ptsH; phosphocarrier protein HPr

K02496	0,7771	0.0494248704443782	K02496 hemX; uroporphyrin-III C-methyltransferase [EC:2.1.1.107]
K09923	0,9397	0.0494358980328907	K09923 K09923; uncharacterized protein
K05526	1,0845	0.049466586963141	K05526 astE; succinylglutamate desuccinylase [EC:3.5.1.96]
K02565	1,1462	0.0494730408327961	K02565 nagC; N-acetylglucosamine repressor
K07804	1,3424	0.0495446343763759	K07804 pagC; putatice virulence related protein PagC
K06946	1,2797	0.049558609407918	K06946 K06946; uncharacterized protein
K06157	1,3006	0.0495800842689508	K06157 idnT; Gnt-II system L-idonate transporter
K12962	1,1227	0.0495842372599701	K12962 arnE; undecaprenyl phosphate-alpha-L-ara4N flippase subunit ArnE
K07343	0,6514	0.0496549086533154	K07343 tfoX; DNA transformation protein and related proteins
K03445	1,0527	0.0496556493519005	K03445 nepI; MFS transporter, DHA1 family, purine ribonucleoside efflux pump
K01147	0,6261	0.049701900279807	K01147 rnb; exoribonuclease II [EC:3.1.13.1]
K07400	0,9402	0.0497705693424312	K07400 nfuA; Fe/S biogenesis protein NfuA
K09131	0,8271	0.0497759174001549	K09131 K09131; uncharacterized protein
K09976	-0,8644	0.0498027476518067	K09976 K09976; uncharacterized protein
K02742	1,0249	0.0498597353732662	K02742 sprT; SprT protein
K02298	0,8825	0.049877449908769	K02298 cyoB; cytochrome o ubiquinol oxidase subunit I [EC:7.1.1.3]
K03304	1,0797	0.0499070322169226	K03304 tehA; tellurite resistance protein
K18325	1,2579	0.0499198219254599	K18325 ramA; AraC family of transcriptional regulator, multidrug resistance transcriptional activator
K10040	-1,0337	0.0499474455373451	K10040 peb1B, glnP, glnM; aspartate/glutamate/glutamine transport system permease protein
K10539	0,8990	0.0499856606512856	K10539 araG; L-arabinose transport system ATP-binding protein [EC:7.5.2.12]
